# Supplementary material for: Cultural and Ethical Barriers to Cancer Treatment in Nursing Homes and Educational Strategies: A Scoping Review
Source: Cancers (Basel). 2021 Jul 14;13(14):3514. doi: 10.3390/cancers13143514 (PMC8305927; doi:10.3390/cancers13143514)
Supplement: Supplementary file 1 [file cancers-13-03514-s001.zip › cancers-1261082-supplementary.pdf]

Supplementary Materials: Cultural and Ethical Barriers to Cancer Treatment in Nursing Homes and Educational Strategies: A Scoping Review

Cynthia Filteau, Arnaud Simeone, Christine Ravot, David Dayde and Claire Falandry

Table S1. Search strategies for scoping review.

Database	Number of Studies	Search Query
PubMed	10	"Neoplasms/education"[Mesh] OR "Neoplasms"[Mesh]) AND ("Nursing Homes"[Mesh] OR "Homes for the Aged"[Mesh] OR "Assisted Living Facilities"[Mesh]) AND ("Education, Professional"[Mesh] OR "Staff Development"[Mesh] OR "Inservice Training"[Mesh] OR "Health Knowledge, Attitudes, Practice"[Mesh] OR "Health Communication"[Mesh])
	22	("neoplasm" OR "cancer" OR "oncology" OR "chemotherapy") AND ("information" OR "education in cancer" OR "training" OR "inservice training" OR "course" OR "health care communication" OR "improving knowledge) AND (" AND caregiver AND "OR " AND health professionals AND "OR " AND nurse AND "OR " AND staff AND "OR " AND doctor AND ")) AND (" AND nursing home AND "OR " AND home hospice AND "OR " AND home care AND "OR " AND hospice care AND "OR " AND homes for the aged AND "OR residential facilities" OR "assisted living facilities" OR "old age home")
	24	(((((aged[MeSH Terms]) AND (neoplasms[MeSH Terms])) AND (nursing homes[MeSH Terms]))) NOT (care, palliative[MeSH Terms])) AND (education)
	190	((((aged[MeSH Terms]) AND (neoplasms[MeSH Terms])) AND (nursing homes[MeSH Terms]))) NOT (care, palliative[MeSH Terms])
	3	(("continuing education"[Title/Abstract]) AND ("nursing"[Title/Abstract])) AND ("cooperative learning"[Title/Abstract])
	12	((continuing education[Title/Abstract]) AND (nursing[Title/Abstract])) AND (participatory[Title/Abstract])
	2	(("continuing education"[Title/Abstract]) AND ("nursing"[Title/Abstract])) AND (Collaborative learning[Title/Abstract])
Embase	238	'malignant neoplasm'/exp AND ('home for the aged'/exp OR 'old age homes'/exp OR 'home* for the aged':ti,ab,kw OR 'housing* for the elderly':ti,ab,kw OR 'old age home*':ti,ab,kw OR 'old people home*':ti,ab,kw OR ('aged'/exp AND ('nursing home'/exp OR 'assisted living facility'/exp))) AND [embase]/lim NOT ([embase]/lim AND [medline]/lim)
	28	'malignant neoplasm'/exp AND 'aged'/exp AND ('paramedical education'/exp OR 'paramedical education':ti,ab,kw OR 'paramedical training':ti,ab,kw OR 'nurse training':ti,ab,kw OR 'nurse education':ti,ab,kw OR ('continuing education'/exp AND 'paramedical personnel'/exp))
CINAHL	76	MH (“neoplasms+”) AND ((MH (“housing for the elderly”) OR TI (“housing* for the elderly” OR “home* for the aged” OR “old age home*” OR “old people home*”) OR AB (“housing* for the elderly” OR “home* for the aged” OR “old age home*” OR “old people home*”)) OR (MH (“aged+”) AND (MH (“nursing homes+”) OR TI (“nursing home*” OR “assisted living facility*”) OR AB (“nursing home*” OR “assisted living facility*”))))
	25	MH (“neoplasms+” AND “aged+”) AND (MH (“education, nursing+” OR “education, allied health+”) OR AB (“nursing education” OR “nursing training” OR “paramedical education” OR “paramedical training”) OR TI (“nursing education” OR “nursing training” OR “paramedical education” OR “paramedical training”) OR (MH (“education, continuing+”) AND MH (“Allied health personnel+” OR “nurses+”)))

Table S2. Preferred Reporting Items for Systematic reviews and Meta-Analyses extension for Scoping Reviews (PRISMA-ScR) Checklist.

SECTION	ITEM	PRISMA-ScR CHECKLIST ITEM	REPORTED ON PAGE #
		TITLE	
Title	1	Identify the report as a scoping review.	1
		ABSTRACT	
Structured summary	2	Provide a structured summary that includes (as applicable): background, objectives, eligibility criteria, sources of evidence, charting methods, results, and conclusions that relate to the review questions and objectives.	2
		INTRODUCTION	
Rationale	3	Describe the rationale for the review in the context of what is already known. Explain why the review questions/objectives lend themselves to a scoping review approach.	3
Objectives	4	Provide an explicit statement of the questions and objectives being addressed with reference to their key elements (e.g., population or participants, concepts, and context) or other relevant key elements used to conceptualize the review questions and/or objectives.	3
		METHODS	
Protocol and registration	5	Indicate whether a review protocol exists; state if and where it can be accessed (e.g., a Web address); and if available, provide registration information, including the registration number.	3
Eligibility criteria	6	Specify characteristics of the sources of evidence used as eligibility criteria (e.g., years considered, language, and publication status), and provide a rationale.	3
Information sources*	7	Describe all information sources in the search (e.g., databases with dates of coverage and contact with authors to identify additional sources), as well as the date the most recent search was executed.	3
Search	8	Present the full electronic search strategy for at least 1 database,	Supplementary materials, Table S1

SECTION	ITEM	PRISMA-ScR CHECKLIST ITEM	REPORTED ON PAGE #
		including any limits used, such that it could be repeated.	
Selection of sources of evidence†	9	State the process for selecting sources of evidence (i.e., screening and eligibility) included in the scoping review.	3
Data charting process‡	10	Describe the methods of charting data from the included sources of evidence (e.g., calibrated forms or forms that have been tested by the team before their use, and whether data charting was done independently or in duplicate) and any processes for obtaining and confirming data from investigators.	3
Data items	11	List and define all variables for which data were sought and any assumptions and simplifications made.	4
Critical appraisal of individual sources of evidence§	12	If done, provide a rationale for conducting a critical appraisal of included sources of evidence; describe the methods used and how this information was used in any data synthesis (if appropriate).	4
Synthesis of results	13	Describe the methods of handling and summarizing the data that were charted.	4
RESULTS			
Selection of sources of evidence	14	Give numbers of sources of evidence screened, assessed for eligibility, and included in the review, with reasons for exclusions at each stage, ideally using a flow diagram.	5
Characteristics of sources of evidence	15	For each source of evidence, present characteristics for which data were charted and provide the citations.	5-14 and Supplementary materials, Table S4-S10
Critical appraisal within sources of evidence	16	If done, present data on critical appraisal of included sources of evidence (see item 12).	Supplementary materials, Table S4-S10
Results of individual sources of evidence	17	For each included source of evidence, present the relevant data that were charted that relate to the review questions and objectives.	5-14
Synthesis of results	18	Summarize and/or present the charting results as they relate to the review questions and objectives.	5-14
DISCUSSION			

SECTION	ITEM	PRISMA-ScR CHECKLIST ITEM	REPORTED ON PAGE #
Summary of evidence	19	Summarize the main results (including an overview of concepts, themes, and types of evidence available), link to the review questions and objectives, and consider the relevance to key groups.	14-16
Limitations	20	Discuss the limitations of the scoping review process.	15-16
Conclusions	21	Provide a general interpretation of the results with respect to the review questions and objectives, as well as potential implications and/or next steps.	16
FUNDING			
Funding	22	Describe sources of funding for the included sources of evidence, as well as sources of funding for the scoping review. Describe the role of the funders of the scoping review.	16

List S3: All records screened listed, after duplicates removal (*n* = 605).

1. Akdeniz, M.; Hahnel, E.; Ulrich, C.; Blume-Peytavi, U.; Kottner, J. Prevalence and Associated Factors of Skin Cancer in Aged Nursing Home Residents: A Multicenter Prevalence Study. *PLoS ONE* 2019, 14, e0215379, doi:10.1371/journal.pone.0215379.
2. Blytt, K.M.; Selbæk, G.; Drageset, J.; Natvig, G.K.; Husebo, B.S. Comorbid Dementia and Cancer in Residents of Nursing Homes: Secondary Analyses of a Cross-Sectional Study. Cancer Nursing 2018, 41, E13–E20, doi:10.1097/NCC.0000000000000478.
3. Boyd, M.; Frey, R.; Balmer, D.; Robinson, J.; McLeod, H.; Foster, S.; Slark, J.; Gott, M. End of Life Care for Long-Term Care Residents with Dementia, Chronic Illness and Cancer: Prospective Staff Survey. BMC Geriatr **2019**, *19*, 137, doi:10.1186/s12877-019-1159-2.
4. Bradley, C.J.; Clement, J.P.; Lin, C. Absence of Cancer Diagnosis and Treatment in Elderly Medicaid-Insured Nursing Home Residents. JNCI: Journal of the National Cancer Institute **2008**, *100*, 21–31, doi:10.1093/jnci/djm271.
5. Brink, P. Examining Do-Not-Resuscitate Orders Among Newly Admitted Residents of Long-Term Care Facilities. Palliat Care **2014**, *8*, 1–6, doi:10.4137/PCRT.S13042.
6. Buchanan, I.A.; Donoho, D.A.; Patel, A.; Lin, M.; Wen, T.; Ding, L.; Giannotta, S.L.; Mack, W.J.; Attenello, F. Predictors of Surgical Site Infection After Nonemergent Craniotomy: A Nationwide Readmission Database Analysis. World Neurosurg 2018, 120, e440–e452, doi:10.1016/j.wneu.2018.08.102.
7. Butler, C.C.; Lau, M.; Gillespie, D.; Owen-Jones, E.; Lown, M.; Wootton, M.; Calder, P.C.; Bayer, A.J.; Moore, M.; Little, P.; et al. Effect of Probiotic Use on Antibiotic Administration Among Care Home Residents: A Randomized Clinical Trial. *JAMA* **2020**, *324*, 47–56, doi:10.1001/jama.2020.8556.
8. Chan, T.Y.; Foo, C.C.; Law, W.L.; Lo, O. Outcomes of Colorectal Cancer Surgery in the Nonagenarians: 20-Year Result from a Tertiary Center. BMC Surg **2019**, *19*, 155, doi:10.1186/s12893-019-0623-4.
9. Clement, J.P.; Bradley, C.J.; Lin, C. Organizational Characteristics and Cancer Care for Nursing Home Residents. Health Serv Res **2009**, *44*, 1983–2003, doi:10.1111/j.1475-6773.2009.01024.x.
10. Dasch, B.; Blum, K.; Gude, P.; Bausewein, C. Place of Death: Trends Over the Course of a Decade: A Population-Based Study of Death Certificates From the Years 2001 and 2011. Dtsch Arztebl Int **2015**, *112*, 496–504, doi:10.3238/arztebl.2015.0496.
11. De Gagne, J.C.; Park, H.K.; Hall, K.; Woodward, A.; Yamane, S.; Kim, S.S. Microlearning in Health Professions Education: Scoping Review. JMIR Med Educ 2019, 5, e13997, doi:10.2196/13997.
12. Drageset, J.; Corbett, A.; Selbaek, G.; Husebo, B.S. Cancer-Related Pain and Symptoms among Nursing Home Residents: A Systematic Review. J Pain Symptom Manage **2014**, *48*, 699-710.e1, doi:10.1016/j.jpainsymman.2013.12.238.
13. Drageset, J.; Eide, G.E.; Dysvik, E.; Furnes, B.; Hauge, S. Loneliness, Loss, and Social Support among Cognitively Intact Older People with Cancer, Living in Nursing Homes--a Mixed-Methods Study. Clin Interv Aging **2015**, *10*, 1529–1536, doi:10.2147/CIA.S88404.
14. Dubé, C.E.; Mack, D.S.; Hunnicutt, J.N.; Lapane, K.L. Cognitive Impairment and Pain Among Nursing Home Residents With Cancer. J Pain Symptom Manage **2018**, *55*, 1509–1518, doi:10.1016/j.jpainsymman.2018.02.012.

15. Dyason, C.; Simon, J.; Wityk Martin, T.L. Advance Care Planning Discussions among Residents of Long Term Care and Designated Assisted Living: Experience from Calgary, Alberta. BMJ Support Palliat Care **2015**, *5*, 48–53, doi:10.1136/bmjspcare-2013-000444.
16. Evans, C.J.; Ho, Y.; Daveson, B.A.; Hall, S.; Higginson, I.J.; Gao, W.; GUIDE_Care project Place and Cause of Death in Centenarians: A Population-Based Observational Study in England, 2001 to 2010. PLoS Med. 2014, 11, e1001653, doi:10.1371/journal.pmed.1001653.
17. Fennell, M.L. Nursing Homes and Cancer Care. Health Serv Res **2009**, *44*, 1927–1932, doi:10.1111/j.1475-6773.2009.01056.x.
18. Finlayson, E.; Zhao, S.; Boscardin, W.J.; Fries, B.E.; Landefeld, C.S.; Dudley, R.A. Functional Status after Colon Cancer Surgery in Elderly Nursing Home Residents. J Am Geriatr Soc **2012**, *60*, 967–973, doi:10.1111/j.1532-5415.2012.03915.x.
19. Ghosh, T.; Jandhyala, D.; Bhatti, M.M.; Pritt, B.S. Closing the Brief Case: Crusted Scabies in a Leukemic Patient Following a Stay in a Long-Term Acute Care Facility. J. Clin. Microbiol. **2017**, *55*, 1599–1600, doi:10.1128/JCM.02375-16.
20. Ghosh, T.; Jandhyala, D.; Bhatti, M.M.; Pritt, B.S. The Brief Case: Crusted Scabies in a Leukemic Patient Following a Stay in a Long-Term Acute Care Facility. J. Clin. Microbiol. **2017**, *55*, 1233–1236, doi:10.1128/JCM.02350-16.
21. Hamaker, M.E.; Hamelinck, V.C.; van Munster, B.C.; Bastiaannet, E.; Smorenburg, C.H.; Achterberg, W.P.; Liefers, G.-J.; de Rooij, S.E. Nonreferral of Nursing Home Patients with Suspected Breast Cancer. J Am Med Dir Assoc **2012**, *13*, 464–469, doi:10.1016/j.jamda.2012.01.002.
22. Hibbert, P.D.; Wiles, L.K.; Cameron, I.D.; Kitson, A.; Reed, R.L.; Georgiou, A.; Gray, L.; Westbrook, J.; Augustsson, H.; Molloy, C.J.; et al. CareTrack Aged: The Appropriateness of Care Delivered to Australians Living in Residential Aged Care Facilities: A Study Protocol. *BMJ Open* 2019, 9, e030988, doi:10.1136/bmjopen-2019-030988.
23. Hodgson, N.A.; Lehning, A.J. Palliative Care in Nursing Homes: A Comparison of High- and Low-Level Providers. International Journal of Palliative Nursing **2008**, *14*, 38–44, doi:10.12968/ijpn.2008.14.1.28152.
24. Hunnicutt, J.N.; Tjia, J.; Lapane, K.L. Hospice Use and Pain Management in Elderly Nursing Home Residents With Cancer. J Pain Symptom Manage **2017**, *53*, 561–570, doi:10.1016/j.jpainsymman.2016.10.369.
25. Jeong, B.-H.; Koh, W.-J.; Yoo, H.; Park, H.Y.; Suh, G.Y.; Chung, M.P.; Kwon, O.J.; Jeon, K. Risk Factors for Acquiring Potentially Drug-Resistant Pathogens in Immunocompetent Patients with Pneumonia Developed out of Hospital. Respiration **2014**, *88*, 190–198, doi:10.1159/000362673.
26. Jokanovic, N.; Kautiainen, H.; Bell, J.S.; Tan, E.C.K.; Pitkälä, K.H. Change in Prescribing for Secondary Prevention of Stroke and Coronary Heart Disease in Finnish Nursing Homes and Assisted Living Facilities. Drugs & Aging **2019**, *36*, 571–579, doi:10.1007/s40266-019-00656-x.
27. Kanapuru, B.; Posani, K.; Muller, D.; Ershler, W.B. Decreased Cancer Prevalence in the Nursing Home. J Am Geriatr Soc **2008**, *56*, 2165–2166, doi:10.1111/j.1532-5415.2008.01864.x.
28. Kim, H.S.; Hwang, S.Y.; Oh, E.G.; Lee, J.E. Development and Evaluation of a PBL-based Continuing Education for Clinical Nurses: A Pilot Study. J Korean Acad Nurs **2006**, *36*, 1308, doi:10.4040/jkan.2006.36.8.1308.
29. Kiran, T.; Kopp, A.; Glazier, R.H. Those Left Behind From Voluntary Medical Home Reforms in Ontario, Canada. Ann Fam Med **2016**, *14*, 517–525, doi:10.1370/afm.2000.
30. Koblizek, V.; Novotna, B.; Zbozinkova, Z.; Hejduk, K. Diagnosing COPD: Advances in Training and Practice – a Systematic Review. AMEP 2016, 219, doi:10.2147/AMEP.S76976.
31. Koroukian, S.M.; Xu, F.; Beaird, H.; Diaz, M.; Murray, P.; Rose, J.H. Complexity of Care Needs and Unstaged Cancer in Elders: A Population-Based Study. Cancer Detect Prev **2007**, *31*, 199–206, doi:10.1016/j.cdp.2007.04.002.
32. LeBlanc, T.W.; Abernethy, A.P.; Casarett, D.J. What Is Different about Patients with Hematologic Malignancies? A Retrospective Cohort Study of Cancer Patients Referred to a Hospice Research Network. J Pain Symptom Manage **2015**, *49*, 505–512, doi:10.1016/j.jpainsymman.2014.07.003.
33. Lewis, M.A.; Kane, R.L.; Cretin, S.; Clark, V. The Immediate and Subsequent Outcomes of Nursing Home Care. Am J Public Health **1985**, *75*, 758–762, doi:10.2105/ajph.75.7.758.
34. Mack, D.S.; Epstein, M.M.; Dubé, C.; Clark, R.E.; Lapane, K.L. Screening Mammography among Nursing Home Residents in the United States: Current Guidelines and Practice. J Geriatr Oncol **2018**, *9*, 626–634, doi:10.1016/j.jgo.2018.05.005.
35. MacKenzie, M.A.; Buck, H.G.; Meghani, S.H.; Riegel, B. Unique Correlates of Heart Failure and Cancer Caregiver Satisfaction With Hospice Care. J Pain Symptom Manage **2016**, *51*, 71–78, doi:10.1016/j.jpainsymman.2015.09.001.
36. Matsumoto, S.; Harada, H.; Tanaka, J.; Ochi, K.; Ishibashi, T.; Takeda, M.; Yoshida, M.; Miyake, H.; Kimura, I. [Aging and exocrine pancreatic function]. Nihon Ronen Igakkai Zasshi **1989**, *26*, 146–152, doi:10.3143/geriatrics.26.146.
37. Meier, D.E.; Back, A.L.; Berman, A.; Block, S.D.; Corrigan, J.M.; Morrison, R.S. A National Strategy For Palliative Care. Health Affairs **2017**, *36*, 1265–1273, doi:10.1377/hlthaff.2017.0164.
38. Moinpour, C.M.; Polissar, L. Factors Affecting Place of Death of Hospice and Non-Hospice Cancer Patients. Am J Public Health **1989**, *79*, 1549–1551, doi:10.2105/ajph.79.11.1549.
39. Monroe, T.B.; Carter, M.A.; Feldt, K.S.; Dietrich, M.S.; Cowan, R.L. Pain and Hospice Care in Nursing Home Residents with Dementia and Terminal Cancer. Geriatr Gerontol Int **2013**, *13*, 1018–1025, doi:10.1111/ggi.12049.
40. Monroe, T.; Carter, M.; Feldt, K.; Tolley, B.; Cowan, R.L. Assessing Advanced Cancer Pain in Older Adults with Dementia at the End-of-Life. J Adv Nurs **2012**, *68*, 2070–2078, doi:10.1111/j.1365-2648.2011.05929.x.
41. Moore, K.L.; Lewis, C.L.; Zimmerman, S.; Ward, K.; Reed, D.; Rodriguez, R.; Kistler, C.E. A Pilot Study to Examine Colorectal Cancer Screening in Two Assisted Living Communities. J Am Geriatr Soc **2014**, *62*, 386–388, doi:10.1111/jgs.12665.

42. Murakami, M.; Ono, K.; Tsubokura, M.; Nomura, S.; Oikawa, T.; Oka, T.; Kami, M.; Oki, T. Was the Risk from Nursing-Home Evacuation after the Fukushima Accident Higher than the Radiation Risk? *PLoS ONE* 2015, 10, e0137906, doi:10.1371/journal.pone.0137906.
43. Murray, K.S.; Prunty, M.; Henderson, A.; Haden, T.; Pokala, N.; Ge, B.; Wakefield, M.; Petroski, G.F.; Mehr, D.R.; Kruse, R.L. Functional Status in Patients Requiring Nursing Home Stay After Radical Cystectomy. Urology **2018**, *121*, 39–43, doi:10.1016/j.urology.2018.07.030.
44. Murthy, S.B.; Shastri, A.; Merkler, A.E.; Hanley, D.F.; Ziai, W.C.; Fink, M.E.; Iadecola, C.; Kamel, H.; Navi, B.B. Intracerebral Hemorrhage Outcomes in Patients with Systemic Cancer. J Stroke Cerebrovasc Dis **2016**, *25*, 2918–2924, doi:10.1016/j.jstrokecerebrovasdis.2016.08.006.
45. Muss, H.B. Adjuvant Chemotherapy in Older and Younger Women With Lymph Node–Positive Breast Cancer. JAMA **2005**, *293*, 1073, doi:10.1001/jama.293.9.1073.
46. Paredes, A.Z.; Hyer, J.M.; Beal, E.W.; Bagante, F.; Merath, K.; Mehta, R.; White, S.; Pawlik, T.M. Impact of Skilled Nursing Facility Quality on Postoperative Outcomes after Pancreatic Surgery. Surgery **2019**, *166*, 1–7, doi:10.1016/j.surg.2018.12.008.
47. Pimentel, C.B.; Briesacher, B.A.; Gurwitz, J.H.; Rosen, A.B.; Pimentel, M.T.; Lapane, K.L. Pain Management in Nursing Home Residents with Cancer. J Am Geriatr Soc **2015**, *63*, 633–641, doi:10.1111/jgs.13345.
48. Porock, D.; Oliver, D.P.; Zweig, S.; Rantz, M.; Mehr, D.; Madsen, R.; Petroski, G. Predicting Death in the Nursing Home: Development and Validation of the 6-Month Minimum Data Set Mortality Risk Index. J. Gerontol. A Biol. Sci. Med. Sci. **2005**, *60*, 491–498, doi:10.1093/gerona/60.4.491.
49. Qanbari Qalehsari, M.; Khaghanizadeh, M.; Ebadi, A. Lifelong Learning Strategies in Nursing: A Systematic Review. Electron Physician **2017**, *9*, 5541–5550, doi:10.19082/5541.
50. Roxby, A.C.; Greninger, A.L.; Hatfield, K.M.; Lynch, J.B.; Dellit, T.H.; James, A.; Taylor, J.; Page, L.C.; Kimball, A.; Arons, M.; et al. Outbreak Investigation of COVID-19 Among Residents and Staff of an Independent and Assisted Living Community for Older Adults in Seattle, Washington. JAMA Intern Med **2020**, *180*, 1101–1105, doi:10.1001/jamainternmed.2020.2233.
51. Scholtes, C.; Baust, K.; Weinhold, L.; Creutzig, U.; Gnekow, A.; Hinz, A.; Kaatsch, P.; Kreitz, K.; Langer, T.; Rutkowski, S.; et al. Health Status, Health-Related Quality of Life, and Socioeconomic Outcome in Childhood Brain Tumor Survivors: A German Cohort Study. Neuro Oncol **2019**, *21*, 1069–1081, doi:10.1093/neuonc/noz044.
52. Shah, B.C.; Ullrich, F.; Smith, L.; Leiphrakpam, P.; Ly, Q.; Sasson, A.; Are, C. National Trends in Discharge Disposition after Hepatic Resection for Malignancy. HPB (Oxford) **2011**, *13*, 96–102, doi:10.1111/j.1477-2574.2010.00251.x.
53. Shugarman, L.R.; Bird, C.E.; Schuster, C.R.; Lynn, J. Age and Gender Differences in Medicare Expenditures and Service Utilization at the End of Life for Lung Cancer Decedents. Womens Health Issues **2008**, *18*, 199–209, doi:10.1016/j.whi.2008.02.008.
54. Silver, S.A.; Harel, Z.; McArthur, E.; Nash, D.M.; Acedillo, R.; Kitchlu, A.; Garg, A.X.; Chertow, G.M.; Bell, C.M.; Wald, R. Causes of Death after a Hospitalization with AKI. J Am Soc Nephrol **2018**, *29*, 1001–1010, doi:10.1681/ASN.2017080882.
55. Smith, A.K.; Lo, B.; Sudore, R. When Previously Expressed Wishes Conflict with Best Interests. JAMA Intern Med **2013**, *173*, 1241–1245, doi:10.1001/jamainternmed.2013.6053.
56. Sommerbakk, R.; Haugen, D.F.; Tjora, A.; Kaasa, S.; Hjermstad, M.J. Barriers to and Facilitators for Implementing Quality Improvements in Palliative Care - Results from a Qualitative Interview Study in Norway. BMC Palliat Care **2016**, *15*, 61, doi:10.1186/s12904-016-0132-5.
57. Tang, V.; Zhao, S.; Boscardin, J.; Sudore, R.; Covinsky, K.; Walter, L.C.; Esserman, L.; Mukhtar, R.; Finlayson, E. Functional Status and Survival After Breast Cancer Surgery in Nursing Home Residents. JAMA Surg **2018**, *153*, 1090–1096, doi:10.1001/jamasurg.2018.2736.
58. Temkin-Greener, H.; Zheng, N.T.; Xing, J.; Mukamel, D.B. Site of Death among Nursing Home Residents in the United States: Changing Patterns, 2003-2007. J Am Med Dir Assoc **2013**, *14*, 741–748, doi:10.1016/j.jamda.2013.03.009.
59. Thomas, K.S.; Boyd, E.; Mariotto, A.B.; Penn, D.C.; Barrett, M.J.; Warren, J.L. New Opportunities for Cancer Health Services Research: Linking the SEER-Medicare Data to the Nursing Home Minimum Data Set. Med Care 2018, 56, e90–e96, doi:10.1097/MLR.0000000000000877.
60. Tschirhart, E.C.; Du, Q.; Kelley, A.S. Factors Influencing the Use of Intensive Procedures at the End of Life. J Am Geriatr Soc **2014**, *62*, 2088–2094, doi:10.1111/jgs.13104.
61. Tuberculosis and Chest Service, Centre for Health Protection, Department of Health, Hong Kong; Leung, E.C.; Leung, C.; Chang, K.; Chan, C.; Mok, T.Y.; Chan, K.; Lau, K.; Chau, C.; Yee, W.K.; et al. Delayed Diagnosis of Tuberculosis: Risk Factors and Effect on Mortality among Older Adults in Hong Kong. *Hong Kong Med J* 2018, doi:10.12809/hkmj177081.
62. Unroe, K.T.; Sachs, G.A.; Dennis, M.E.; Hickman, S.E.; Stump, T.E.; Tu, W.; Callahan, C.M. Effect of Hospice Use on Costs of Care for Long-Stay Nursing Home Decedents. J Am Geriatr Soc **2016**, *64*, 723–730, doi:10.1111/jgs.14070.
63. Van Cleave, J.H.; Egleston, B.L.; Abbott, K.M.; Hirschman, K.B.; Rao, A.; Naylor, M.D. Multiple Chronic Conditions and Hospitalizations Among Recipients of Long-Term Services and Supports: Nursing Research **2016**, *65*, 425–434, doi:10.1097/NNR.0000000000000185.
64. Van Rensbergen, G.; Nawrot, T.S.; Van Hecke, E.; Nemery, B. Where Do the Elderly Die? The Impact of Nursing Home Utilisation on the Place of Death. Observations from a Mortality Cohort Study in Flanders. BMC Public Health **2006**, *6*, 178, doi:10.1186/1471-2458-6-178.
65. Walter, L.C.; Eng, C.; Covinsky, K.E. Screening Mammography for Frail Older Women: What Are the Burdens? J Gen Intern Med **2001**, *16*, 779–784, doi:10.1111/j.1525-1497.2001.10113.x.

66. Whitley, R.J.; Monto, A.S. Prevention and Treatment of Influenza in High-Risk Groups: Children, Pregnant Women, Immunocompromised Hosts, and Nursing Home Residents. J. Infect. Dis. 2006, 194 Suppl 2, S133-138, doi:10.1086/507548.
67. Zueger, P.M.; Holmes, H.M.; Calip, G.S.; Qato, D.M.; Pickard, A.S.; Lee, T.A. Older Medicare Beneficiaries Frequently Continue Medications with Limited Benefit Following Hospice Admission. J Gen Intern Med **2019**, *34*, 2029–2037, doi:10.1007/s11606-019-05152-x.
68. Aangenendt-Siegers, I.P.; Cools, H.J. [Cause of death in nursing homes]. Ned Tijdschr Geneeskd **1992**, *136*, 2015–2017.
69. Aberger, K.; Trtchounian, A.; DiPasquale, I.; Nelson, J.W. Early Palliative Care Consultation Decreases Lenth of Stay. J. Palliative Med. 2018, 21, A31, doi:10.1089/jpm.2018.29007.abstract.
70. Acher, A.W.; Squires, M.H.; Fields, R.C.; Poultsides, G.A.; Schmidt, C.; Votanopoulos, K.I.; Pawlik, T.M.; Jin, L.X.; Ejaz, A.; Kooby, D.A.; et al. Can the Risk of Non-Home Discharge after Resection of Gastric Adenocarcinoma Be Predicted: A Seven-Institution Study of the US Gastric Cancer Collaborative. *J. Gastrointest. Surg.* **2015**, *19*, 207–216, doi:10.1007/s11605-014-2690-2.
71. Ahonen, A.; Penttilä, A. Mortality and Autopsy Rate for Gastrointestinal Diseases in Finland in 1955--1973. Scand J Gastroenterol **1979**, *14*, 721–728, doi:10.3109/00365527909181944.
72. Akindele, A.; Lazar, P. Hypercalcemia of Undetermined Origin or Possibly Multifactorial. J. Am. Geriatr. Soc. 2017, 65, S193, doi:10.1111/imj.13426.
73. Alexander, K.; Shahrokni, A.; Mahmoudzadeh Pournaki, S.; Korc-Grodzicki, B. Skilled Care Utilization after Abdominal Cancer Surgery in Older Patients. J. Am. Geriatr. Soc. 2015, 63, S89, doi:10.1111/jgs.13439.
74. Alhelih, E.M.; Baker, O.G.; Almomani, S.A.; Baker, B.G. CANCER TREATMENT - RELATED SyMPTOMS AGGREGATION AND DISTRIBUTION AMONG SAUDI CHILDREN WITH CANCER. Middle East Journal of Nursing **2018**, *12*, 14–31, doi:10.5742/MEJN.2018.93393.
75. Alikhan, M.A.; Limesand, D.L.; Schmelzel, M.; McGlone, T.; Powers, D.; Khawaja, O.; Johnson, R.F.; Pikul, F. Management of Octogenarians with Female Breast Cancer in a Rural Oncology Program. J. Clin. Oncol. 2012, 30.
76. Aliyu, S.; Cohen, B.; Liu, J.; Larson, E. Prevalence and Risk Factors for Bloodstream Infection Present on Hospital Admission. Journal of Infection Prevention **2018**, *19*, 37–42, doi:10.1177/1757177417720998.
77. Altieri, C. Success of a Home-Based Oncology Rehabilitation Program for a Geriatric Patient Living in an Assisted Living Facility: A Case Report. Rehabilitation Oncology **2012**, *30*, 8–12.
78. Aly, A.; Singh, P.; Korytowsky, B.; Ling, Y.-L.; Kale, H.P.; Dastani, H.B.; Botteman, M.F.; Norden, A.D. Survival, Costs, and Health Care Resource Use by Line of Therapy in US Medicare Patients with Newly Diagnosed Glioblastoma: A Retrospective Observational Study. Neuro-Oncol. Pract. **2020**, *7*, 164–175, doi:10.1093/nop/npz042.
79. Andershed, B.; Ternestedt, B.M. Involvement of Relatives in Care of the Dying in Different Care Cultures: Development of a Theoretical Understanding. Nurs Sci Q **1999**, *12*, 45–51, doi:10.1177/08943189922106404.
80. Ankrett VO; Williams I Basal Cell Carcinoma. Update **2004**, *68*, 456–456.
81. Arao, Y.; Sasabe, M.; Yamada, Y.; Motoyama, Y.; Morita, K.; Kawazoe, M.; Ono, Y. [A consideration of the support for terminal care at the private skilled nursing home]. Gan To Kagaku Ryoho 2009, 36 Suppl 1, 42–44.
82. Argote, J.A. Patient with Myeloma Who Develops Thrombotic Microangiopathy without Hemolysis. Blood 2018, 132, doi:10.1182/blood-2018-99-117095.
83. Aslam, N.; Ali, M.U.; Rai, M. Candida Lambica; an Exceptional and Rare Fungal Infection. J. Gen. Intern. Med. 2020, 35, S419, doi:10.1007/s11606-020-05890-3.
84. Astuti, T.; Rochani, N.S.; Amrihati, E.T.; Rochimiwati, S.N.; Bustos, A.R. Snack-Based Pury, Safe and Beneficial to Maintain the Lipid Profile in Elderly. Ann. Trop. Med. Public Health **2020**, *23*, 1301–1308, doi:10.36295/ASRO.2020.23817.
85. Au, T.-Y. Perceived Stress, Spirituality, Resourcefulness and Sexuality in Patients with Rectal Cancer Undergoing Cancer Treatment., Case Western Reserve University, 2009.
86. Axelsson, B.; Christensen, S.B. Place of Death Correlated to Sociodemographic Factors. A Study of 203 Patients Dying of Cancer in a Rural Swedish County in 1990. Palliat Med **1996**, *10*, 329–335, doi:10.1177/026921639601000409.
87. Aydoğdu, N.; Çapık, C.; Ersin, F.; Kissal, A.; Bahar, Z. The Reliability and Validity of Prostate Cancer Fatalism Inventory in Turkish Language. Journal of Religion & Health **2017**, *56*, 1670–1682, doi:10.1007/s10943-016-0302-z.
88. Baali, N. Dermatology in Retirement Homes in Morocco. J. Dermatol. Nurses' Assoc. 2020, 12.
89. Bach, L.E.; Mortimer, J.A.; VandeWeerd, C.; Corvin, J. The Association of Physical and Mental Health with Sexual Activity in Older Adults in a Retirement Community. J Sex Med **2013**, *10*, 2671–2678, doi:10.1111/jsm.12308.
90. Badgwell, B.; Stanley, J.; Chang, G.J.; Katz, M.H.G.; Lin, H.Y.; Ning, J.; Klimberg, S.V.; Cormier, J.N. Comprehensive Geriatric Assessment of Risk Factors Associated with Adverse Outcomes and Resource Utilization in Cancer Patients Undergoing Abdominal Surgery. J Surg Oncol **2013**, *108*, 182–186, doi:10.1002/jso.23369.
91. Bagheri, I.; Shan, Y.; Klaassen, Z.; Kamat, A.M.; Konety, B.; Mehta, H.B.; Baillargeon, J.G.; Srinivas, S.; Tyler, D.S.; Swanson, T.A.; et al. Comparing Costs of Radical Versus Partial Cystectomy for Patients Diagnosed with Localized Muscle-Invasive Bladder Cancer: Understanding the Value of Surgical Care. *Urol. Oncol. Semin. Orig. Invest.* **2020**, *38*, 903, doi:10.1016/j.urolonc.2020.10.044.
92. Bahl, A.; Hoefeler, H.; Duran, I.; Hechmati, G.; Garzon-Rodriguez, C.; Ashcroft, J.; Lorusso, V.; Ghelani, P.; Wei, R.; Thomas, E.; et al. Health Resource Utilization Associated with Skeletal-Related Events in Patients with Advanced Prostate Cancer: A European Subgroup Analysis from an Observational, Multinational Study. J. Clin. Med. **2014**, *3*, 883–896, doi:10.3390/jcm3030883.

93. Bai, Q.; Zhang, Z.; Lu, X.; Shi, Y.; Liu, X.; Chan, H. Attitudes towards Palliative Care among Patients and Health Professionals in Henan, China. Prog. Palliative Care **2010**, *18*, 341–345, doi:10.1179/1743291X10Y.0000000006.
94. Bainbridge, D.; Seow, H.; Sussman, J.; Pond, G. Factors Associated with Acute Care Use among Nursing Home Residents Dying of Cancer: A Population-Based Study. Int J Palliat Nurs **2015**, *21*, 349–356, doi:10.12968/ijpn.2015.21.7.349.
95. Bakker, D.; Butler, L.; Fitch, M.; Green, E.; Olson, K.; Cummings, G. Canadian Cancer Nurses' Views on Recruitment and Retention. J Nurs Manag **2010**, *18*, 205–214, doi:10.1111/j.1365-2834.2009.01029.x.
96. Baldwin, K.M.; Katz, S.C.; Suarez, L.; Somasundar, P. Laparoscopic Colon Cancer Surgery in the Elderly: Results Are Similar to Those of Younger Patients. Ann. Surg. Oncol. 2012, 19, S108, doi:10.1245/s10434-012-2244-3.
97. Barber-Madden, R.; Glanz, K. Participatory Learning in Continuing Nursing Education: Soviet Edition of a Public Health and Medicine Trivia Game. J Contin Educ Nurs **1989**, *20*, 128–131.
98. Barchi, F.; Kasimatis Singleton, M.; Magama, M.; Shaibu, S. Building Locally Relevant Ethics Curricula for Nursing Education in Botswana. International Nursing Review **2014**, *61*, 491–498, doi:10.1111/inr.12138.
99. Barckley, V. Cancer Consultant to Nursing Homes. Am J Nurs **1970**, *70*, 804–806.
100. Bashari, D.R.; Iofel, Y. Ceftriaxone-Induced Immune Hemolytic Anemia in a Nonagenarian Identified at a Skilled Nursing Facility. Ann. Long-Term Care **2017**, *25*, 33–37, doi:10.25270/altc.2017.10.00011.
101. Bass, E.; French, D.D.; Bradham, D.D. A National Perspective of Medicare Expenditures for Elderly Veterans with Hip Fractures. J Am Med Dir Assoc **2008**, *9*, 114–119, doi:10.1016/j.jamda.2007.10.001.
102. Bassett, S.D.; Smyer, T. Health Screening Practices in Rural Long-Term Care Facilities. J Gerontol Nurs **2003**, *29*, 42–49, doi:10.3928/0098-9134-20030401-10.
103. Bastiaannet, E.; Hamaker, M.E.; Hamelinck, V.C.; Van Munster, B.C.; Achterberg, W.P.; Liefers, G.J.; Smorenburg, C.H.; De Rooij, S.E. Referral Patterns for Older Patients with Suspected Breast Cancer in Nursing Homes. Eur. J. Cancer 2012, 48, S66.
104. Bates, B.E.; Kwong, P.L.; Xie, D.; Valimahomed, A.; Ripley, D.C.; Kurichi, J.E.; Stineman, M.G. Factors Influencing Receipt of Early Rehabilitation after Stroke. Arch Phys Med Rehabil **2013**, *94*, 2349–2356, doi:10.1016/j.apmr.2013.07.021.
105. Bayer, V.; Amaya, B.; Baniewicz, D.; Callahan, C.; Marsh, L.; McCoy, A.S. Cancer Immunotherapy: An Evidence-Based Overview and Implications for Practice. Clin J Oncol Nurs **2017**, *21*, 13–21, doi:10.1188/17.CJON.S2.13-21.
106. Bensted, K.; Vadali, N.; Freiman, J. Advanced Care Planning and Impact of Palliative Care on Patients with Cirrhosis and Hepatocellular Carcinoma. J. Gastroenterol. Hepatol. **2018**, *33*, 35, doi:10.1111/jgh.14393.
107. Berger-Höger, B.; Liethmann, K.; Mühlhauser, I.; Steckelberg, A. Implementation of Shared Decision-Making in Oncology: Development and Pilot Study of a Nurse-Led Decision-Coaching Programme for Women with Ductal Carcinoma in Situ. BMC Med Inform Decis Mak **2017**, *17*, 160, doi:10.1186/s12911-017-0548-8.
108. Bergevin, R.C. Old Willie. J Gerontol Nurs **2005**, *31*, 60, doi:10.3928/0098-9134-20050201-12.
109. Bern-Klug, M.; Singh, J.; Liu, J.; Shinkunas, L. Prospect Theory Concepts Applied to Family Members of Nursing Home Residents with Cancer: A Good Ending Is a Gain. J Soc Work End Life Palliat Care **2019**, *15*, 34–54, doi:10.1080/15524256.2019.1580242.
110. Berry, P.H. The Pain of Residents with Terminal Cancer in USA Nursing Homes: Family Members' Perspectives. Int J Palliat Nurs **2007**, *13*, 20–27, doi:10.12968/ijpn.2007.13.1.22777.
111. Berwick, M.; Oliveria, S.; Luo, S.-T.; Headley, A.; Bolognia, J.L. A Pilot Study Using Nurse Education as an Intervention to Increase Skin Self-Examination for Melanoma. J. Cancer Educ. **2000**, *15*, 38–40.
112. Bhagat, R.; Bronsert, M.R.; Henderson, W.G.; Scott, C.D.; Weyant, M.J.; Mitchell, J.D.; Fullerton, D.A.; Meguid, R.A. Analysis of Discharge Destination After Open Versus Minimally Invasive Surgery for Lung Cancer. Ann Thorac Surg **2020**, *109*, 375–382, doi:10.1016/j.athoracsur.2019.08.059.
113. Billmeier, S.E.; Ayanian, J.Z.; He, Y.; Jaklitsch, M.T.; Rogers, S.O. Predictors of Nursing Home Admission, Severe Functional Impairment, or Death One Year after Surgery for Non-Small Cell Lung Cancer. Ann. Surg. **2013**, *257*, 555–563, doi:10.1097/SLA.0b013e31828353af.
114. Blanař, V.; Hödl, M.; Lohrmann, C.; Amir, Y.; Eglseer, D. Dysphagia and Factors Associated with Malnutrition Risk: A 5-year Multicentre Study. Journal of Advanced Nursing (John Wiley & Sons, Inc.) **2019**, *75*, 3566–3576, doi:10.1111/jan.14188.
115. Bliss, M.; Jordan, P. Long-Term Care a Priority Issue for Patients with Cancer. ONS News **1993**, *8*, 6.
116. Boland, A.; Haycox, A.; Bagust, A.; Fitzsimmons, L. A Randomised Controlled Trial to Evaluate the Clinical and Cost-Effectiveness of Hickman Line Insertions in Adult Cancer Patients by Nurses. Health Technol. Assess. 2003, 7, doi:10.3310/hta7360.
117. Boorsma, M.; Joling, K.; Dussel, M.; Ribbe, M.; Frijters, D.; van Marwijk, H.W.J.; Nijpels, G.; van Hout, H. The Incidence of Depression and Its Risk Factors in Dutch Nursing Homes and Residential Care Homes. The American Journal of Geriatric Psychiatry **2012**, *20*, 932–942, doi:10.1097/JGP.0b013e31825d08ac.
118. Bosworth, H.B.; Bastian, L.A.; Siegler, I.C. Benefits and Drawbacks to Hormone Replacement Therapy among Nursing Home Patients. Womens Health Issues **1998**, *8*, 53–59, doi:10.1016/s1049-3867(97)00094-7.
119. Bouëtté, G.; Esvan, M.; Apel, K.; Thibault, R. THE SELF-EVALUATION OF FOOD INTAKE (SEFI®) AS A SCREENING TEST FOR MALNUTRITION IN GENERAL PRACTICE: PROSPECTIVE NON-INTERVENTIONAL MULTICENTER STUDY. Clin. Nutr. 2019, 38, S21, doi:10.1016/S0261-5614(19)32516-6.
120. Bourbonniere, M.; Van Cleave, J.H. Cancer Care in Nursing Homes. Semin Oncol Nurs **2006**, *22*, 51–57, doi:10.1016/j.soncn.2005.10.007.

121. Bradley, J.P. A Descriptive Survey of Skin Cancer in a Geriatric Centre 1988-1989. Aust Fam Physician **1991**, *20*, 1461, 1464, 1467–1468.
122. Brechtelsbauer D Lessons Learned from an Old Acquaintance. Caring for the Ages **2006**, *7*, 6–7, doi:10.1016/s1526-4114(06)60319-3.
123. Breuer, B.; Anderson, R. The Relationship of Tamoxifen with Dementia, Depression, and Dependence in Activities of Daily Living in Elderly Nursing Home Residents. Women Health **2000**, *31*, 71–85, doi:10.1300/J013v31n01_05.
124. Breuer, B.; Subramaniam, G.; Marchello, V.; Giambanco, V. A Study of the Effects of Tamoxifen in the Frail Elderly: Difficulties Encountered in Nursing Home Research. Women Health **1997**, *25*, 37–51, doi:10.1300/J013v25n01_03.
125. Buchanan, R.J.; Barkley, J.; Wang, S.; Kim, M. Analyses of Nursing Home Residents with Cancer at Admission. Cancer Nurs **2005**, *28*, 406–414, doi:10.1097/00002820-200509000-00012.
126. Burgess, C.; Teasdale, E.; Omar, L.; Tucker, L.; Ramirez, A.-J. Training Radiographers to Deliver an Intervention to Promote Early Presentation of Breast Cancer. Radiography **2012**, *18*, 232–237, doi:10.1016/j.radi.2012.06.003.
127. Burguete, D.; Mokdad, A.A.; Augustine, M.M.; Minter, R.; Mansour, J.C.; Choti, M.A.; Polanco, P.M. Non-Home Discharge and Prolonged Length of Stay After Cytoreductive Surgery and HIPEC. J Surg Res **2019**, *233*, 360–367, doi:10.1016/j.jss.2018.08.018.
128. Burns, B.C. Oral Care for Older People in Residential. Nursing & Residential Care **2012**, *14*, 26–31.
129. Cagle, J.G.; Lee, J.; Ornstein, K.A.; Guralnik, J.M. Hospice Utilization in the United States: A Prospective Cohort Study Comparing Cancer and Noncancer Deaths. Journal of the American Geriatrics Society **2020**, *68*, 783–793, doi:10.1111/jgs.16294.
130. Campbell, K. Leukaemia. Knowledge for Practice (Continuing Education Credit). Nurs Times 1997, 93, suppl 1-4.
131. Campbell, S.M.; Louie-Gao, Q.; Hession, M.L.; Bailey, E.; Geller, A.C.; Cummins, D. Skin Cancer Education among Massage Therapists: A Survey at the 2010 Meeting of the American Massage Therapy Association. Journal of Cancer Education **2013**, *28*, 158–164, doi:10.1007/s13187-012-0403-7.
132. Caranasos, G.J. Prevalence of Cancer in Older Persons Living at Home and in Institutions. Clin. Geriatr. Med. **1997**, *13*, 15–31.
133. Carethers, J.M. Potential Insights into COVID-19 Disparitiesfrom the Science of Cancer Health Disparities. Clin. Cancer Res. 2020, 26, doi:10.1158/1557-3265.COVID-19-IA20.
134. Carpenter Joan G; Berry Patricia H. Refractory Cancer Pain in a Nursing Home Resident. Journal of Hospice & Palliative Nursing **2012**, *14*, 516–523, doi:10.1097/njh.0b013e318273b983.
135. Caucat, M.; Zacarin, A.; Rousseau, V.; Montastruc, J.-L.; Bagheri, H. The Cost of Potentially Inappropriate Medications in Nursing Homes in West Occitanie. Pharm. 2020, 8, doi:10.3390/pharmacy8010039.
136. Cavalieri, T.A. Pain Management at the End of Life. Clin. Geriatr. **2005**, *13*, 44–52.
137. Cha, E.; Kim, J.; Sohn, M.K.; Lee, B.S.; Jung, S.S.; Lee, S.; Lee, I. Perceptions on Good-Life, Good-Death, and Advance Care Planning in Koreans with Non-Cancerous Chronic Diseases. J Adv Nurs **2021**, *77*, 889–898, doi:10.1111/jan.14633.
138. Chan-Yeung, M.; Cheung, A.H.K.; Dai, D.L.K.; Chan, F.H.W.; Kam, K.M.; Tam, C.M.; Leung, C.C. Prevalence and Determinants of Positive Tuberculin Reactions of Residents in Old Age Homes in Hong Kong. Int J Tuberc Lung Dis **2006**, *10*, 892–898.
139. Chang, B. A Rare Case of Lymphoma. J. Gen. Intern. Med. 2017, 32, S416.
140. Chang, Y.-L.; Tsai, Y.-F.; Hsu, C.-L.; Chao, Y.-K.; Hsu, C.-C.; Lin, K.-C. The Effectiveness of a Nurse-Led Exercise and Health Education Informatics Program on Exercise Capacity and Quality of Life among Cancer Survivors after Esophagectomy: A Randomized Controlled Trial. International Journal of Nursing Studies 2020, 101, N.PAG-N.PAG, doi:10.1016/j.ijnurstu.2019.103418.
141. Chauncey, H.H.; House, J.E. Dental Problems in the Elderly. Hosp Pract **1977**, *12*, 81–86, doi:10.1080/21548331.1977.11707244.
142. Chen, J.C.Y.; Thorsteinsdottir, B.; Williams, A.W.; Feely, M.; Hickson, L.T. Death and Hemodialysis Withdrawal in Incident Dialysis Patients. Hemodial. Int. 2017, 21, A13, doi:10.1111/hdi.12520.
143. Cheng, E.; Thorpe, E.; Borrowdale, R. Spinal Epidural Abscess Following Glossectomy and Neck Dissection: A Case Report. Int. J. Surg. Case Rep. **2016**, *20*, 33–36, doi:10.1016/j.ijscr.2016.01.004.
144. Cheng, H.Y.; Tonorezos, E.; Zorowitz, R.; Novotny, J.; Dubin, S.; Maurer, M.S. Inpatient Care for Nursing Home Patients: An Opportunity to Improve Transitional Care. J Am Med Dir Assoc **2006**, *7*, 383–387, doi:10.1016/j.jamda.2006.01.025.
145. Chesterman, T.; Zimmermann, A.; Mah, P.M.; Vora, P. Profound Hypocalcaemia Following Denosumab for Metastatic Prostate Carcinoma; An under-Recognised and Potentially Fatal Complication. Clin. Endocrinol. **2018**, *89*, 91–92.
146. Chini, C.; Nigro, O.; Giaquinto, A.; Magni, E.; Tinelli, G.; Bascialla, L.; Gobba, S.; Pinotti, G. Homcology: Home Chemotherapy Delivery in a Simultaneous Care Project for Frail Advanced Cancer Patients. Ann. Oncol. 2019, 30, v728–v729, doi:10.1093/annonc/mdz265.032.
147. Choi, N.G.; DiNitto, D.M.; Marti, C.N.; Conwell, Y. Physical Health Problems as a Late-Life Suicide Precipitant: Examination of Coroner/Medical Examiner and Law Enforcement Reports. Gerontologist **2019**, *59*, 356–367, doi:10.1093/geront/gnx143.
148. Cholankeril, G.; Hu, M.; Tanner, E.; Cholankeril, R.; Reha, J.; Somasundar, P. Skilled Nursing Facility Placement in Hospitalized Elderly Patients with Colon Cancer. Eur J Surg Oncol **2016**, *42*, 1660–1666, doi:10.1016/j.ejso.2016.06.005.
149. Christos PJ; Oliveria SA; Mâsse LC; McCormick LK; Halpern AC Skin Cancer Prevention and Detection by Nurses: Attitudes, Perceptions, and Barriers. Journal of Cancer Education **2004**, *19*, 50–57, doi:10.1207/s15430154jce1901_12.
150. Clark, M.; Julmisse, M.; Marcelin, N.; Merry, L.; Tuck, J.; Gagnon, A.J. Strengthening Healthcare Delivery in Haiti through Nursing Continuing Education. Int Nurs Rev **2015**, *62*, 54–63, doi:10.1111/inr.12165.
151. Coghill, A.S. Care Coordination Helps Older Adults with Cancer Continue to Live Life to the Fullest. ONS Connect **2014**, *29*, 24–28.

152. Cohen, A.C.; Davidson, N.; Scharf, J.; Middlebrook, D. Cost Burden Analysis of Ineffective Induction Chemotherapy in Elderly Patients with AML. Blood 2009, 114.
153. Coleman EA; Stewart CB; Wilson S; Cantrell MJ; O'Sullivan P; Carthron DO; Wood LC An Evaluation of Standardized Patients in Improving Clinical Breast Examinations for Military Women. Cancer Nursing **2004**, *27*, 474–482.
154. Coleman, J.; Levy, J.; Wiggins, S.; Kinley, J. Using a New End-of-Life Care Plan in Nursing Homes. Nursing & Residential Care **2017**, *19*, 38–41, doi:10.12968/nrec.2017.19.1.38.
155. Cooper, G.S.; Xu, F.; Koroukian, S.; Barnholtz-Sloan, J.; Schluchter, M. Colorectal Cancer Diagnosis without Colonoscopy: Frequency and Predictive Factors. Gastroenterology 2010, 138, S187.
156. Corica, T.; Joseph, D.; Saunders, C.; Bulsara, M.; Nowak, A.K. Intraoperative Radiotherapy for Early Breast Cancer: Do Health Professionals Choose Convenience or Risk? Radiat. Oncol. 2014, 9, doi:10.1186/1748-717X-9-33.
157. Cornwell, L.B.; Bratslavsky, G.; Ginzburg, N. Factors Associated with Length of Stay and Discharge Disposition for Elective Bladder Cancer Surgical Admissions: A SPARCS Analysis. Can. Urol. Assoc. J. 2018, 12, S221, doi:10.5489/cuaj.5656.
158. Corona, G.; Dinardo, F.; Bochicchio, G.B. Home Placement of PICCs and Midlines in Patients over Eighty Years Old. A Three-Year Experience. Nutritional Therapy & Metabolism **2011**, *29*, 139–144.
159. Corrao, S.; Santalucia, P.; Argano, C.; Djade, C.D.; Barone, E.; Tettamanti, M.; Pasina, L.; Franchi, C.; Kamal Eldin, T.; Marengoni, A.; et al. *Gender-Differences in Disease Distribution and Outcome in Hospitalized Elderly: Data from the REPOSI Study. Eur. J. Intern. Med.* **2014**, *25*, 617–623, doi:10.1016/j.ejim.2014.06.027.
160. Costello, J. Research Roundup. International Journal of Palliative Nursing **2017**, *23*, 100–101, doi:10.12968/ijpn.2017.23.2.100.
161. Custers, A.F.J.; Cillessen, A.H.J.; Westerhof, G.J.; Kuin, Y.; Riksen-Walraven, J.M. Need Fulfillment, Need Importance, and Depressive Symptoms of Residents over the First Eight Months of Living in a Nursing Home. Int. Psychogeriatr. **2014**, *26*, 1161–1170, doi:10.1017/S1041610214000659.
162. D'Alessandro, C.; Sandri, R.; Viti, N.; Ricciardi, T.; Cantatore, A.; Cossovich, P.; Rebecchi, I.; Galetti, G.; Monfardini, S. Cancer in an Italian Nursing Home. Ann. Oncol. 2010, 21, viii188, doi:10.1093/annonc/mdq520.
163. da Costa, C.C.; Freitas, L.V.; Dias, L.M.B.; Lima, T.M.; Damasceno, A.K. de C.; Pinheiro, A.K.B. Performance of Tests to Prevent Cervical Cancer: Promoting Health Nursing Home. Rev Rene **2010**, *11*, 27–35.
164. Dahlqvist, J.; Ekdahl, A.; Friedrichsen, M. Does Comprehensive Geriatric Assessment (CGA) in an Outpatient Care Setting Affect the Causes of Death and the Quality of Palliative Care? A Subanalysis of the Age-FIT Study. Eur. Geriatr. Med. **2019**, *10*, 455–462, doi:10.1007/s41999-019-00198-w.
165. Darjani, A.; Alizadeh, N.; Rafiei, E.; Moulaei, M.; Naseri Alavi, S.H.; Eftekhari, H.; Rafiei, R.; Gharaei-Nejad, K.; Mohtasham-Amiri, Z. Skin Diseases among the Old Age Residents in a Nursing Home: A Neglected Problem. Dermatology Research & Practice 2020, 1–5, doi:10.1155/2020/8849355.
166. Dasch, B.; Bausewein, C.; Feddersen, B. Place of Death in Patients with Dementia and the Association with Comorbidities: A Retrospective Population-Based Observational Study in Germany. BMC Palliative Care 2018, 17, N.PAG-N.PAG, doi:10.1186/s12904-018-0334-0.
167. De Gaetano, C.; Lichtman, S.M. Care of Elderly Women with Ovarian Cancer. Geriatr. Nurs. **2004**, *25*, 329–334, doi:10.1016/j.gerinurse.2004.09.015.
168. De La Motte Rouge, T.; Chaïbi, P.; Vignot, S.; Boussion, H.; Cabel, L.; Geiss, R.; Delgado, F.-M.; Khayat, D.; Chebib, A.; Spano, J.-P. Frequency of Cognitive Impairment (CI) in Elderly Patients (Pts) Suffering from Malignancies and Impact on Therapeutic Decision. J. Clin. Oncol. 2012, 30.
169. de Luis, D.A.; Izaola, O.; López, L.; Blanco, B.; Colato, C.A.; Kelly, O.J.; Sanz, R.; Bayo, H.; Olivares, M.; González, V.; et al. AdNut Study: Effectiveness of a High Calorie and Protein Oral Nutritional Supplement with β-Hydroxy-β-Methylbutyrate in an Older Malnourished Population in Usual Clinical Practice. *Eur. Geriatr. Med.* **2018**, *9*, 809–817, doi:10.1007/s41999-018-0109-4.
170. De Vito, A.; Fiore, V.; Princic, E.; Geremia, N.; Panu Napodano, C.M.; Muredda, A.A.; Maida, I.; Madeddu, G.; Babudieri, S. Predictors of Infection, Symptoms Development, and Mortality in People with SARS-CoV-2 Living in Retirement Nursing Homes. *PLoS ONE* 2021, 16, e0248009, doi:10.1371/journal.pone.0248009.
171. de Vries, M. Does the Education Tool Cancer in the Elderly Have International Potential?...Fourth European Nursing Congress. Journal of Clinical Nursing (John Wiley & Sons, Inc.) **2010**, *19*, 110–110, doi:10.1111/j.1365-2702.2010.03445.x.
172. Decker B; Hull AH Older Adults with Developmental Disabilities: Encouraging Participation. OT Practice 2005, 10, CE-2p.
173. Decker, S.L.; Higginson, I.J. A Tale of Two Cities: Factors Affecting Place of Cancer Death in London and New York. Eur J Public Health **2007**, *17*, 285–290, doi:10.1093/eurpub/ckl243.
174. Delarue, J.; Chomette, G.; Aryen, D. [Cancer in the elderly person. Necropsic enumeration in an old age home population. *]. Bull Assoc Fr Etud Cancer* **1961**, *48*, 18–34.
175. Devlin B; Agnew A An Evaluation of a Domiciliary Blood Transfusion Service for Palliative Care Patients in Northern Ireland. Community Practitioner **2008**, *81*, 32–35.
176. Diduszyn, J.; Boigon, M.; Glew, C.; Hofmann, M.T. Osteonecrosis of the Mandible in a Nursing Home Resident Receiving Bisphosphonate Therapy. Ann. Long-Term Care **2008**, *16*, 37–40.
177. Dixit, S.; Shumack, S. A Guide to Skin Conditions in Older People. Med. Today **2017**, *18*, 10–17.
178. Dobalian, A. Advance Care Planning Documents in Nursing Facilities: Results from a Nationally Representative Survey. Archives of Gerontology and Geriatrics **2006**, *43*, 193–212, doi:10.1016/j.archger.2005.10.007.

179. Dobalian, A.; Tsao, J.C.I.; Radcliff, T.A. Diagnosed Mental and Physical Health Conditions in the United States Nursing Home Population: Differences between Urban and Rural Facilities. J Rural Health **2003**, *19*, 477–483, doi:10.1111/j.1748-0361.2003.tb00585.x.
180. Dorney, K.M.; Growdon, W.B.; Clemmer, J.; Rauh-Hain, J.A.; Hall, T.R.; Diver, E.; Boruta, D.; Del Carmen, M.G.; Goodman, A.; Schorge, J.O.; et al. Patient, Treatment and Discharge Factors Associated with Hospital Readmission within 30days after Surgery for Vulvar Cancer. Gynecol. Oncol. **2017**, *144*, 136–139, doi:10.1016/j.ygyno.2016.11.009.
181. dos Santos, R.P.; de Almeida, A.C.C.H. THE PREVALENCE OF INTRA-EPITHELIAL LESIONS IN PREVENTIVE TESTS COLLECTED BY STUDENT NURSES: 2008-2012. Cogitare Enfermagem **2014**, *19*, 324–329.
182. Dou, Z.L. Prevalence of Dysphagia in China: An Epidemiology Survery among 6102 Participants. Neurorehabil. Neural Repair **2018**, *32*, 361, doi:10.1177/1545968318765497.
183. Downe-Wamboldt, B.L.; Butler, L.J.; Melanson, P.M.; Coulter, L.A.; Singleton, J.F.; Keefe, J.M.; Bell, D.G. The Effects and Expense of Augmenting Usual Cancer Clinic Care with Telephone Problem-Solving Counseling. Cancer Nurs. **2007**, *30*, 441–453, doi:10.1097/01.NCC.0000300164.90768.ec.
184. Drageset, J.; Eide, G.E.; Harrington, C.; Ranhoff, A.H. Acute Hospital Admission for Nursing Home Residents without Cognitive Impairment with a Diagnosis of Cancer. Eur J Cancer Care (Engl) **2015**, *24*, 147–154, doi:10.1111/ecc.12205.
185. Drageset, J.; Eide, G.E.; Kirkevold, M.; Ranhoff, A.H. Emotional Loneliness Is Associated with Mortality among Mentally Intact Nursing Home Residents with and without Cancer: A Five-Year Follow-up Study. J Clin Nurs **2013**, *22*, 106–114, doi:10.1111/j.1365-2702.2012.04209.x.
186. Drageset, J.; Eide, G.E.; Ranhoff, A.H. Cancer in Nursing Homes: Characteristics and Health-Related Quality of Life among Cognitively Intact Residents with and without Cancer. Cancer Nurs **2012**, *35*, 295–301, doi:10.1097/NCC.0b013e31822e7cb8.
187. Drageset, J.; Eide, G.E.; Ranhoff, A.H. Mortality in Nursing Home Residents without Cognitive Impairment and Its Relation to Self-Reported Health-Related Quality of Life, Sociodemographic Factors, Illness Variables and Cancer Diagnosis: A 5-Year Follow-up Study. Qual Life Res **2013**, *22*, 317–325, doi:10.1007/s11136-012-0143-5.
188. Drageset, J.; Eide, G.E.; Ranhoff, A.H. Anxiety and Depression and Mortality among Cognitively Intact Nursing Home Residents with and without a Cancer Diagnosis: A 5-Year Follow-up Study. Cancer Nurs 2013, 36, E68-74, doi:10.1097/NCC.0b013e31826fcb11.
189. Drolle, H.; Vehling-Kaiser, U.; Bäuml, E.; Flieser-Hartl, M.; Kaiser, F. Comparison: Proportion of Cancer Patients in the SAPV (Specialized Outpatient Palliative Care) and the Hospice with Comparison of the Length of Stay. Oncol. Res. Treat. **2016**, *39*, 178, doi:10.1159/000449050.
190. Duncan, J.G.; Bott, M.J.; Thompson, S.A.; Gajewski, B.J. Symptom Occurrence and Associated Clinical Factors in Nursing Home Residents with Cancer. Res Nurs Health **2009**, *32*, 453–464, doi:10.1002/nur.20331.
191. Duncan, J.G.; Forbes-Thompson, S.; Bott, M.J. Unmet Symptom Management Needs of Nursing Home Residents with Cancer. Cancer Nurs **2008**, *31*, 265–273, doi:10.1097/01.NCC.0000305738.33958.9f.
192. Eberle, C.M.; Winsemius, D.; Garibaldi, R.A. Risk Factors and Consequences of Bacteriuria in Non-Catheterized Nursing Home Residents. J Gerontol 1993, 48, M266-271, doi:10.1093/geronj/48.6.m266.
193. Ebner, H.; Spaengler, E. An investigation of the association between bullous pemphigoid and malignant disease. *WIEN. KLIN. WOCHENSCHR.* **1974**, *86*, 449–452.
194. Edwards, R.L.; Andan, C.; Lalla, R.V.; Lacouture, M.E.; O'Brien, D.; Sequist, L.V. Afatinib Therapy: Practical Management of Adverse Events With an Oral Agent for Non-Small Cell Lung Cancer Treatment. Clin J Oncol Nurs **2018**, *22*, 542–548, doi:10.1188/18.CJON.542-548.
195. Eichholz Heller, F.; Jimenez-Bautista, A. Building a Bridge to Connect Patients to Their Homeland at End of Life. Journal of Social Work in End-of-Life & Palliative Care **2015**, *11*, 96–100, doi:10.1080/15524256.2015.1074139.
196. Eisemon N; Stucky-Marshall L; Talamonti MS Screening for Colorectal Cancer: Developing a Preventive Healthcare Program Utilizing Nurse Endoscopists. Gastroenterology Nursing **2001**, *24*, 12–19.
197. Elbadawi, A.; Elgendy, I.Y.; Albaeni, A.; Aly, A.; Almustafa, A.; Shahin, H.I.; Chatterjee, A.; Alkhouli, M.; Khalife, W.; Kapadia, S.; et al. Trends and Outcomes of Transcatheter Valve Implantation in Patients With Prior Mediastinal Radiation. *Am J Cardiol* **2021**, *143*, 167–168, doi:10.1016/j.amjcard.2021.01.003.
198. Elder, A. The Digital Rectal Examination Still Matters. Diagnosis 2018, 5, eA107, doi:10.1515/dx-2018-0095.
199. Epstein, A.S.; Desai, A.V.; Bernal, C.; Romano, D.; Wan, P.J.; Okpako, M.; Anderson, K.; Chow, K.; Kramer, D.; Calderon, C.; et al. Giving Voice to Patient Values Throughout Cancer: A Novel Nurse-Led Intervention. *J. Pain Symptom Manage.* **2019**, *58*, 72-79.e2, doi:10.1016/j.jpainsymman.2019.04.028.
200. Epstein, J.B.; Lunn, R.; Le, N.D.; Stevenson-Moore, P.; Gorsky, M. Patients with Oropharyngeal Cancer: A Comparison of Adults Living Independently and Patients Living in Long-Term Care Facilities. Spec Care Dentist **2005**, *25*, 124–130, doi:10.1111/j.1754-4505.2005.tb01422.x.
201. Eroglu, M.Z.; Gunes, T.; Nebioglu, M. Suicide Attempt by Subcutaneous Injection of Cyanide: A Case Report. Dusunen Adam **2014**, *27*, 257–260, doi:10.5350/DAJPN2014270310.
202. Evans, K.D.; Bates, R.C. Describing Elderly Women in Assisted Living's Risk for Breast Cancer and Participation in Screening Behaviors. J. Diagn. Med. Sonogr. **2005**, *21*, 320–328, doi:10.1177/8756479305278354.
203. Falandry, C.; Brain, E.; Bonnefoy, M.; Mefti, F.; Savoye, A.; Rigal, O.; Oddou-Lagranière, S.; El Kouri, C.; Uwer, L.; Abadie-Lacourtoisie, S.; et al. Impact of Geriatric Vulnerability Parameters on Pegylated Liposomal Doxorubicin (PLD) Tolerance and

Outcome in Elderly Patients with Metastatic Breast Cancer: Results of the DOGMES Multicenter Phase II GINECO Trial. *J. Clin. Oncol.* 2011, 29.
204. Falandry, C.; Brain, E.; Bonnefoy, M.; Mefti, F.; Jovenin, N.; Rigal, O.; Guillem, O.; El Kouri, C.; Uwer, L.; Abadie-Lacourtoisie, S.; et al. Impact of Geriatric Risk Factors on Pegylated Liposomal Doxorubicin Tolerance and Efficacy in Elderly Metastatic Breast Cancer Patients: Final Results of the DOGMES Multicentre GINECO Trial. *Eur. J. Cancer* **2013**, *49*, 2806–2814, doi:10.1016/j.ejca.2013.04.027.
205. Feliciano, J.; Gardner, L.; Edelman, M.; Davidoff, A. First Survival Analysis in Elderly Patients (PTS) with Advanced Non-Small-Cell Lung Cancer (NSCLC) Stratified by a Predicted Disability Status (DS) Model: Analysis of Surveillance, Epidemiology and End Results (SEER)-Medicare Claims 2001-2005. J. Thorac. Oncol. 2012, 7, S210, doi:10.1097/JTO.0b013e318269fc07.
206. Fernández, P.; Fernández, E.; Arellano, M. Vaginal Moisturizing Post PDR-Pulse Dose Rate Brachytherapy. Ann. Oncol. 2019, 30, v849, doi:10.1093/annonc/mdz277.012.
207. Ferrell, B.A. Care of Cancer Patients in Nursing Homes. Oncology (Williston Park, N.Y.) **1992**, *6*, 141–145.
208. Ferrell, B.A.; Ferrell, B.R.; Osterweil, D. Pain in the Nursing Home. J Am Geriatr Soc **1990**, *38*, 409–414, doi:10.1111/j.1532-5415.1990.tb03538.x.
209. Ferrell, B.R.; Ferrell, B.A. Easing the Pain. Geriatr Nurs **1990**, *11*, 175–178, doi:10.1016/s0197-4572(05)80337-4.
210. Finlayson, E.; Zhao, S.; Varma, M.G. Outcomes after Rectal Cancer Surgery in Elderly Nursing Home Residents. Dis. Colon Rectum **2012**, *55*, 1229–1235, doi:10.1097/DCR.0b013e318267bfe3.
211. Firnhaber, C.; Mao, L.; Levin, S.; Faesen, M.; Lewis, D.A.; Goeieman, B.J.; Swarts, A.J.; Rakhombe, N.; Michelow, P.M.; Williams, S.; et al. Evaluation of a Cervicography-Based Program to Ensure Quality of Visual Inspection of the Cervix in HIV-Infected Women in Johannesburg, South Africa. J. Lower Genital Tract Dis. **2015**, *19*, 7–11, doi:10.1097/LGT.0000000000000040.
212. Flannery, M.; McAndrews, L.; Stein, K.F. Telephone Calls by Individuals With Cancer. Oncology Nursing Forum **2013**, *40*, 464–471, doi:10.1188/13.ONF.464-471.
213. Flynn, A.; Stewart, D.E. Where Do Cancer Patients Die? A Review of Cancer Deaths in Cuyahoga County, Ohio, 1957--1974. J Community Health **1979**, *5*, 126–130, doi:10.1007/BF01324014.
214. Forrest JL; Horowitz AM; Shmuely Y Dental Hygienists' Knowledge, Opinions, and Practices Related to Oral and Pharyngeal Cancer Risk Assessment. Journal of Dental Hygiene **2001**, *75*, 271–281.
215. Francke AL; Garssen B; Luiken JB; De Schepper AME; Grypdonck M; Abu-Saad HH Effects of a Nursing Pain Programme on Patient Outcomes. Psycho-Oncology **1997**, *6*, 302–310, doi:10.1002/(sici)1099-1611(199712)6:4<302::aid-pon284>3.0.co;2-d.
216. Frawley, H.C.; Lin, K.-Y.; Granger, C.L.; Higgins, R.; Butler, M.; Denehy, L. An Allied Health Rehabilitation Program for Patients Following Surgery for Abdomino-Pelvic Cancer: A Feasibility and Pilot Clinical Study. Supportive Care Cancer **2020**, *28*, 1335–1350, doi:10.1007/s00520-019-04931-w.
217. Freijer, K.; Tan, S.S.; Koopmanschap, M.A.; Meijers, J.M.M.; Halfens, R.J.G.; Nuijten, M.J.C. The Economic Costs of Disease Related Malnutrition. Clin Nutr **2013**, *32*, 136–141, doi:10.1016/j.clnu.2012.06.009.
218. Freund, K.M.; Reisinger, S.A.; LeClair, A.; Al-Najar, S.; Young, G.S.; Oliveri, J.; Gonzalez, E.; Paskett, E. Impact of Insurance and Primary Care Stability on Cancer Screening Behaviors. Cancer Epidemiol. Biomarkers Prev. 2018, 27, doi:10.1158/1538-7755.DISP17-C77.
219. Fukui, S.; Ogawa, K.; Yamagishi, A. Effectiveness of Communication Skills Training of Nurses on the Quality of Life and Satisfaction with Healthcare Professionals among Newly Diagnosed Cancer Patients: A Preliminary Study. Psycho-Oncology **2011**, *20*, 1285–1291, doi:10.1002/pon.1840.
220. Gage, B.; Dao, T. Medicare's Hospice Benefit: Use and Expenditures. J. Pharm. Care Pain Symptom Control **2001**, *9*, 57–75, doi:10.1300/J088v09n01_06.
221. Gerhart, J.I.; Sanchez Varela, V.; Burns, J.W. Brief Training on Patient Anger Increases Oncology Providers' Self-Efficacy in Communicating With Angry Patients. Journal of Pain & Symptom Management **2017**, *54*, 355-360.e2, doi:10.1016/j.jpainsymman.2017.07.039.
222. Giannini, I.; Baroncini, A.C.; Baroncini, C.; Latini, E.; Nucci, V.; Mossello, E.; Ungar, A.; Di Bari, M.; Cavallini, M.C. Delirium in Older Adults "'ergo'" Dementia, but If It Was a Brain Tumor? Eur. Geriatr. Med. 2018, 9, S315, doi:10.1007/s41999-018-0097-4.
223. Gimon, T.; MacLean, A.R. Ileal Mycobacterium Tuberculosis in a Patient Treated for Long-Standing Crohn's Disease. Dis. Colon Rectum 2018, 61, e122–e123, doi:10.1097/DCR.0000000000001104.
224. Gironi, R.; Fabbrini, M.; Clerici, D.; Oltolini, C.; Greco, R.; Corti, C.; Ciceri, F.; Biella, A. Effect of Nurses Education Program on Catheter Related Blood Stream Infections. Bone Marrow Transplant. **2020**, *55*, 731–732, doi:10.1038/s41409-020-01103-x.
225. Godino C; Jodar L; Durán Á; Martínez I; Schiaffino A Nursing Education as an Intervention to Decrease Fatigue Perception in Oncology Patients. European Journal of Oncology Nursing **2006**, *10*, 150–155, doi:10.1016/j.ejon.2005.03.004.
226. Goldberg, J.I.; Burhenn, P.S.; Ginex, P.K. Nursing Education: Review of Assessment, Clinical Care, and Implications for Practice Regarding Older Adult Patients with Cancer. Clinical Journal of Oncology Nursing **2018**, *22*, 19–25, doi:10.1188/18.CJON.S2.19-25.
227. Gonzalez, T. Chemotherapy Extravasations: Prevention, Identification, Management, and Documentation. Clin. J. Oncol. Nurs. **2013**, *17*, 61–66, doi:10.1188/13.CJON.61-66.
228. Goodman, H.A.; Pacheco, C.L.; Loescher, L.J. An Online Intervention to Enhance Nurse Practitioners' Skin Cancer Knowledge, Attitudes, and Counseling Behaviors: A Pilot Study. Journal of the Dermatology Nurses' Association **2018**, *10*, 20–28, doi:10.1097/JDN.0000000000000367.

229. Goodrich TJ; Dyche L Narrative Medicine. Families, Systems & Health: The Journal of Collaborative Family HealthCare **2006**, *24*, 487–488.
230. Gordon, C.; Hendriks, T.; Ranaweera, C.B.; Wallace, M.H.; Ayonrinde, O.T. Characteristics and Outcomes of Octogenerians and Nonagenerians Diagnosed with Colorectal Cancer at a Tertiary Hospital. J. Gastroenterol. Hepatol. **2014**, *29*, 127, doi:10.1111/jgh.12736-7.
231. Gordon, R.; Kasler, M.K.; Stasi, K.; Shames, Y.; Errante, M.; Ciccolini, K.; Skripnik Lucas, A.; Raasch, P.; Fischer-Cartlidge, E. Checkpoint Inhibitors: Common Immune-Related Adverse Events and Their Management. Clin J Oncol Nurs **2017**, *21*, 45–52, doi:10.1188/17.CJON.S2.45-52.
232. Gorzoni, M.L.; Pires, S.L.; De Fátima Costa Faria, L.; Aguado, M.R.V.; Santana, M.C. Human Immunodeficiency Virus in Institutionalized Elderly People. Sao Paulo Med. J. **2016**, *134*, 528–533, doi:10.1590/1516-3180.2016.0034150516.
233. Goulet, V.; Hebert, M.; Hedberg, C.; Laurent, E.; Vaillant, V.; De Valk, H.; Desenclos, J.-C. Incidence of Listeriosis and Related Mortality among Groups at Risk of Acquiring Listeriosis. Clin. Infect. Dis. **2012**, *54*, 652–660, doi:10.1093/cid/cir902.
234. GREEN, I.; STOW, D.; MATTHEWS, F.E.; HANRATTY, B. Changes over Time in the Health and Functioning of Older People Moving into Care Homes: Analysis of Data from the English Longitudinal Study of Ageing. Age & Ageing **2017**, *46*, 693–696, doi:10.1093/ageing/afx046.
235. Griscti, O.; Jacono, J. Effectiveness of Continuing Education Programmes in Nursing: Literature Review. J Adv Nurs **2006**, *55*, 449–456, doi:10.1111/j.1365-2648.2006.03940.x.
236. Gross, J.S.; Neufeld, R.R.; Libow, L.S.; Gerber, I.; Rodstein, M. Autopsy Study of the Elderly Institutionalized Patient. Review of 234 Autopsies. Arch. Intern. Med. **1988**, *148*, 173–176.
237. Gupta, M.; Venkatanarayan, A.; Kadiyam, S. Capnocytophaga - An Unusual Causative Agent for Empyema. Am. J. Respir. Crit. Care Med. 2019, 199.
238. Gurel, L.; Linn, M.W.; Linn, B.S.; Davis, J.E.; Maroney, R.J. Patients in Nursing Homes. Multidisciplinary Characteristics and Outcomes. JAMA **1970**, *213*, 73–77.
239. Hacialioglu, N.; Özer, N.; Yilmaz karabulutlu, E.; Erdem, N.; Erci, B. The Quality of Life of Family Caregivers of Cancer Patients in the East of Turkey. European Journal of Oncology Nursing **2010**, *14*, 211–217, doi:10.1016/j.ejon.2010.01.017.
240. Hanlon, A.; Small, W.; Strauss, J.; Lin, L.L.; Hanisch, L.; Huang, L.; Bai, J.; Wells, J.; Bruner, D.W. Dilator Use After Vaginal Brachytherapy for Endometrial Cancer: A Randomized Feasibility and Adherence Study. Cancer Nursing **2018**, *41*, 200–209, doi:10.1097/NCC.0000000000000500.
241. Hanson, L.C.; Danis, M. Use of Life-Sustaining Care for the Elderly. J Am Geriatr Soc **1991**, *39*, 772–777, doi:10.1111/j.1532-5415.1991.tb02699.x.
242. Hao, Y.; Lang, K.; Huang, H.; Federico, V.; Menzin, J. All-Cause Resource Use among Elderly Breast Cancer Patients by Receipt of Human Epidermal Growth Factor Receptor 2-Targeted Therapy: An Analysis of US National Data 2006-2009. Cancer Res. 2013, 73, doi:10.1158/0008-5472.SABCS13-P3-06-10.
243. Harker, N. Supporting Patients Living with and beyond Cancer in Primary Care. Practice Nursing **2016**, *27*, 431–434, doi:10.12968/pnur.2016.27.9.431.
244. Heilbroner, S.P.; Xanthopoulos, E.; Wang, T.J.C.; Cheng, S. The Effect of Supplemental Estrogen on the Outcomes of NSCLC Patients in the SEER-Medicare Database. J. Clin. Oncol. 2017, 35.
245. Heilbroner, S.P.; Xanthopoulos, E.P.; Wang, T.J.C.; Cheng, S.K. A Population-Based Screen for Repurposed Drugs Using Non-Small Cell Lung Cancer Patients in the Seer-Medicare Database. Int. J. Radiat. Oncol. Biol. Phys. 2017, 99, E460, doi:10.1016/j.ijrobp.2017.06.1704.
246. Heilbroner, S.P.; Xanthopoulos, E.P.; Wang, T.J.C.; Cheng, S.K. Image-Guided Radiation Therapy Does Not Affect Outcomes in a Cohort of Stage III Non-Small Cell Lung Cancer Patients from the Seer-Medicare Database. Int. J. Radiat. Oncol. Biol. Phys. 2017, 99, E460, doi:10.1016/j.ijrobp.2017.06.1703.
247. Helft, P.R. Consultations IN Ethics. Oncology (08909091) **2012**, *26*, 1120–1120.
248. Hewson, V.L. Raising Awareness of Mouth Cancer in the Care Home. Nursing & Residential Care **2018**, *20*, 256–260, doi:10.12968/nrec.2018.20.6.256.
249. Hirai, J.; Kinjo, T.; Haranaga, S.; Fujita, J. Clinical Characteristics of Patients with Community-Acquired Pneumoniae Due to Moraxella Catarrhalis in Adults: A Retrospective Single-Center Study in Okinawa Miyako Island in Japan. Open Forum Infect. Dis. 2018, 5, S448–S449, doi:10.1093/ofid/ofy210.1281.
250. Hoang, C.M.; Davids, J.S.; Maykel, J.A.; Flahive, J.M.; Sturrock, P.R.; Alavi, K. Not All Discharge Settings Are Created Equal: Thirty-Day Readmission Risk after Elective Colorectal Surgery.; United States, 2020; Vol. 63;.
251. Hoerger TJ; Downs KE; Lakshmanan MC; Lindrooth RC; Plouffe L Jr.; Wendling B; West SL; Ohsfeldt RL Healthcare Use among U.S. Women Aged 45 and Older: Total Costs and Costs for Selected Postmenopausal Health Risks. Journal of Women's Health & Gender-Based Medicine **1999**, *8*, 1077–1089, doi:10.1089/jwh.1.1999.8.1077.
252. Holman, K.G.; Noce, J.J.; Montgomery, P.J. Hospice Care: Not Available to Everyone. J Fam Pract **1985**, *20*, 539, 542, 544–546.
253. Honish, A.; Grimsrud, K.; Miedzinski, L.; Gold, E.; Cherry, R.R. Outbreak of Campbell de Morgan Spots in a Nursing Home--Alberta. Can Dis Wkly Rep **1988**, *14*, 211–212.
254. Hopkinson, J.B. Nutritional Support of the Elderly Cancer Patient: The Role of the Nurse. Nutrition **2015**, *31*, 598–602, doi:10.1016/j.nut.2014.12.013.

255. Howell SL; Nelson-Marten P; Krebs LU; Kaszyk L; Wold R Promoting Nurses' Positive Attitudes toward Cancer Prevention/Screening. Journal of Cancer Education **1998**, *13*, 76–84.
256. Hui, E.; Wong, E.M.C.; Woo, J. Assessing Life Expectancies of Institutionalized Older Persons in Hong Kong. Gerontology **2004**, *50*, 165–170, doi:10.1159/000076774.
257. Hunt, R.W.; Fazekas, B.S.; Luke, C.G.; Roder, D.M. Where Patients with Cancer Die in South Australia, 1990-1999: A Population-Based Review. Med. J. Aust. **2001**, *175*, 526–529.
258. Hunt, R.W.; D'Onise, K.; Nguyen, A.-M.T.; Venugopal, K. Where Patients With Cancer Die: A Population-Based Study, 1990 to 2012. J Palliat Care **2019**, *34*, 224–231, doi:10.1177/0825859718814813.
259. Huntington, S.F.; Keshishian, A.; Xie, L.; Baser, O.; McGuire, M. Evaluating the Economic Burden and Health Care Utilization Following First-Line Therapy for Diffuse Large B-Cell Lymphoma Patients in the US Medicare Population. Blood 2016, 128.
260. Hustey, F.M. Care Transitions between Nursing Homes and Emergency Departments: A Failure to Communicate. Ann. Long-Term Care **2010**, *18*, 17–19.
261. I-Chiang Shy; Ching-Yun Han Diagnosing a Cutaneous Horn on an Elderly Woman's Face. Annals of Long Term Care **2014**, *22*, 17–19.
262. Ichihara, T.; Sato, K.; Goto, K. [Comparison of home-care and welfare facilities]. Gan To Kagaku Ryoho 2012, 39 Suppl 1, 115–116.
263. Iida, M.; Masuo, T.; Shigematsu, Y.; Tanaka, M.; Tanaka, K.; Takada, H.; Koh, H.; Tsujimoto, S. A Case of Urothelial Carcinoma with Sarcomatoid Variant of the Urinary Bladder. Acta Cytol. **2016**, *60*, 209–210, doi:10.1159/000446388.
264. Ilgili, Ö.; Demirtaş, Y.; Demir, A.; Arda, B.; Karacalar, A. How to decide from ethical point of view? A malignant melanoma case with dementia. *Turk Geriatr. Derg.* **2006**, *9*, 222–225.
265. Inoue, K.; Tanii, H.; Fukunaga, T.; Abe, S.; Nishimura, F.; Kimura, Y.; Kaiya, H.; Nata, M.; Okazaki, Y. Psychosomatic Tendency for Suicide among the Elderly in Mie Prefecture, Japan. Psychogeriatrics **2007**, *7*, 44–48, doi:10.1111/j.1479-8301.2006.00159.x.
266. Iqbal Penwala, T.; Bin Mohamed Yusof, A.K.; Tan, L.K. Rare Case of Primary Cardiac Lymphoma in Immunocompetent Patient. Eur. Heart J. Cardiovasc. Imaging 2020, 21, i558, doi:10.1093/ehjci/jez319.524.
267. Irvin, S. The Experiences of the Registered Nurse Caring for the Person Dying of Cancer in a Nursing Home. Collegian **2000**, *7*, 30–34, doi:10.1016/s1322-7696(08)60388-1.
268. Iuliano, F.; Iuliano, E.; Perricelli, A.; Pomillo, A.; Luci, M.; Cervo, G. Assisted Administration of Subcutaneous 5-Azacytidine to the Patient's Home. Significant Reduction of Indirect Costs Incurred by Care Recipients and Unpaid Caregivers Together with a Total Adhesion to the Treatment Schedule and Same Safety. Blood 2013, 122.
269. Iuliano, F.; Iuliano, E.; Rossi, M. Assisted Administration of Subcutaneous Rituximab to the Patient's Home. Significant Reduction of Indirect Costs Incurred by Care Recipients and Unpaid Caregivers Together with a Total Adherence to the Treatment Schedule and Same Safety. Blood 2018, 132, doi:10.1182/blood-2018-99-118756.
270. Iuliano, F.; Perricelli, A.; Iuliano, E.; Pomillo, A.; Cervo, G.; Corea, F.; Gammuto, D.; Rella, M.; Salerno, C. Assisted Administration of Subcutaneous 5-Azacytidine to the Patient's Home Is Safe and Cost-Effective. Haematologica **2014**, *99*, 622.
271. Iyer, P.; Ibrahim, M.; Dirweesh, A.; Zaman, M.K. Syndrome of Inappropriate Secretion of ADH (SIADH)-an Unusual Initial Presentation of Non Small Cell Lung Cancer. Am. J. Respir. Crit. Care Med. 2017, 195, doi:10.1164/ajrccmconference.2017.C80H.
272. Jackson, M.L. Transitional Care: Methods and Processes for Transitioning Older Adults With Cancer in a Postacute Setting. Clin J Oncol Nurs **2018**, *22*, 37–41, doi:10.1188/18.CJON.S2.37-41.
273. Jarosek, S.L.; Shippee, T.P.; Virnig, B.A. Place of Death of Individuals with Terminal Cancer: New Insights from Medicare Hospice Place-of-Service Codes. Journal of the American Geriatrics Society **2016**, *64*, 1815–1822, doi:10.1111/jgs.14269.
274. Jayasekera, J.; Onukwugha, E.; Bikov, K.A.; Daniel Mullins, C.; Seal, B.S.; Hussain, A. Incremental Cost (IC) Analysis of Skeletal Related Events (SREs) among Elderly Men with Stage IV Metastatic (M1) Prostate Cancer (PCa). J. Clin. Oncol. 2013, 31.
275. Jiménez-Mola, S.; Idoate, J.; López, M.; Martín, E.; Benítez, C.; Idoate, D.; Plaza, M. In-Hospital Mortality Following Hip Fractures in the Elderly. Eur. Geriatr. Med. 2019, 10, S206, doi:10.1007/s41999-019-00221-0.
276. Johnson, J.C.S.; Byrne, G.J.A. Takotsubo Cardiomyopathy in the Context of Major Neurocognitive Disorder and Multiple Risk Factors. Swiss Arch. Neurol. Psychiatry Psychother. 2019, 170, doi:10.4414/sanp.2019.03022.
277. Johnson, L.M. Self-Governance: Treatment for an Unhealthy Nursing Culture. Health Prog **1987**, *68*, 41–43.
278. Johnson, TaliM. Long-Term Care: Safe Drug Handling of Oral Chemotherapy. consult pharm **2017**, *32*, 74–83, doi:10.4140/TCP.n.2017.74.
279. Johnston G; Gao J; Lavergne R; Rheaume D; Dummer T; MacIntyre M Palliative Radiotherapy: Associations with Age and Nursing Home Residency for Adults Dying of Cancer in Nova Scotia, 2000-2004...17th International Congress on Palliative Care, September 23-26, 2008/Palais Des Congres, Montreal, Canada. Journal of Palliative Care **2008**, *24*, 210–210.
280. Johnston, G.M.; Urquhart, R.; Lethbridge, L.; MacIntyre, M. Increasing Our Understanding of Dying of Breast Cancer: Comorbidities and Care. Prog. Palliative Care **2016**, *24*, 147–152, doi:10.1080/09699260.2015.1108638.
281. Jones, L. The Nutrition of Elderly People. J. Hum. Nutr. Diet. **1999**, *12*, 483, doi:10.1046/j.1365-277x.1999.00199.x.
282. Jones, M. The Common Misdiagnosis That Can Land You in a Nursing Home. Bottom Line Health **2014**, *28*, 1–14.
283. Kabboord, A.D.; Van Eijk, M.; Buijck, B.I.; Koopmans, R.T.C.M.; van Balen, R.; Achterberg, W.P. Comorbidity and Intercurrent Diseases in Geriatric Stroke Rehabilitation: A Multicentre Observational Study in Skilled Nursing Facilities. Eur. Geriatr. Med. **2018**, *9*, 347–353, doi:10.1007/s41999-018-0043-5.

284. Kamel, H.K. Minimizing the Risk of Another Hip Fracture in a Patient with Osteoporosis. Ann. Long-Term Care **2005**, *13*, 23–24.
285. Kamisetty, A.; Magennis, P.; Lowe, D.; Rogers, S.N. Place of Death of Oral and Oropharyngeal Squamous Cell Carcinoma (OO-SCC) Patients 1992-2009: Who Dies at Home? Br. J. Oral Maxillofac. Surg. 2011, 49, S9, doi:10.1016/j.bjoms.2011.04.024.
286. Kamm, A.M.; Turaga, K.K. Appendiceal Mucinous Neoplasm: Nurse Education about a Rare and Complex Disease. Clinical Journal of Oncology Nursing **2020**, *24*, 409–414, doi:10.1188/20.CJON.409-414.
287. Kane, R.L.; Kane, R.A.; Arnold, S.B. Prevention and the Elderly: Risk Factors. Health Serv Res **1985**, *19*, 945–1006.
288. Kanters, A.E.; Nikolian, V.C.; Kamdar, N.S.; Regenbogen, S.E.; Hendren, S.K.; Suwanabol, P.A. Academic Hospitals Discharge Fewer Patients to Postacute Care Facilities After Colorectal Resection. Dis. Colon Rectum **2019**, *62*, 483–490, doi:10.1097/DCR.0000000000001308.
289. Kaplan, M. Hospital Acquired Infections. CLIN.BULL. **1974**, *3*, 134–137.
290. Kardinah, D.; Anderson, B.O.; Duggan, C.; Ali, I.A.; Thomas, D.B. Short Report: Limited Effectiveness of Screening Mammography in Addition to Clinical Breast Examination by Trained Nurse Midwives in Rural Jakarta, Indonesia. Int. J. Cancer **2014**, *134*, 1250–1255, doi:10.1002/ijc.28442.
291. Karlsson, E.; Egenvall, M.; Farahnak, P.; Bergenmar, M.; Nygren-Bonnier, M.; Franzén, E.; Rydwik, E. Better Preoperative Physical Performance Reduces the Odds of Complication Severity and Discharge to Care Facility after Abdominal Cancer Resection in People over the Age of 70 - A Prospective Cohort Study. Eur J Surg Oncol **2018**, *44*, 1760–1767, doi:10.1016/j.ejso.2018.08.011.
292. Kaushik, S.; Hounsome, L.; Blinman, C.; Gornall, R.; Verne, J. The Impact of Sociodemographic Factors and Emergency Admissions on the Place of Death of Gynecological Cancer Patients in England: An Analysis of a National Mortality-Hospital Episode Statistics-Linked Data Set. Int. J. Gynecol. Cancer **2018**, *28*, 1714–1721, doi:10.1097/IGC.0000000000001373.
293. Kelkar, K.; Salvi, R.; Kanitkar, S.; Bejoy, C. Effectiveness of Educational Intervention Regarding Knowledge of Self-Care Management of Diabetes Mellitus during Pandemic COVID 19 among Senior Citizens Residing in Selected Old Age Homes. Eur. J. Mol. Clin. Med. **2021**, *8*, 2363–2373.
294. Kelsey, D.; Zakeri, S.; Hettiaratchi, C.; Offonry, N. Avoidance Behaviours and Missed Opportunities in a Case of Metastatic Squamous Cell Carcinoma. Case Rep. Oncol. Med. 2015, 2015, doi:10.1155/2015/235943.
295. Kennedy, V.E.; Keegan, T.; Li, Q.; Maguire, F.; Muffly, L.S. Intensity of Front-Line Regimen Is Associated with Admissions, in-Hospital Days, and Discharge Destination in Older Adults Withacute Myeloid Leukemia: A Population-Based Analysis. Blood **2020**, *136*, 29, doi:10.1182/blood-2020-141344.
296. Kenny, J.C.; Keenan, P.W. A Survey of Breast Cancer Detection Methods in Long-Term Care Facilities. J Gerontol Nurs **1991**, *17*, 20–22, doi:10.3928/0098-9134-19910401-05.
297. Kenzik, K.M.; Williams, G.R.; Bhatia, S.; Balentine, C.J. Post-Acute Care among Older Adults with Stage I to III Colorectal Cancer. J Am Geriatr Soc **2019**, *67*, 937–944, doi:10.1111/jgs.15680.
298. Khakoo, S.; Georgiou, A.; Hughes, D.; Lanese, A.; Baratelli, C.; Coakley, M.; Shepherd, S.; Calamai, V.; Kouvelakis, K.; Kalaitzaki, R.; et al. Real World Use of Palliative Systemic Therapy (Tx) in Elderly Patients (Pts) with Metastatic Colorectal Cancer (MCRC) within a UK Specialist Cancer Centre. *Ann. Oncol.* 2017, 28, x48, doi:10.1093/annonc/mdx659.020.
299. Khan, S.; Sclabas, G.M.; Lombardo, K.R.; Nagorney, D.M.; Sarr, M.G.; Donohue, J.H.; Kendrick, M.L.; Que, F.G.; Farnell, M.B. Pancreatoduodenectomy for Ductal Adenocarcinoma in the Very Elderly; Is It Safe and Justified? Gastroenterology 2010, 138, S889.
300. Khattak, B.S. Communication in Medicine. CME Bull. Respir. Med. **2000**, *2*, 70.
301. Kiely, D.K.; Flacker, J.M. Common and Gender Specific Factors Associated with One-Year Mortality in Nursing Home Residents. J. Am. Med. Dir. Assoc. **2002**, *3*, 302–309, doi:10.1016/S1525-8610(05)70545-4.
302. King, E.; Rimer, B.K.; Benincasa, T.; Harrop, C.; Amfoh, K.; Bonney, G.; Kornguth, P.; Demark-Wahnefried, W.; Strigo, T.; Engstrom, P. Strategies to Encourage Mammography Use among Women in Senior Citizens' Housing Facilities. J Cancer Educ **1998**, *13*, 108–115, doi:10.1080/08858199809528526.
303. Kissane C Looking after Bob. N2N: Nurse2Nurse **2002**, *2*, 21–23.
304. Kleier, J.A. When Clinical Practice Guidelines And Clinical Practice Diverge. Urologic Nursing **2015**, *35*, 269–270, doi:10.7257/1053-816X.2015.35.6.269.
305. Knox, A. Fall Risk Perceptions: A Study of Hospitalized Patients with Hematologic Malignancies. Clinical Journal of Oncology Nursing 2018, 22, E159–E165, doi:10.1188/18.CJON.E159-E165.
306. Konecne SM; Perdomo M Lymphedema in the Elderly: A Special Needs Population. Topics in Geriatric Rehabilitation **2004**, *20*, 98–113, doi:10.1097/00013614-200404000-00005.
307. Korc-Grodzicki, B.; Wallace, J.A.; Rodin, M.B.; Bernacki, R.E. Cancer in Long-Term Care. Clin Geriatr Med **2011**, *27*, 301–327, doi:10.1016/j.cger.2011.03.011.
308. Kort, H.S.M. Technology for Longevity: Predictors for Use of Technology. Medicina **2016**, *49*, 24–26.
309. Krongrad, A.; Lai, H.; Lai, S. Hospitalization, Inpatient Physical Therapy and Institutionalization after Hospital Discharge of Prostate Cancer Patients in South Florida. J Urol **1998**, *159*, 888–892.
310. Kruizenga, H.M.; Wierdsma, N.J.; van Bokhorst, M.A.E.; Schueren, de van der; Haollander, H.J.; Jonkers-Schuitema, C.F.; van der Heijden, E.; Melis, G.C.; van Staveren, W.A. Screening of Nutritional Status in The Netherlands. Clin Nutr **2003**, *22*, 147–152, doi:10.1054/clnu.2002.0611.

311. Lage, D.E.; DuMontier, C.; Lee, Y.; Nipp, R.D.; Mitchell, S.L.; Temel, J.S.; El-Jawahri, A.; Berry, S.D.; El-Jawahri, A. Potentially Burdensome End-of-Life Transitions among Nursing Home Residents with Poor-Prognosis Cancer. Cancer (0008543X) **2020**, *126*, 1322–1329, doi:10.1002/cncr.32658.
312. Lakasing, E.; Kulkarni, S.; Sparkes, C.; Ravichander, R. A Practice-Based Survey of Patients Dying in Hospital: Can We Do More to Support End-of-Life Care at Home? Br J Community Nurs **2014**, *19*, 130–133, doi:10.12968/bjcn.2014.19.3.130.
313. Lanca, A.; Assis, R.; Gama, P.; Cortes, C.; Paredes, S.; Ferrer, F.; Lobos, A.V. Risk of Progression to End Stage Renal Disease among Geriatric Patients with Acute Kidney Injury Progression to End Stage Renal Disease among Geriatric Patients with Acute Kidney Injury. Nephrol. Dial. Transplant. 2017, 32, iii170, doi:10.1093/ndt/gfx143.
314. Lane, A.; Davis, M.; Wade, J.; Turner, E.; Walsh, E.; Holding, P.; Bonnington, S.; Metcalfe, C.; Martin, R.; Neal, D. Maximising Participant Retention and Outcome Data in a Long Term Cancer Trial (Protect). Trials 2017, 18, doi:10.1186/s13063-017-1902-y.
315. Lang, K.; Hao, Y.; Huang, H.; Federico, V.; Menzin, J. All-Cause Costs among Elderly Breast Cancer Patients by Receipt of Human Epidermal Growth Factor Receptor 2-Targeted Therapy: An Analysis of US National Data 2006-2009. Cancer Res. 2013, 73, doi:10.1158/0008-5472.SABCS13-P3-06-07.
316. Langan, R.C.; Bucciarelli, P.R.; Chapman, W.; Oza, K.; Fehring, C.; Jackson, P.G.; Fishbein, T.M.; Jha, R.; Haddad, N.; Al-Kawas, F.; et al. Pancreaticoduodenectomy in the Oldest of Old: Results beyond Operative Mortality. *HPB* **2014**, *16*, 66–67.
317. Lautenschläger, S. [Therapeutic Nursing in Neurological (Early-)Rehabilitation: A Grounded Theory Study]. Rehabilitation (Stuttg) **2015**, *54*, 273–278, doi:10.1055/s-0035-1555907.
318. Lawday, S.; Keogh, K.; Perkins, D.; Smart, N.; Daniels, I. Decision-Making in the over-90s with Colorectal Cancer. Int. J. Surg. 2017, 47, S28.
319. Lawrence, S.; Robinson, A.; Eagar, K. Identification of the Trajectory of Functional Decline for Advance Care Planning in a Nursing Home Population. Australas J Ageing 2017, 36, E14–E20, doi:10.1111/ajag.12454.
320. Lazzaro M; Thompson M Update on Prostate Cancer Screening. Lippincott's Primary Care Practice **1997**, *1*, 408–420.
321. Le Blanc, T.W.; Seetasith, A.; Choi, M.; Surinach, A.; To, T.M.; Nixon, M.; Montez, M.; Ma, E. Transfusion Burden on Older Patients with Acute Myeloid Leukemia Receiving Low-Intensity Treatments. HemaSphere **2020**, *4*, 798–799, doi:10.1097/HS9.0000000000000404.
322. Le, T.T.; Kuzucan, A.; Simoni-Wastila, L. Validating the Latent Construct of Problematic Opioid Use among Medicare Beneficiaries. Pharmacoepidemiol. Drug Saf. **2018**, *27*, 298, doi:10.1002/pds.4629.
323. Lee, H. Special Issue on Global Health Disparities Focus on Cancer. Asia Pac J Oncol Nurs **2016**, *3*, 309–311, doi:10.4103/2347-5625.195883.
324. Leedo, E.; Gade, J.; Granov, S.; Mellemgaard, A.; Klausen, T.W.; Rask, K.; Astrup, A. The Effect of a Home Delivery Meal Service of Energy- and Protein-Rich Meals on Quality of Life in Malnourished Outpatients Suffering from Lung Cancer: A Randomized Controlled Trial. Nutrition & Cancer **2017**, *69*, 444–453, doi:10.1080/01635581.2017.1283421.
325. Leibovitz, A.; Habot, B.; Walach, N.; Gil, I. [Elderly cancer patients requiring skilled nursing care]. Harefuah **1996**, *131*, 156–160, 216, 215.
326. Leroy, B.; Ahbib, S.; Marot, L.; Vereecken, P. Sequential Treatment with MAL-PDT and Imiquimod (Aldara®) in Difficult-to-Treat Basal Cell Carcinomas: Long-Term Outcome. Melanoma Res. 2011, 21, e59, doi:10.1097/01.cmr.0000399537.31086.16.
327. Levine, S.K.; Sachs, G.A.; Jin, L.; Meltzer, D. A Prognostic Model for 1-Year Mortality in Older Adults after Hospital Discharge. Am. J. Med. **2007**, *120*, 455–460, doi:10.1016/j.amjmed.2006.09.021.
328. Levitt, S.H.; Kempen, P.M.; Mor, V.; Bernabei, R.; Lapane, K.L.; Gambassi, G. Managing Pain in Elderly Patients [1] (Multiple Letters). J. Am. Med. Assoc. **1999**, *281*, 605–606, doi:10.1001/jama.281.7.605.
329. Li, S.; Guo, H.; Peng, Y.; Gong, T.; Fu, A.; Bhowmik, D.; Hernandez, R.K.; Carlson, K.B.; Lowe, K.A.; Rana, J.; et al. Discharge Status and Post-Discharge Healthcare Costs after Skeletal-Related Event Hospitalizations among Medicare Patients with Bone Metastatic Solid Tumors or Multiple Myeloma. *J. Bone Oncol.* 2021, 26, doi:10.1016/j.jbo.2020.100328.
330. Li, T.; Shore, N.D.; Mehra, M.; Todd, M.; Saadi, R.; Leblay, G.; Griffiths, R. The Impact of Subsequent Metastases on Survival and Medical Costs in Prostate Cancer Patients. Eur. Urol. Suppl. 2016, 15, e67.
331. Lindqvist, E.K.; Lund, S.H.; Costello, R.; Burton, D.; Korde, N.; Mailankody, S.; Björkholm, M.; Gudnason, V.; Eiriksdottir, G.; Launer, L.J.; et al. Monoclonal Gammopathy of Undetermined Significance (MGUS) Is Associated with a 30% Increased Risk of Dying at 8 Years of Follow-up: Results from a Screened Cross-Sectional Population-Based Study. Haematologica **2014**, *99*, 115.
332. Lindström, P.; Janzon, L.; Sternby, N.H. Declining Autopsy Rate in Sweden: A Study of Causes and Consequences in Malmö, Sweden. J. Intern. Med. **1997**, *242*, 157–165, doi:10.1046/j.1365-2796.1997.00178.x.
333. Linn, B.S.; Linn, M.W.; Gurel, L. Correlates of Prognosis: A Study of the Physician's Clinical Judgment. Med Care **1973**, *11*, 430–435, doi:10.1097/00005650-197309000-00007.
334. Liu, J.Y.; Bilimoria, K.Y. Surgical Decision Making in Elderly, Impaired Nursing Home Patients. JAMA Surg **2018**, *153*, 1096–1097, doi:10.1001/jamasurg.2018.2737.
335. Liu, S.-H.; Hunnicutt, J.N.; Ulbricht, C.M.; Dubé, C.E.; Hume, A.L.; Lapane, K.L. Adjuvant Use and the Intensification of Pharmacologic Management for Pain in Nursing Home Residents with Cancer: Data from a US National Database. Drugs & Aging **2019**, *36*, 549–557, doi:10.1007/s40266-019-00650-3.
336. Liuu, E.; Guyot, N.; Valero, S.; Jamet, A.; Ouazzani, H.E.; Bouchaert, P.; Tourani, J.; Migeot, V.; Paccalin, M. Prevalence of Cancer and Management in Elderly Nursing Home Residents. A Descriptive Study in 45 French Nursing Homes. European Journal of Cancer Care 2019, 28, N.PAG-N.PAG, doi:10.1111/ecc.12957.

337. Llamas del Castillo, M.D. The Institutionized Elderly's Knowledge on Their Illness and Pharmacological Treatment. Pharm. Care Espana **2001**, *3*, 358–369.
338. Lobo, Y.; Blake, T.; Blake, T.; Muir, J. A Large Fungating Skin Lesion in an Elderly Nursing Home Resident. Aust J Gen Pract **2020**, *49*, 505–506, doi:10.31128/AJGP-12-19-5165.
339. Lopez, J.H.; Reyes-Ortiz, C.A. Subcutaneous Hydration by Hypodermoclysis. Rev. Clin. Gerontol. **2010**, *20*, 105–113, doi:10.1017/S0959259810000109.
340. Lou, X.; Sanders, A.; Sancheti, M.; Javidfar, J.; Pickens, A.; Force, S.; Fernandez, F.; Khullar, O. Safety and Feasibility of Thoracoscopic Lung Resection for Non-Small-Cell Lung Cancer in Octogenarians. Innov. Technol. Tech. Cardiothorac. Vasc. Surg. 2019, 14, 16S-17S, doi:10.1177/1556984519865482.
341. Lovy, M. Pelvic Fragility Fractures: A Debilitating Fracture That Occurs in Elderly Ambulatory Patients with Monoclonal Gammopathy of Uncertain Significance, Pernicious Anemia, Hyponatremia and Is Associated with High Mortality. Osteoporosis Int. 2020, 31, S214, doi:10.1007/s00198-020-05696-3.
342. Lovy, M. Pelvic Ring Fragility Fractures: A Debilitationg Fracture Occurring in Elderly Ambulatory Patients with Monoclonal Gammopathy of Uncertain Significance, Pernicious Anemia, Hyponatremia, and Associated with High Mortality. J. Bone Miner. Res. **2020**, *35*, 223, doi:10.1002/jbmr.4206.
343. Lubeek, S.F.K.; van Gelder, M.M.H.J.; van der Geer, E.R.; van de Kerkhof, P.C.M.; Gerritsen, M.J.P. Skin Cancer Care in Institutionalized Elderly in the Netherlands: A Nationwide Study on the Role of Nursing Home Physicians. J Eur Acad Dermatol Venereol 2016, 30, e236–e237, doi:10.1111/jdv.13573.
344. Lucchetti, G.; Lucchetti, A.L.G.; Pires, S.L.; Gorzoni, M.L. Predictors of Death among Nursing Home Patients: A 5-Year Prospective Study. Geriatr Gerontol Int **2015**, *15*, 234–236, doi:10.1111/ggi.12324.
345. Luce, E.A. Lingering Death from Squamous Cell Carcinoma of the Face. Hosp Pract **1989**, *24*, 60–61, 65–66, 71–72.
346. Ludwick, R. Registered Nurses' Knowledge and Practices of Teaching and Performing Breast Exams among Elderly Women. Cancer Nurs **1992**, *15*, 61–67.
347. Lukas, A.; Mayer, B.; Fialová, D.; Topinkova, E.; Gindin, J.; Onder, G.; Bernabei, R.; Nikolaus, T.; Denkinger, M.D. Pain Characteristics and Pain Control in European Nursing Homes: Cross-Sectional and Longitudinal Results from the Services and Health for Elderly in Long TERm Care (SHELTER) Study. J Am Med Dir Assoc **2013**, *14*, 421–428, doi:10.1016/j.jamda.2012.12.010.
348. Lusardi, M.M. Using Walking Speed in Clinical Practice: Interpreting Age-, Gender-, and Function-Specific Norms. Top. Geriatr. Rehabil. **2012**, *28*, 77–90, doi:10.1097/TGR.0b013e31823d7b9f.
349. Lusky, K.F. Pain Management Found Lacking. Provider **1998**, *24*, 79, 81–82.
350. Maartense, E.; Kluin-Nelemans, H.C.; Noordijk, E.M. Non-Hodgkin's Lymphoma in the Elderly. A Review with Emphasis on Elderly Patients, Geriatric Assessment, and Future Perspectives. Ann. Hematol. **2003**, *82*, 661–670, doi:10.1007/s00277-003-0722-1.
351. Mack, D.S.; Lapane, K.L. Screening Mammography Among Older Women: A Review of United States Guidelines and Potential Harms. Journal of Women's Health (15409996) **2019**, *28*, 820–826, doi:10.1089/jwh.2018.6992.
352. Madden, P.; Coupland, V.; Møller, H.; Davies, E. Using Maps and Funnel Plots to Explore Variation in Place of Death from Cancer within London, 2002-2007. Palliat Med **2011**, *25*, 323–332, doi:10.1177/0269216311398700.
353. Mahalaha, S.A.; Cheruvu, V.K.; Smyth, K.A. Oral Cancer Screening: Practices, Knowledge, and Opinions of Dentists Working in Ohio Nursing Homes. Special Care in Dentistry **2009**, *29*, 237–243, doi:10.1111/j.1754-4505.2009.00105.x.
354. Mahmood, A.; Ghazal, H.; Fink, M.G.; Patel, M.; Atchison, C.; Wei, R.; Suenaert, P.; Pinzone, J.J.; Slasor, P.; Chung, K. Health-Resource Utilization Attributable to Skeletal-Related Events in Patients with Advanced Cancers Associated with Bone Metastases: Results of the US Cohort from a Multicenter Observational Study. Community Oncology **2012**, *9*, 146–157.
355. Maier, R.; Maier, A.; Müller-Busch, C. [Outpatient opiate therapy in cancer patients during their last days of life]. Schmerz **2008**, *22*, 148, 150–155, doi:10.1007/s00482-007-0616-6.
356. Mak, L.; Chan, T.; Chan, F.H.; Liu, S. Gallstone Ileus in an Older Nursing Home Resident. Journal of the American Geriatrics Society **2016**, *64*, 447–449, doi:10.1111/jgs.13965.
357. Mak, T.W.C.; Dai, D.L.K.; Wa Leung, W.; Lee, J.F.Y.; Ng, S.S.M. A Pilot Study of Enhanced Geriatric Input in Management of Elderly Patients Undergoing Colorectal Cancer Surgery. Colorectal Dis. **2014**, *16*, 56, doi:10.1111/codi.12643.
358. Malden, L.T.; Sutherland, C.; Tattersall, M.H.; Morgan, J.; Forsyth, S.; Levi, J.A.; Gunz, F.W. Dying of Cancer. Factors Influencing the Place of Death of Patients. Med. J. Aust. **1984**, *141*, 147–150.
359. Malik, K.S.; Reisner, M. A Fatal Case of Treatment Resistant C. Difficile Colitis. J. Am. Geriatr. Soc. 2012, 60, S192, doi:10.1111/j.1532-5415.2012.04000.x.
360. Malinis, M.; Ngo, T.; Garcia-Diaz, J.; Taimur, S.; Mueller, N.; Eid, A.; Vergidis, P.; Pursell, K.; Bhaskaran, A.; Hoz, R.L.; et al. A Multicenter Study on Clinical Outcomes of Infections within 200 Days of Liver Transplantation among Recipients Age 65 Years and Older. Open Forum Infect. Dis. 2017, 4, S9–S10, doi:10.1093/ofid/ofxl62.024.
361. Maltoni, B.; Forti, P.; Zoli, M.; Maltoni, M.; Ricci, M. Prevalence of Chronic Cancer and No-Cancer Pain in Elderly Hospitalized Patients: Elements for the Early Assessment of Palliative Care Needs. Int. J. Gerontol. **2018**, *12*, 180–185, doi:10.1016/j.ijge.2017.11.005.
362. Manton, K.G.; Liu, K.; Cornelius, E.S. An Analysis of the Heterogeneity of U.S. Nursing Home Patients. J Gerontol **1985**, *40*, 34–46, doi:10.1093/geronj/40.1.34.

363. Marchesi, C.; Speroni, S.; Brivio, L.; Lavazza, M.T.; Mazzone, A. Use of Edoxaban in a Cancer Patient. Ital. J. Med. **2019**, *13*, 90, doi:10.4081/itjm.2019.s2.
364. Marie Thon Aamodt, I.; Lie, I.; Hellesø, R. Nurses' Perspectives on the Discharge of Cancer Patients with Palliative Care Needs from a Gastroenterology Ward. International Journal of Palliative Nursing **2013**, *19*, 396–402, doi:10.12968/ijpn.2013.19.8.396.
365. Marlies Heiligenberg, M.; Maaskant, J.M.; Latour, C.H.M. Differences in Comorbidities between Men and Women with Heart Failure Exist. Eur. J. Heart Fail. **2017**, *19*, 268, doi:10.1002/ejhf.833.
366. Maroz, N.; Mohandes, S.; Field, H.; Kabakov, Z.; Simman, R. Calciphylaxis in Patients with Preserved Kidney Function. J. Am. Coll. Clin. Wound Spec. **2014**, *6*, 24–28, doi:10.1016/j.jccw.2015.08.002.
367. Marrie, T.J. Pneumonia. Bailliere's Clin. Infect. Dis. **1998**, *5*, 35–51.
368. Marriott, L.K.; Lipus, A.C.; Choate, L.; Smith, J.; Coppola, L.; Cameron, W.E.; Shannon, J. Experiential Learning Through Participatory Action Research in Public Health Supports Community-Based Training of Future Health Professionals. Pedagogy Health Promot **2015**, *1*, 220–232, doi:10.1177/2373379915601119.
369. Marshall, L.L. Megestrol Acetate Therapy in Geriatric Patients: Case Reviews and Associated Deep Vein Thrombosis. Consult. Pharm. **2003**, *18*, 764–773.
370. Martin, E.J. A High-Priority Population: Older Patients Require Extra Care and Understanding. AGD Impact **2018**, *46*, 12–17.
371. Martoni, A.A.; Degli Esposti, C.; Cricca, A.; Rocchi, G.; Giaquinta, S. Twice-Daily Pain Monitoring as Standard Clinical Practice for Inpatients at a Medical Oncology Unit: A Descriptive Study. Ann. Oncol. **2007**, *18*, 158–162, doi:10.1093/annonc/mdl314.
372. Maruthachalam, K.; Stoker, E.; Nicholson, G.; Horgan, A.F. Nurse Led Flexible Sigmoidoscopy in Primary Care - The First Thousand Patients. Colorectal Dis. **2006**, *8*, 557–562, doi:10.1111/j.1463-1318.2006.00973.x.
373. Masoorli, S. Education, Education, and More Education. JAVA J. Assoc. Vasc. Access **2007**, *12*, 128+130, doi:10.2309/java.12-3-7.
374. Maule, W.F. Screening for Colorectal Cancer by Nurse Endoscopists. NEW ENGL. J. MED. **1994**, *330*, 183–187, doi:10.1056/NEJM199401203300307.
375. McCullough, S.; Reckrey, J.; Shah, B. Dysphagia with a Large Goiter: What You See Is Not Necessarily What You Get. J. Am. Geriatr. Soc. 2012, 60, S78–S79, doi:10.1111/j.1532-5415.2012.04000.x.
376. McLane, L.; Jones, K.; Lydiatt, W.; Lydiatt, D.; Richards, A. Taking Away the Fear: A Grounded Theory Study of Cooperative Care in the Treatment of Head and Neck Cancer. Psychooncology **2003**, *12*, 474–490, doi:10.1002/pon.670.
377. McLeroy, T.M.; Gurenlian, J.; Rogo, E.J. The Effect of Continuing Education on Dental Hygienists' Knowledge, Attitudes, and Practices Regarding Human Papillomavirus Related Oropharyngeal Cancer. Journal of Dental Hygiene **2020**, *94*, 16–28.
378. McMillan, A.; Mentnech, R.M.; Lubitz, J.; McBean, A.M.; Russell, D. Trends and Patterns in Place of Death for Medicare Enrollees. Health Care Financ Rev **1990**, *12*, 1–7.
379. Meijers, J.M.M.; Schols, J.M.G.A.; van Bokhorst-de van der Schueren, M.A.E.; Dassen, T.; Janssen, M.A.P.; Halfens, R.J.G. Malnutrition Prevalence in The Netherlands: Results of the Annual Dutch National Prevalence Measurement of Care Problems. Br. J. Nutr. **2009**, *101*, 417–423, doi:10.1017/S0007114508998317.
380. Melac, A.T.; Lesuffleur, T.; Bousquet, P.-J.; Fagot-Campagna, A.; Gastaldi-Ménager, C.; Tuppin, P. Cancer and End of Life: The Management Provided during the Year and the Month Preceding Death in 2015 and Causes of Death in France. Supportive Care in Cancer **2020**, *28*, 3877–3887, doi:10.1007/s00520-019-05188-z.
381. Meraviglia MG The Mediating Effects of Meaning in Life and Prayer on the Physical and Psychological Responses of People Experiencing Lung Cancer., University of Texas at Austin, 2001.
382. Mertens, J.; Haddad, M.; De Block, C. CREST Syndrome Diagnosed Due to Faulty Point-of-Care Glycemia: A Case Report. Acta Clin. Belg. **2020**, *74*, 15–16, doi:10.1080/17843286.2019.1687975.
383. Messer, M.; Hackenberg, A. [From case to case: What really counts]. Pflege Z **2006**, *59*, 518–520.
384. Messer, M.; Weber, M. [From case to case: Making a decision about life]. Pflege Z **2008**, *61*, 290–291.
385. Meyer-Kühling, I.; Eckardt, R.; Makrantonaki, E. Professional Caregivers' Knowledge in Dermatology: Improving Knowledge and Communication to Dermatologists in Geriatric Facilities. Z Gerontol Geriat **2018**, *51*, 54–59, doi:10.1007/s00391-016-1132-z.
386. Miller, M.B. Decision-Making in the Death Process of the Ill Aged. Geriatrics **1971**, *26*, 105–116.
387. Miller, S.C.; Intrator, O.; Gozalo, P.; Roy, J.; Barber, J.; Mor, V. Government Expenditures at the End of Life for Short- and Long-Stay Nursing Home Residents: Differences by Hospice Enrollment Status. J Am Geriatr Soc **2004**, *52*, 1284–1292, doi:10.1111/j.1532-5415.2004.52357.x.
388. Minarik, J.; Jungova, A.; Straub, J.; Gregora, E.; Bryknarova, J.; Nemcova, B.; Hajek, R. The Role of Demographic and Social Factors on Decision Making in Patients with Relapsed and Refractory Multiple Myeloma Treated by Ixazomib, Lenalidomide and Dexamethasone. Blood 2019, 134, doi:10.1182/blood-2019-130961.
389. Mirza, Z.; Roman-Colon, D.; Ortiz-Rivera, M.; Martínez-Souss, J. Old Is Gold: Cecocostomy for Recurrent Colonic Pseudo-Obstruction. Am. J. Gastroenterol. 2020, 115, S1073–S1074, doi:10.14309/01.ajg.0000710228.09158.e8.
390. Missel, M.; Schønau, M.N.; Pedersen, J.H.; Pedersen, P.U. Transition from Hospital to Daily Life: A Pilot Study. Rehabil Nurs **2015**, *40*, 20–29, doi:10.1002/rnj.180.
391. Mitchell, M.A.; Dhaliwal, I.; Mulpuru, S.; Amjadi, K.; Chee, A. Early Readmission to Hospital in Patients With Cancer With Malignant Pleural Effusions: Analysis of the Nationwide Readmissions Database. Chest **2020**, *157*, 435–445, doi:10.1016/j.chest.2019.09.007.
392. Mitchell, S.L.; Teno, J.M.; Miller, S.C.; Mor, V. A National Study of the Location of Death for Older Persons with Dementia. J Am Geriatr Soc **2005**, *53*, 299–305, doi:10.1111/j.1532-5415.2005.53118.x.

393. Mohri, T.; Ueda, A.; Katsume, Y.; Nagaoka, M.; Momose, Y.; Nonoguchi, N.; Hoshida, K.; Togashi, I.; Sato, T.; Isogaya, K.; et al. PRIMARY CARDIAC B CELL LYMPHOMA FROM SINUS NODE, PRESENTING AS SICK SINUS SYNDROME. Heart Rhythm **2019**, *16*, 421, doi:10.1016/j.hrthm.2019.04.017.
394. Mollica, M.A.; Litzelman, K.; Rowland, J.H.; Kent, E.E. The Role of Medical/Nursing Skills Training in Caregiver Confidence and Burden: A CanCORS Study. Cancer **2017**, *123*, 4481–4487, doi:10.1002/cncr.30875.
395. Mongil PL; Mongil RL Evaluation of the Neoplastic Condition and Its Impact on the Functional Status of the Institutionalized Elderly Patient. Gerokomos **2001**, *12*, 163–172.
396. Montgomery, M.; Cheshire, M.; Johnson, P.; Beasley, A. Incorporating End-of-Life Content Into the Community Health Nursing Curriculum Using High-Fidelity Simulation. Journal of Hospice & Palliative Nursing **2016**, *18*, 60–65, doi:10.1097/NJH.0000000000000211.
397. Morin, L.; Wastesson, J.; Fors, S.; Johnell, K. How Do Hundred-Year-Old People Die? Results from a Populationlevel Study of Centenarians in Sweden. Eur. Geriatr. Med. 2018, 9, S168, doi:10.1007/s41999-018-0097-4.
398. Motiwala, S.S.; Croxford, R.; Guerriere, D.N.; Coyte, P.C. Predictors of Place of Death for Seniors in Ontario: A Population-Based Cohort Analysis. Can J Aging **2006**, *25*, 363–371, doi:10.1353/cja.2007.0019.
399. Moutzouris, J.P.; Chow, V.; Yong, A.S.C.; Chung, T.; Naganathan, V.; Kritharides, L.; Chwan, A.C. Acute Pulmonary Embolism in Individuals Aged 80 and Older. J Am Geriatr Soc **2014**, *62*, 2004–2006, doi:10.1111/jgs.13063.
400. Mrayyan, M.T. Nurses' Views of Organizational Readiness for Change. Nurs Forum **2020**, *55*, 83–91, doi:10.1111/nuf.12393.
401. Mubarak, M.; Daglilar, E.S.; Similari, T.; Ray, A. Metastatic Renal Cell Carcinoma Presenting as a Bleeding Gastric Polyp. Am. J. Gastroenterol. 2017, 112, S1042–S1043, doi:10.1038/ajg.2017.318.
402. Mulligan, R. The Three Phases of Eve: Exploring the Common and Unique Findings in Oral and Systemic Health of Differently Aging Women. Compend Contin Educ Dent **2002**, *23*, 32–40.
403. Murphy, D.P.; Istenes, N.A. Strategies to Promote Successful Aging: Part 2, the Physician's Role. Inside: Preventive Strategies That Help Your Patients Stay in Good Shape... Mentally and Physically. Consultant **2005**, *45*, 653–658.
404. Musat, A.; Cailliod, R.; Bismuth, M.-J.; Bernard, A.; Quantin, C. Medico economic impact of standardised procedures after pulmonary lobectomies in lung cancer patients. *J. Econ. Med.* **2005**, *23*, 127–138.
405. Mygind-Klausen, T.; Lauridsen, K.G.; Bødker, H.; Løfgren, B. Emergency Medical Technicians Are Often Consulted on Termination of Resuscitation, and Will Terminate Resuscitation Based on Controversial Single Factors. Circulation 2016, 134.
406. Nagamatsu, Y.; Natori, Y.; Yanai, H.; Horiuchi, S. Impact of a Nursing Education Program about Caring for Patients in Japan with Malignant Pleural Mesothelioma on Nurses' Knowledge, Difficulties and Attitude: A Randomized Control Trial. Nurse Educ Today **2014**, *34*, 1087–1093, doi:10.1016/j.nedt.2014.02.007.
407. Nagamine, M.; Osman, S.; Chen, N.; Akbari, Y.; Mnatsakanya, L.; Korb, M.; Mozaffar, T. Covid-19 Presenting as Post-Infectious Myelitis: A Case Report. Ann. Neurol. 2020, 88, S57, doi:10.1002/ana.25865.
408. Nakamura, H.; Yoon, W.T.; Kubota, Y.; Nishihara, K. Resumption of Oral Intake after Gastroduodenal Stenting in Patients with Malignant Gastric Outlet Obstruction. J. Gastroenterol. Hepatol. **2017**, *32*, 81, doi:10.1111/jgh.13875.
409. Nakazawa, N. Difficult Peripherally Inserted Central Catheter (PICC) Insertions. JAVA J. Assoc. Vasc. Access **2005**, *10*, 112–122, doi:10.2309/java.10-3-6.
410. Navon, L.; Morag, A. Advanced Prostate Cancer Patients' Relationships with Their Spouses Following Hormonal Therapy. Eur. J. Oncol. Nurs. **2003**, *7*, 73–80, doi:10.1016/S1462-3889(03)00022-X.
411. Nazarko, L. Improving Skin Cancer Awareness in Care Homes. Nursing & Residential Care **2018**, *20*, 10–12, doi:10.12968/nrec.2018.20.1.10.
412. Needham, D.S.; Wong, I.C.K.; Campion, P.D. Evaluation of the Effectiveness of UK Community Pharmacists' Interventions in Community Palliative Care. Palliative Med. **2002**, *16*, 219–225, doi:10.1191/0269216302pm533oa.
413. Ng, A.; Nadarajan, V.; McIver, S.; Reid, C.; Schofield, E.; Sachar, A. Frequent Attendances to a London Emergency Department: A Service Improvement Project Embedding Mental Health into the Team. London J. Prim. Care **2015**, *7*, 70–77, doi:10.1080/17571472.2015.11493438.
414. Nichols J Medical Ethics. Ethics Means Respecting the Individual When a Person's Desires Are Unclear. Caring for the Ages **2008**, *9*, 19–19, doi:10.1016/s1526-4114(08)60099-2.
415. Nieder, C.; Norum, J.; Stemland, J.G.; Dalhaug, A. Resource Utilization in Patients with Brain Metastases Managed with Best Supportive Care, Radiotherapy and/or Surgical Resection: A Markov Analysis. Oncology **2010**, *78*, 348–355, doi:10.1159/000320347.
416. Nielsen, L.K.; King, M.; Möller, S.; Jarden, M.; Andersen, C.L.; Frederiksen, H.; Gregersen, H.; Klostergaard, A.; Steffensen, M.S.; Pedersen, P.T.; et al. Strategies to Improve Patient-Reported Outcome Completion Rates in Longitudinal Studies. *Qual. Life Res.* **2020**, *29*, 335–346, doi:10.1007/s11136-019-02304-8.
417. Niessen, L.C.; Fedele, D.J. Older Adults--Implications for Private Dental Practitioners. J Calif Dent Assoc **2005**, *33*, 695–703.
418. Niznik, J.D.; Zhang, S.; Mor, M.K.; Zhao, X.; Ersek, M.; Aspinall, S.L.; Gellad, W.F.; Thorpe, J.M.; Hanlon, J.T.; Schleiden, L.J.; et al. Adaptation and Initial Validation of Minimum Data Set (MDS) Mortality Risk Index to MDS Version 3.0. Journal of the American Geriatrics Society **2018**, *66*, 2353–2359, doi:10.1111/jgs.15579.
419. Nørgaard, B.; Lee, A. Characteristics of Patients Receiving District-Nursing Assistance for Anticoagulant Therapy: A Descriptive Cohort Study. Eur. Geriatr. Med. **2017**, *8*, 256–259, doi:10.1016/j.eurger.2017.04.014.

420. Norlander, O.P. Management of Respirator and Anesthesia Patients: Monitoring and Developments. MED. PROG. TECHNOL. **1974**, *3*, 15–23.
421. Notsu, M.; Naito, T.; Mori, K.; Notsu, A.; Morikawa, A.; Kawabata, T.; Okayama, T.; Yonenaga, Y.; Sugiyama, M.; Kenmotsu, H.; et al. Days Spent at Home near the End of Life in Japanese Elderly Patients with Lung Cancer: Post Hoc Analysis of a Prospective Study. *Asia-Pacific Journal of Oncology Nursing* **2021**, *8*, 228–236, doi:10.4103/2347-5625.311131.
422. O'Connor, S.; Jolliffe, S.; Stanmore, E.; Renwick, L.; Booth, R. Social Media in Nursing and Midwifery Education: A Mixed Study Systematic Review. J Adv Nurs **2018**, *74*, 2273–2289, doi:10.1111/jan.13799.
423. O'Grady ET From the Desk of Eileen T. O'Grady, PhD, RN, NP. Meaningful Prevention. Nurse Practitioner World News **2008**, *13*, 1–14.
424. O'Neal, P.V.; McClellan, L.C.; Jarosinski, J.M. A New Model in Teaching Undergraduate Research: A Collaborative Approach and Learning Cooperatives. Nurse Educ Pract **2016**, *18*, 80–84, doi:10.1016/j.nepr.2016.03.008.
425. Obeidat, N.A.; Onukwugha, E.; Bikov, K.; Seal, B.; Mullins, C.D. Costs of Care for Elderly Metastatic Prostate Cancer Patients over Time. Value Health 2009, 12, A47, doi:10.1111/j.1524-4733.2009.00537-2.x.
426. Odejide, O.O.; Cronin, A.M.; Davidoff, A.; LaCasce, A.S.; Abel, G.A. Comparative Effectiveness of Treatments for Limited Stage, Non-Bulky Diffuse Large B-Cell Lymphoma in the Elderly. Blood 2013, 122.
427. Ohbayashi K; Kataoka Y; Suzuki K Breast Awareness Promotion: Development, Implementation and Evaluation of an Educational Program for Nurse-Midwives. Journal of St. Luke's Society for Nursing Research **2009**, *13*, 8–16.
428. Öhlén, J.; Cohen, J.; Håkanson, C. Determinants in the Place of Death for People with Different Cancer Types: A National Population-Based Study. Acta Oncologica **2017**, *56*, 455–461, doi:10.1080/0284186X.2016.1250946.
429. Ohson, K.; DesGroseilliers, J.-P.; Weatherhead, S.; Weatherhead, L. Imiquimod 5% Cream Use for the Treatment of Basal Cell Carcinomas in Elderly Patients, in Long-Term Care Facilities, Not Amenable to Surgical or Radiation Therapy. J Cutan Med Surg **2006**, *10*, 201–203, doi:10.2310/7750.2006.00033.
430. Olszewski, A.J. Comparative Outcomes of Splenectomy and Rituximab-Based Chemotherapy in Elderly Patients with Splenic Marginal Zone Lymphoma. J. Clin. Oncol. 2013, 31.
431. Onder, G.; Vetrano, D.L.; Villani, E.R.; Carfì, A.; Lo Monaco, M.R.; Cipriani, M.C.; Manes Gravina, E.; Denkinger, M.; Pagano, F.; van der Roest, H.G.; et al. Deprescribing in Nursing Home Residents on Polypharmacy: Incidence and Associated Factors. *Journal of the American Medical Directors Association* **2019**, *20*, 1116–1120, doi:10.1016/j.jamda.2019.01.130.
432. Orebo Holmström, M.; Gimsing, P.; Abildgaard, N.; Andersen, N.; Clausen, N.; Frederiksen, M.; Gregersen, H.; Kristensen, D.; Larsen, H.; Trøllund Pedersen, P.; et al. Causes of Death 30 and 180 Days after Diagnosis in Non-HDT Treated Danish Multiple Myeloma Patients: A Study from the Danish Myeloma Study Group. Haematologica **2013**, *98*, 323.
433. Ørum, M.; Jensen, K.; Meldgaard, P.; Damsgaard, E.M. Frailty Status of Elderly Cancer Patients in the Central Denmark Region. Eur. Geriatr. Med. 2015, 6, S76.
434. Owan, T.; Kohra, T.; Miyara, Y.; Kohki, M.; Shimo, Y.; Bise, T.; Sugaya, K.; Kiriyama, M.; Sunagawa, Y.; Kohno, S. Urination Assessment after the Removal of Bladder Catheter Using a Novel Urination Chart. Nurs. Health Sci. **2003**, *5*, 189–197, doi:10.1046/j.1442-2018.2003.00152.x.
435. Panchal, K.; Hussain, M.; Ramaswamy, S. THE CASE OF A DISGUISED PNEUMOTHORAX. Chest 2019, 156, A2243, doi:10.1016/j.chest.2019.08.2162.
436. Parhar, H.S.; Chang, B.A.; Durham, J.S.; Anderson, D.W.; Hayden, R.E.; Prisman, E. Post-Acute Care Use after Major Head and Neck Oncologic Surgery with Microvascular Reconstruction. Laryngoscope **2018**, *128*, 2532–2538, doi:10.1002/lary.27190.
437. Parsons, Y. An Ounce of Prevention. Screening Can Improve Both Quantity and Quality of Life. Contemp Longterm Care **1995**, *18*, 50–52.
438. Passik SD; Donaghy KB; Theobald DE; Lundberg JC; Holtsclaw E; Dugan WM Jr.; Passik, S.D.; Donaghy, K.B.; Theobald, D.E.; Lundberg, J.C.; et al. Oncology Staff Recognition of Depressive Symptoms on Videotaped Interviews of Depressed Cancer Patients: Implications for Designing a Training Program. Journal of Pain & Symptom Management **2000**, *19*, 329–338.
439. Pastor Ochoa, C.; Herraiz, J.; Hernandez, A.; Morago, M.; Garcia Garcia, R. The Real Degree in Terminal Patients and Special Knowledge about the Diagnosis of Medical Profile Who Are Living in Nurse Home. Eur. Geriatr. Med. 2011, 2, S48, doi:10.1016/j.eurger.2011.06.002.
440. Patil, S.; Chamberlain, R.S. Whipple Procedure in Octogenarians and Nonagenarians: A United States Population-Based Study Analyzing Morbidity and Mortality Following Whipple Procedure in Elderly Patients (National Inpatient Sample Database 1998-2010). J. Am. Coll. Surg. 2015, 221, e108–e109.
441. Pawar, A.; Desai, R.; Gautam, N.; Kim, S.C. Risk of Serious Infections in Tofacitinib versus Other Biologic Drug Initiators in Patients with Rheumatoid Arthritis: A Multi-Database Cohort Study. Arthritis Rheum. **2019**, *71*, 5011–5013, doi:10.1002/art.41108.
442. Pawar, A.; Desai, R.; He, M.; Bessette, L.; Kim, S. Risk of Non-Vertebral Fractures among Rheumatoid Arthritis Patients Treated with Biologic or Targeted-Synthetic DMARDs: A Multi-Database Comparative Safety Study. Arthritis Rheum. **2020**, *72*, 2009–2011, doi:10.1002/art.41538.
443. Pearl, J.A.; Patil, D.; Filson, C.P.; Arya, S.; Alemozaffar, M.; Master, V.A.; Ogan, K. Patient Frailty and Discharge Disposition Following Radical Cystectomy. Clin Genitourin Cancer 2017, 15, e615–e621, doi:10.1016/j.clgc.2016.12.013.
444. Penn, C.A.; Kamdar, N.S.; Morgan, D.M.; Uppal, S. Preoperatively Predicting Non-Home Discharge after Surgery for Gynecologic Malignancy. Gynecol. Oncol. **2017**, *145*, 15–16, doi:10.1016/j.ygyno.2017.03.054.

445. Percival, J.; Abbott, K.; Allain, T.; Bradley, R.; Cramp, F.; Donovan, J.L.; McCabe, C.; Neubauer, K.; Redwood, S.; Cotterill, N. "We Tend to Get Pad Happy": A Qualitative Study of Health Practitioners' Perspectives on the Quality of Continence Care for Older People in Hospital. BMJ Open Qual 2021, 10, doi:10.1136/bmjoq-2021-001380.
446. Perkins RB; Langrish S; Stern LJ; Simon CJ A Community-Based Education Program about Cervical Cancer Improves Knowledge and Screening Behavior in Honduran Women. Revista Panamericana de Salud Publica **2007**, *22*, 187–193, doi:10.1590/s1020-49892007000800005.
447. Pietrokovski, J.; Levy, F.; Azuelos, J.; Tau, S.; Tamari, I.; Mostavoy, R. Oral Findings in Elderly Nursing Home Residents in Selected Countries. 2. Soft Tissue Lesions and Denture Wearing Habits. Gerodontology **1990**, *9*, 75–81, doi:10.1111/j.1741-2358.1990.tb00262.x.
448. Pijpe, A.; Slottje, P.; van Pelt, C.; Stehmann, F.; Kromhout, H.; van Leeuwen, F.E.; Vermeulen, R.C.H.; Rookus, M.A. The Nightingale Study: Rationale, Study Design and Baseline Characteristics of a Prospective Cohort Study on Shift Work and Breast Cancer Risk among Nurses. BMC Cancer 2014, 14, doi:10.1186/1471-2407-14-47.
449. Pita Campos, Á. Cuidados Paliativos En Paciente Con Adenocarcinoma Pancreático En Estadio Terminal. Etica de los Cuidados **2017**, *10*, 1–10.
450. Poulin, C.; Schlech, W.F. 3rd A Pseudo-Outbreak in a Nursing Home. Infect Control Hosp Epidemiol **1991**, *12*, 521–522, doi:10.1086/646401.
451. Powell, D.; Pollack, M.; Moretz, C.; Robinson, S.; Parente, A.; Xi, A.; Dreyfus, J.; Make, B.; Feigler, N. Assessment of Healthcare Resource Use by Comorbidity Burden among COPD Patients Diagnosed across Multiple Payer Settings. J. Manag. Care Spec. Pharm. 2020, 26, S58.
452. Qadri, S. Rare Cutaneous Melanoma in a Centenarian. J. Am. Med. Informatics Assoc. 2018, 19, B8, doi:10.1016/j.jamda.2017.12.032.
453. Rajkumar, A.; Baum, E. Cerebral Venous Sinus Thrombosis Resulting from Herpes Zoster Infection in an Older Adult. Ann. Long-Term Care **2016**, *24*, 27–30.
454. Ramfelt E; Severinsson E; Lützén K Attempting to Find Meaning in Illness to Achieve Emotional Coherence: The Experiences of Patients with Colorectal Cancer. Cancer Nursing **2002**, *25*, 141–149.
455. Ramkumar, P.N.; Gwam, C.; Navarro, S.M.; Haeberle, H.S.; Karnuta, J.M.; Delanois, R.E.; Mont, M.A. Discharge to the Skilled Nursing Facility: Patient Risk Factors and Perioperative Outcomes after Total Knee Arthroplasty. Ann. Transl. Med. 2019, 7, doi:10.21037/atm.2018.12.62.
456. Ramudu, L.; Bellet, B.; Higgs, J.; Latimer, C.; Smith, R. How Effectively Do We Use Double Staff Time? Aust J Adv Nurs **1994**, *11*, 5–10.
457. Rand, K.L.; Cohee, A.A.; Monahan, P.O.; Wagner, L.I.; Shanahan, M.L.; Champion, V.L. Coping Among Breast Cancer Survivors: A Confirmatory Factor Analysis of the Brief COPE. Journal of Nursing Measurement **2019**, *27*, 259–276, doi:10.1891/1061-3749.27.2.259.
458. Rangarajan, S. Leptomeningeal Carcinomatosis from Esophogeal Cancer Presenting with Dysphagia. J. Am. Geriatr. Soc. 2017, 65, S95, doi:10.1111/(ISSN)1445-5994.
459. Rawlings, C.; Cameron, A.; Zaimin, W.; Healy, H.; Hoy, W. Comparing Those with CKD (Chronic Kidney Diease) Planed for Renal Supportive Care (RSC) and Those for Renal Replacement Therapy (RRT). Nephrology **2016**, *21*, 178, doi:10.1111/nep.12888.
460. Reblin, M.; Clayton, M.F.; Xu, J.; Hulett, J.M.; Latimer, S.; Donaldson, G.W.; Ellington, L. Caregiver, Patient, and Nurse Visit Communication Patterns in Cancer Home Hospice. Psycho-Oncology **2017**, *26*, 2285–2293, doi:10.1002/pon.4361.
461. Redaniel, M.T.; Laudico, A.; Mirasol-Lumague, M.R.; Gondos, A.; Uy, G.; Brenner, H. Inter-Country and Ethnic Variation in Colorectal Cancer Survival: Comparisons between a Philippine Population, Filipino-Americans and Caucasians. BMC Cancer 2010, 10, doi:10.1186/1471-2407-10-100.
462. Reddy, V.; Nguyen, K.P.; Faiz, S.; Glass, W.; Kala, J.; Zhang, S. RENAL METASTASIS - RARE INITIAL PRESENTATION OF OVARIAN CANCER. Am. J. Kidney Dis. **2020**, *75*, 624, doi:10.1053/j.ajkd.2020.02.306.
463. Reichman, W. Late Life Depression and Dementia. Prim. Care Update Ob Gyns **2003**, *10*, 238–241, doi:10.1016/S1068-607X(03)00048-9.
464. Reilev, M.; Lundby, C.; Hoffmann, H.; Larsen, S.P.; Jensen, J.; Pottegard, A. Drug Use among Older People Admitted to Nursing Homes. Pharmacoepidemiol. Drug Saf. **2019**, *28*, 287–288, doi:10.1002/pds.4864.
465. Reinke, L.F.; Vig, E.K.; Tartaglione, E.V.; Backhus, L.M.; Gunnink, E.; Au, D.H. Protocol and Pilot Testing: The Feasibility and Acceptability of a Nurse-Led Telephone-Based Palliative Care Intervention for Patients Newly Diagnosed with Lung Cancer. Contemp. Clin. Trials **2018**, *64*, 30–34, doi:10.1016/j.cct.2017.11.013.
466. Reintjes, W.; Romijn, M.D.M.; Hollander, D.; Ter Bruggen, J.P.; van Marum, R.J. Reversible Dementia: Two Nursing Home Patients with Voltage-Gated Potassium Channel Antibody-Associated Limbic Encephalitis. J Am Med Dir Assoc **2015**, *16*, 790–794, doi:10.1016/j.jamda.2015.06.008.
467. Rhondali, W.; Hui, D.; Kim, S.H.; Kilgore, K.; Kang, J.H.; Nguyen, L.; Bruera, E. Association between Patient-Reported Symptoms and Nurses' Clinical Impressions in Cancer Patients Admitted to an Acute Palliative Care Unit. J. Palliative Med. **2012**, *15*, 301–307, doi:10.1089/jpm.2011.0403.

468. Riese, C.; Weiß, B.; Borges, U.; Beylich, A.; Dengler, R.; Hermes-Moll, K.; Welslau, M.; Baumann, W. Effectiveness of a Standardized Patient Education Program on Therapy-Related Side Effects and Unplanned Therapy Interruptions in Oral Cancer Therapy: A Cluster-Randomized Controlled Trial. Supportive Care Cancer **2017**, *25*, 3475–3483, doi:10.1007/s00520-017-3770-0.
469. Rigler, S.K.; Shireman, T.I.; Kallenbach, L. Predictors of Long-Acting Opioid Use and Oral versus Transdermal Route among Older Medicaid Beneficiaries. Am J Geriatr Pharmacother **2007**, *5*, 91–99, doi:10.1016/j.amjopharm.2007.05.002.
470. Roder, D.; Bonett, A.; Hunt, R.; Beare, M. Where Patients with Cancer Die in South Australia. Med. J. Aust. **1987**, *147*, 11–13.
471. Rodin, M.B. Should You Screen Nursing Home Residents for Cancer? J Geriatr Oncol **2017**, *8*, 154–159, doi:10.1016/j.jgo.2016.10.005.
472. Romanoski M Letter to the Editor. Plastic Surgical Nursing **2005**, *25*, 115–115.
473. Rosenberg, K. JOURNAL Watch. AJN American Journal of Nursing **2015**, *115*, 56–57.
474. Rossi, N.A.; Rawl, J.W.; Chaaban, M.R. Retroperitoneal Hematoma as a Complication of Endovascular Embolization of Tumor Epistaxis. Otolaryngol. Head Neck Surg. 2020, 163, P302, doi:10.1177/0194599820934781.
475. Roulston, A.; Campbell, A.; Cairnduff, V.; Fitzpatrick, D.; Donnelly, C.; Gavin, A. Bereavement Outcomes: A Quantitative Survey Identifying Risk Factors in Informal Carers Bereaved through Cancer. Palliative Medicine **2017**, *31*, 162–170, doi:10.1177/0269216316649127.
476. Rudel RJ On Breast Cancer Detection, Directors of Nursing and Female Residents: A Study in Rural Long-Term Care. Online Journal of Rural Nursing & Health Care **2007**, *7*, 21–35, doi:10.14574/ojrnhc.v7i2.135.
477. Ruelle, L.; Deyi, V.Y.M.; Konopnicki, D.; Dauby, N. Saccharomyces Cerevisiae Fungemia: Risk Factors, Outcome and Link with Entérol® Administration. Acta Clin. Belg. Int. J. Clin. Lab. Med. **2018**, *73*, 8, doi:10.1080/17843286.2018.1538724.
478. Rys, S.; Mortier, F.; Deliens, L.; Bilsen, J. The Practice of Continuous Sedation until Death in Nursing Homes in Flanders, Belgium: A Nationwide Study. J Am Geriatr Soc **2014**, *62*, 1869–1876, doi:10.1111/jgs.13073.
479. Saad, M.; Matteucci, P. The Use of Electrochemotherapy (ECT) in the Management of Malignant Melanoma in the Clinically Frail Patient: A Case Report. Eur. J. Plast. Surg. **2020**, *43*, 855–858, doi:10.1007/s00238-020-01641-z.
480. Saad, T.F. Controversies in Management of Central Vein Stenosis Associated with Cardiac Rhythm Management Device (CRMD) Leads: There Are Times to Stent over That Lead! J. Vasc. Access **2010**, *11*, 233–234.
481. Saadat, L.V.; Mahvi, D.A.; Jolissaint, J.S.; Urman, R.D.; Gold, J.S.; Whang, E.E. Discharge Destination Following Rectal Cancer Resection: An Analysis of Preoperative and Intraoperative Factors. Int J Colorectal Dis **2020**, *35*, 249–257, doi:10.1007/s00384-019-03487-9.
482. Sadrzadeh, H.; Souidi, A. Acute Bleeding in an 80 Year Old Male: An Uncommon Diagnosis. J. Am. Geriatr. Soc. 2019, 67, S106, doi:10.1111/jgs.15898.
483. Saenz, H.; Motarjemi, R. Undiagnosed Secondary Adrenal Insufficiency a Life-Threatening Risk for Surgical Patients. J. Am. Geriatr. Soc. 2020, 68, S106, doi:10.1111/jgs.16431.
484. Saleem, S.; Shah, E.; Abbas, S.M.; Knight, C. From Pseudohypoglycemia to Diabetic Ketoacidosis. J. Gen. Intern. Med. 2019, 34, S538, doi:10.1007/11606.1525-1497.
485. Saltbæk, L.; Karlsen, R.V.; Bidstrup, P.E.; Høeg, B.L.; Zoffmann, V.; Horsbøl, T.A.; Holländer, N.H.; Svendsen, M.N.; Christensen, H.G.; Dalton, S.O.; et al. MyHealth: Specialist Nurse-Led Follow-up in Breast Cancer. A Randomized Controlled Trial–Development and Feasibility. Acta Oncol. **2019**, *58*, 619–626, doi:10.1080/0284186X.2018.1563717.
486. Schafer, S. Oral Cancer Screening in the Nursing Home. Alumni Bull Sch Dent Indiana Univ 1977, 35–36, 110.
487. Schenker, N.; Gentleman, J.F.; Rose, D.; Hing, E.; Shimizu, I.M. Combining Estimates from Complementary Surveys: A Case Study Using Prevalence Estimates from National Health Surveys of Households and Nursing Homes. Public Health Rep **2002**, *117*, 393–407, doi:10.1093/phr/117.4.393.
488. Schols, M.G.A. Sleep problems in the nursing home. *Tijdschr. Verpleeghuisgeneeskd.* **1998**, *22*, 3–6.
489. Schuster, T.G.; Hollingsworth, J.M.; Wei, J.T. Use of the "Decoy" Catheter to Prevent Traumatic Foley Removal. Clin. Geriatr. **2005**, *13*, 37–38.
490. Senan, S.; Haasbeek, C.J.; Antonisse, M.E.; Slotman, B.J.; Lagerwaard, F.J. Outcomes of Stereotactic Body Radiotherapy (SBRT) in 175 Patients with Stage I NSCLC Aged 75 Years and Older. J. Clin. Oncol. **2009**, *27*, 9545.
491. Seow, H.; Tanuseputro, P.; Barbera, L.; Earle, C.; Guthrie, D.; Isenberg, S.; Juergens, R.; Myers, J.; Brouwers, M.; Sutradhar, R. Development and Validation of a Prognostic Survival Model With Patient-Reported Outcomes for Patients With Cancer. JAMA Network Open 2020, 3, e201768–e201768, doi:10.1001/jamanetworkopen.2020.1768.
492. Sfeir, J.G.; Smith, K.M.; Surkis, W.D.; Tavani, D.M.; El-Hajj, R.J. Evaluation of Osteoporosis in Patients Admitted with Fragility Fractures at the Lankenau Medical Center. Endocr. Rev. 2016, 37, doi:10.1210/endo-meetings.2016.EHDE.1.SUN-768.
493. Shah, S.; Tartaro, C.; Chew, F.; Morris, M.; Wood, C.; Wuzzardo, A. Rehabilitation Efficiency and Effectiveness in Medical-Surgical Conditions: A Pilot Study of 234 Patients. Physical & Occupational Therapy in Geriatrics **2012**, *30*, 288–300, doi:10.3109/02703181.2012.731481.
494. Shankpal, P. Nurses for Cancer Prevention: Economics of Respiratory Cancer Projects. Supportive Care Cancer 2011, 19, S117–S118, doi:10.1007/s00520-011-1184-y.
495. Shapero, T.F.; Alexander, P.E.; Hoover, J.; Burgis, E.; Schabas, R. Colorectal Cancer Screening: Video-Reviewed Flexible Sigmoidoscopy by Nurse Endoscopist - A Canadian Community-Based Perspective. Can. J. Gastroenterol. **2001**, *15*, 441–445.
496. Sharif, S.; Oh, H. Is Autoimmunity the Common Factor amongst Chronic Inflammatory Demylinating Polyneuropathy, Amyotrophic Lateral Sclerosis and Psychiatric Disorders? J. Am. Geriatr. Soc. 2019, 67, S279, doi:10.1111/jgs.15898.

497. Sharp, L.; Foll, P.; Deady, S.; O'Ceilleachair, A.; Buicke, C.; Carsin, A.E. Where Do People with Lung Cancer Die and How Is This Changing? A Population-Based Study. Ir Med J **2010**, *103*, 262–264, 266.
498. Shaya, F.; Aljawadi, M.H.; Daniel Mullins, C.; Pandya, N.; Seal, B.; Hanna, N. Determinants of Trans-Arterial Chemoembolization (TACE) Treatments among Patients with Hepatocellular Carcinoma within Seermedicare. Hepatology 2011, 54, 594A, doi:10.1002/hep.24666.
499. Sherratt, A.; Skinn, E.; Gardiner, A. Gastrointestinal Cancers: An Update for Nurses. Nursing & Residential Care **2013**, *15*, 206–209, doi:10.12968/nrec.2013.15.4.206.
500. Sherwood PR; Given BA; Scholnik A; Given CW To Refer or Not to Refer: Factors That Affect Primary Care Provider Referral of Patients with Cancer to Clinical Treatment Trials. Journal of Cancer Education **2004**, *19*, 58–65, doi:10.1207/s15430154jce1901_13.
501. Shugarman, L.R.; Bird, C.E.; Schuster, C.R.; Lynn, J. Age and Gender Differences in Medicare Expenditures at the End of Life for Colorectal Cancer Decedents. J Womens Health (Larchmt) **2007**, *16*, 214–227, doi:10.1089/jwh.2006.0012.
502. Shuman, A.G.; Korc-Grodzicki, B.; Shklar, V.; Palmer, F.; Shah, J.P.; Patel, S.G. A New Care Paradigm in Geriatric Head and Neck Surgical Oncology. J Surg Oncol **2013**, *108*, 187–191, doi:10.1002/jso.23370.
503. Sieberth, E. [Report of 111 patients with multiple cancers of the first medical department of the Vienna-Lainz nursing home 1981-1987]. Wien Klin Wochenschr **1992**, *104*, 551–562.
504. Siegmund, L.A. Social Media in Occupational Health Nursing: Helpful or Harmful? Workplace Health Saf **2020**, *68*, 408–414, doi:10.1177/2165079920935779.
505. Sikorskii, A.; Given, C.W.; Siddiqi, A.-E.-A.; Champion, V.; McCorkle, R.; Spoelstra, S.L.; Given, B.A. Testing the Differential Effects of Symptom Management Interventions in Cancer. Psycho-Oncology **2015**, *24*, 25–32, doi:10.1002/pon.3555.
506. Silver, S.A.; Harel, Z.; McArthur, E.; Nash, D.M.; Acedillo, R.R.; Kitchlu, A.; Garg, A.X.; Chertow, G.M.; Wald, R. Causes of Death in Patients Who Survive a Hospitalization with AKI. J. Am. Soc. Nephrol. **2017**, *28*, 99.
507. Singh, A.; Braun, D.S. A Case of Patient with Stage vi Hepatocellular Carcinoma with Metastasis to the Spine Presenting with Acute Neurological Symptoms. Am. J. Gastroenterol. 2020, 115, S1405, doi:10.14309/01.ajg.0000712780.77839.9e.
508. Singh Joy SD Journal Watch. AJN American Journal of Nursing **2010**, *110*, 59–60.
509. Singh, S.; Eguchi, M.; Min, S.-J.; Fischer, S. Outcomes of Patients with Cancer Discharged to a Skilled Nursing Facility after Acute Care Hospitalization. JNCCN J. Nat. Compr. Cancer Netw. **2020**, *18*, 856–865, doi:10.6004/JNCCN.2020.7534.
510. Singh, S.R.K.; Malapati, S.; Kumar, R.; Lupak, O.; Kuriakose, P. Predictors of Transfer to Different Facility at Discharge in Patients Admitted with Hematologic Malignancy: A Nationwide Inpatient Sample (NIS) Database Analysis. Blood 2019, 134, doi:10.1182/blood-2019-127604.
511. Smith, C.B.; Bonomi, M.; Mhango, G.; Wisnivesky, J. Outcomes of Elderly Patients with Stage IIIB-IV Non-Small Cell Lung Cancer Admitted to the Intensive Care Unit. Am. J. Respir. Crit. Care Med. 2011, 183.
512. Smith MK Implementing Annual Cancer Screenings for Elderly Women. Journal of Gerontological Nursing 1995, 12–17.
513. Smith, P.M.; Reilly, K.R.; Miller, N.H.; DeBusk, R.F.; Taylor, C.B. Application of a Nurse-Managed Inpatient Smoking Cessation Program. Nicotine Tob. Res. **2002**, *4*, 211–222, doi:10.1080/14622200210123590.
514. Smith, S. Elderly See Themselves as in Good Health and Largely Independent. PUBLIC HEALTH REP. **1993**, *108*, 408–409.
515. Soliz, M.K.; Merai, P.G. A Millennial Caring for a Septuagenarian with Dementia. J. Am. Geriatr. Soc. 2018, 66, S180, doi:10.1111/jgs.15376.
516. Sperl-Hillen, J.M.; Anderson, J.P.; Haapala, J.; Chumba, L.; Ekstrom, H.; Appana, D.; Desilva, M.B.; O'connor, P.J. Better Care Strategies Are Necessary to Address Poor Medication Adherence. Diabetes 2020, 69, doi:10.2337/db20-1180-P.
517. Springer, S.; Mor, M.; Zhang, S.; Sileanu, F.; Zhao, X.; Aspinall, S.; Ersek, M.; Hanlon, J.; Hunnicutt, J.; Niznik, J.; et al. Prevalence and Factors Predicting Aspirin Use for Secondary Prevention in Veteran Affairs (VA) Nursing Home Residents with Limited Life Expectancy on Admission. J. Am. Geriatr. Soc. 2019, 67, S223, doi:10.1111/jgs.15898.
518. Staats DO Geriatric Gynecology in Long-Term Care Settings. Annals of Long Term Care **2000**, *8*, 52–58.
519. Stark, L.L.; Tofthagen, C.; Visovsky, C.; McMillan, S.C. The Symptom Experience of Patients with Cancer. Journal of Hospice & Palliative Nursing **2012**, *14*, 61–70, doi:10.1097/NJH.0b013e318236de5c.
520. Stein, W.M.; Min, Y.K. Nebulized Morphine for Paroxysmal Cough and Dyspnea in a Nursing Home Resident with Metastatic Cancer. Am J Hosp Palliat Care **1997**, *14*, 52–56, doi:10.1177/104990919701400201.
521. Suskind, A.M.; Zhao, S.; Boscardin, W.J.; Smith, A.; Finlayson, E. Time Spent Away from Home in the Year Following High-Risk Cancer Surgery in Older Adults. J Am Geriatr Soc **2020**, *68*, 505–510, doi:10.1111/jgs.16344.
522. Talley, K.M.C.; Wyman, J.F.; Savik, K.; Kane, R.L.; Mueller, C.E.; Hong Zhao Restorative Care's Effect on Activities of Daily Living Dependency in Long-Stay Nursing Home Residents. Gerontologist 2015, 55, S88–S98, doi:10.1093/geront/gnv011.
523. Tanoue, N.; Ummah, F.C.; Hanada, T.; Takajo, T.; Kamil, M.; Askoro, R.; Arita, K. Incidentally Found Primary Cerebral Malignant Melanoma Associated with Ota Nevus-Wide Dissemination after an Initial Phase of Slow Growth. Hiroshima J. Med. Sci. **2018**, *67*, 21–29.
524. Tathanhlong, L.; Bristow, B.; McGuffin, M. Providing Supportive Care for Young Breast Cancer Patients: Exploring the Comfort Level and Educational Needs of the Interprofessional Health Care Team at a Comprehensive Cancer Centre. J. Med. Imaging Radiat. Sci. 2015, 46, S69–S74, doi:10.1016/j.jmir.2015.04.015.

525. Taub, D.A.; Dunn, R.L.; Miller, D.C.; Wei, J.T.; Hollenbeck, B.K. Discharge Practice Patterns Following Cystectomy for Bladder Cancer: Evidence for the Shifting of the Burden of Care. J Urol **2006**, *176*, 2612–7; discussion 2617-2618, doi:10.1016/j.juro.2006.07.150.
526. Teggi, D. Care Homes as Hospices for the Prevalent Form of Dying: An Analysis of Long-Term Care Provision towards the End of Life in England. Social Science & Medicine 2020, 260, N.PAG-N.PAG, doi:10.1016/j.socscimed.2020.113150.
527. Tekin, N. An Example of Support Program to Elderly Patient and Elderly Relative during the Terminal Period in a Developed Model of Residential Home and Geriatric Care Center Gelişmiş Bir Dinlenme ve Bakimevi Modelinde Terminal Dönem Yaşli Olgu ve Yaşli Yakinina Destek Programi Örneği. Turk Geriatr. Derg. **2011**, *14*, 273–275.
528. Teno, J.M.; Kabumoto, G.; Wetle, T.; Roy, J.; Mor, V. Daily Pain That Was Excruciating at Some Time in the Previous Week: Prevalence, Characteristics, and Outcomes in Nursing Home Residents. J Am Geriatr Soc **2004**, *52*, 762–767, doi:10.1111/j.1532-5415.2004.52215.x.
529. Tharackal Ravindran, R.; Starr, R. Acute Generalized Exanthematous Pustulosis in an Elderly Female. J. Am. Geriatr. Soc. 2013, 61, S136, doi:10.1111/jgs.12263.
530. Thompson, C. A Job to Do. Nursing **1989**, *19*, 65, doi:10.1097/00152193-198912000-00028.
531. Thorpe, C.T.; Mor, M.; Zhang, S.; Sileanu, F.; Zhao, X.; Aspinall, S.; Ersek, M.; Hanlon, J.; Hunnicutt, J.; Niznik, J.; et al. Statin Discontinuation in Veterans Affairs (va) Nursing Home Residents with Limited Life Expectancy. *J. Am. Geriatr. Soc.* 2019, 67, S90, doi:10.1111/jgs.15898.
532. Thorpe, T.; Vilches-Moraga, A.; Fox, J.; Feilding, E.; Peeroo, M.K.; Wardle, K.; Alio, Z.R. Emergency Laparotomy in the Older Patient. Perioperative Care of Older People Undergoing Surgery-Salford General Surgery (POPS-SG). Eur. Geriatr. Med. 2015, 6, S151.
533. Tian G; Zhu Y; Qi L; Guo F; Xu H; Tian, G.; Zhu, Y.; Qi, L.; Guo, F.; Xu, H. Efficacy of Multifaceted Interventions in Reducing Complications of Peripherally Inserted Central Catheter in Adult Oncology Patients. Supportive Care in Cancer **2010**, *18*, 1293–1298, doi:10.1007/s00520-009-0747-7.
534. Topçu, B.; Vatan, V. Comparison of Endoscopic Diagnosis and Histopathological Results of Patients with a Preliminary Diagnosis of Malignancy and the Role of the Nurse in Planning. Prog. Nutr. 2020, 22, doi:10.23751/pn.v22i2-S.10171.
535. Traeger, L.; Pirl, W.F. Approaching the Treatment of Depression in Elderly Cancer Patients. Aging Health **2010**, *6*, 393–403, doi:10.2217/ahe.10.24.
536. Tramini, P.; Montal, S.; Valcarcel, J. Tooth Loss and Associated Factors in Long-Term Institutionalised Elderly Patients. Gerodontology **2007**, *24*, 196–203, doi:10.1111/j.1741-2358.2007.00183.x.
537. Triantos, D. Intra-Oral Findings and General Health Conditions among Institutionalized and Non-Institutionalized Elderly in Greece. J. Oral Pathol. Med. **2005**, *34*, 577–582, doi:10.1111/j.1600-0714.2005.00356.x.
538. Trtchounian, A.; Aberger, K.; Nelson, J. Early Palliative Care Consultation Associated with Decreased Length of Stay. Ann. Emerg. Med. 2017, 70, S115.
539. Trujillo Vílchez, R.; Morgado Bermejo, N.; Pozo Villa, R.; Lapeira Cabello, J.M.; Gómez Thébaut, A.; Rosúa Rodríguez, M.; Villalba Merchán, O.A.; Romero Montero, E.; Ortega Morell, A.; De La Ossa Sendra, M.J.; et al. Experience with the CUDECA Foundation palliative care program. Med. Paliativa **2007**, *14*, 217–221.
540. Tschopp, J.M.; Brutsche, M.; Frey, J.G.; Tatti, B.; Aymon, E.; Spiliopoulos, A. [Thoracic surgery for non-small cell lung cancer. Cost-benefit of its management in specialized intermediate care]. Swiss Surg Suppl 1996, Suppl 1, 46–51.
541. Tseng, O.; Spinelli, J.; Gotay, C.; McBride, M.; Dawes, M. Utilization of Bone Mineral Density Testing among Breast Cancer Survivors in British Columbia, Canada. Osteoporosis International **2017**, *28*, 3439–3449, doi:10.1007/s00198-017-4218-6.
542. Tseng, Y.-P.; Huang, L.-H.; Huang, T.-H.; Hsu, L.-L.; Hsieh, S.-I. Factors Associated With the Do-Not-Resuscitate Decision Among Surrogates of Elderly Residents at a Nursing Home in Taiwan. Int. J. Gerontol. **2017**, *11*, 95–99, doi:10.1016/j.ijge.2016.07.004.
543. Tur-Sinai, A.; Urban, D.; Bentur, N. Out-of-pocket Spending of Deceased Cancer Patients in Five European Countries and Israel. European Journal of Cancer Care **2021**, *30*, 1–6, doi:10.1111/ecc.13372.
544. Ugbarugba, E.E.; Stanfill, C.; Aninyei, E. A Case of Hepatic Inflammatory Pseudotumor. Am. J. Gastroenterol. 2017, 112, S1189, doi:10.1038/ajg.2017.321.
545. Ursem, C.J.; Diaz-Ramirez, L.G.; Lang-Brown, S.; Wei, X.X.; Chen, R.C.; Lee, S. Association of Changes in Functional Status with Radiation for Prostate Cancer in Older Veterans. J. Clin. Oncol. 2018, 36, doi:10.1200/JCO.2018.36.6_suppl.148.
546. van Ballegooijen, M.; Koopmanschap, M.A.; Tjokrowardojo, A.J.; van Oortmarssen, G.J. Care and Costs for Advanced Cervical Cancer. Eur. J. Cancer 1992, 28A, 1703–1708, doi:10.1016/0959-8049(92)90072-a.
547. Van Der Heide, A.; Rietjens, J.; Onwuteaka-Philipsen, B.; Van Delden, J. Developments in the Practice of Palliative Sedation in The Netherlands (2005-2015). Palliative Med. **2018**, *32*, 26, doi:10.1177/0269216318769196.
548. van der Plas, A.G.M.; Oosterveld-Vlug, M.G.; Pasman, H.R.W.; Onwuteaka-Philipsen, B.D. Relating Cause of Death with Place of Care and Healthcare Costs in the Last Year of Life for Patients Who Died from Cancer, Chronic Obstructive Pulmonary Disease, Heart Failure and Dementia: A Descriptive Study Using Registry Data. Palliative Medicine **2017**, *31*, 338–345, doi:10.1177/0269216316685029.
549. Van Rij, S.; Andrews, A.W.; Freund, J.; Bailey, S. Multimodal Rehabilitation Following Gliosarcoma Resection: A Case Report. Rehabil. Oncol. **2021**, *39*, 56–63, doi:10/9701.REO.0000000000000206.

550. Vetto JT; Petty JK; Dunn N; Prouser NC; Austin DF Structured Clinical Breast Examination (CBE) Training Results in Objective Improvement in CBE Skills. Journal of Cancer Education **2002**, *17*, 124–127.
551. Vetto JT; Prouser NC; Flemming DW; Hedberg KM Assessing the Need for Clinical Breast Examination (CBE) Training: The Oregon Experience. Journal of Cancer Education **2000**, *15*, 209–213.
552. Viele, C.S. Diagnosis, Treatment, and Nursing Care of Acute Leukemia. Semin Oncol Nurs **2003**, *19*, 98–108.
553. Vilches-Moraga, A.; Fox, J.; Paracha, A.; Gomez-Quintanilla, A.; Maevis, T.; Epstein, J.; Thomson, A.M. Baseline Characteristics and Clinical Outcomes of Older Patients Admitted as an Emergency to General Surgical Wards. Salford-POPS – GS. Eur. Geriatr. Med. **2017**, *8*, 499–504, doi:10.1016/j.eurger.2017.07.012.
554. Vondeling, A.; Hazen, J.; Van Huis Tanja, L.; Schiphorst, A.; Dekker, J.; Hamaker, M. FACTORS ASSOCIATED WITH SURVIVAL IN ELDERLY PATIENTS WITH STAGE I–III COLORECTAL CARCINOMA IN THE NETHERLANDS MANAGED WITH NON-CURATIVE INTENT. J. Geriatr. Oncol. 2019, 10, S31–S32, doi:10.1016/S1879-4068(19)31168-3.
555. Wachtel, T.J.; Derby, C.; Fulton, J.P. Predicting the Outcome of Hospitalization for Elderly Persons: Home versus Nursing Home. South. Med. J. **1984**, *77*, 1283–1285, 1290, doi:10.1097/00007611-198410000-00020.
556. Wagg, A.; Staskin, D.; Engel, E.; Herschorn, S.; Kristy, R.M.; Schermer, C.R. A Phase 4, Double-Blind, Randomized, Placebo-Controlled, Parallel Group, Multi-Centre Study to Evaluate the Efficacy, Safety, and Tolerability of Mirabegron in Older Adult Patients with Overactive Bladder Syndrome (Pillar). Neurourol. Urodyn. 2018, 37, S215–S216.
557. Wahler, R.G.; Reiman, A.T.; Schrader, J.V. Use of Compounded Dextromethorphan-Quinidine Suspension for Pseudobulbar Affect in Hospice Patients. Journal of Palliative Medicine **2017**, *20*, 294–297, doi:10.1089/jpm.2016.0277.
558. Walling, A.M.; D'Ambruoso, S.F.; Malin, J.L.; Hurvitz, S.; Zisser, A.; Coscarelli, A.; Clarke, R.; Hackbarth, A.; Pietras, C.; Watts, F.; et al. Effect and Efficiency of an Embedded Palliative Care Nurse Practitioner in an Oncology Clinic. J. Oncol. Pract. 2017, 13, e792–e799, doi:10.1200/JOP.2017.020990.
559. Walter, J.; Tufman, A.; Schwarzkopf, L. Are Elderly Lung Cancer Patients Treated Differently? Results from a German Claims Data Analysis. Pneumologie 2018, 72, doi:10.1055/s-0037-1619215.
560. Wang, B.; Hylwka, T.; Smieja, M.; Surrette, M.; Bowdish, D.M.E.; Loeb, M. Probiotics to Prevent Respiratory Infections in Nursing Homes: A Pilot Randomized Controlled Trial. Journal of the American Geriatrics Society **2018**, *66*, 1346–1352, doi:10.1111/jgs.15396.
561. Wang, H.; Qiu, F.; Boilesen, E.; Nayar, P.; Lander, L.; Watkins, K.; Watanabe-Galloway, S. Rural-Urban Differences in Costs of End-of-Life Care for Elderly Cancer Patients in the United States. Journal of Rural Health **2016**, *32*, 353–362, doi:10.1111/jrh.12160.
562. Ward-Smith P Abstracts. Urologic Nursing **2009**, *29*, 264–266.
563. Warren, G.; Shah, N.; Crossley, H.; Dailey, S.; Hafez, K.; He, C.; Tallman, C.; Montgomery, J.; Weizer, A.; Lee, C. Hospital Discharge Status of Elderly Patients Treated with Radical Cystectomy for Bladder Cancer. J. Urol. 2013, 189, e724, doi:10.1016/j.juro.2013.02.2891.
564. Wergeland Sørbye, L.; Steindal, S.A.; Kalfoss, M.H.; Vibe, O.E. Opioids, Pain Management, and Palliative Care in a Norwegian Nursing Home From 2013 to 2018. Health Services Insights 2019, 12, N.PAG-N.PAG, doi:10.1177/1178632919834318.
565. Weyer, C.; Zeitouni, N.C.; Jackson, J.R. Three-Staged Reconstruction of a Nasal Defect. Dermatol. Surg. **2018**, *44*, 1611–1614, doi:10.1097/DSS.0000000000001487.
566. Wick, J.Y.; Zanni, G.R. Aging in Place: Multiple Options, Multiple Choices. Consult Pharm **2009**, *24*, 804–806, 808, 811–812, doi:10.4140/tcp.n.2009.804.
567. Wijnia JW; Corstiaensen IJP A Poor Prognosis: Guide or Misleading? American Journal of Hospice & Palliative Medicine **2008**, *25*, 5–8, doi:10.1177/1049909107307379.
568. Wildiers, H.; Mauer, M.; Elseviers, M.; De Wolf, J.; Hatse, S.; Hamaker, M.; Buntinx, F.; De Lepeleire, J.; Uytterschaut, G.; Falandry, C.; et al. Cancer Events in Belgian Nursing Home Residents: An EORTC Prospective Cohort Study. J Geriatr Oncol **2019**, *10*, 805–810, doi:10.1016/j.jgo.2019.03.005.
569. Williams, G.R.; Dunham, L.; Chang, Y.; Deal, A.M.; Pergolotti, M.; Lund, J.L.; Guerard, E.J.; Kenzik, K.; Muss, H.B.; Sanoff, H.K. Geriatric Assessment to Predict Hospitalization Frequency and Long-Term Care Utilization in Older Adult Cancer Survivors. J. Clin. Oncol. 2018, 36, doi:10.1200/JCO.2018.36.15-suppl.10036.
570. Wilson, D.M.; Truman, C.D. Research Note: Describing Canada's Residential Continuing Care Population through an Analysis of a National Mortality Dataset and a Provincial Hospital Dataset. Can. J. Aging **2003**, *22*, 127–131, doi:10.1017/S0714980800003780.
571. Wilson, K.B.; Ardoin, K.B. When Professional and Personal Worlds Meet: Nurse as Daughter. MEDSURG Nursing **2013**, *22*, 192–196.
572. Wu, J.H.; Guo, Z.; Berman, R.; Coric, V.; Kumar, S.; Radhakrishnan, M. Risk of Non-Melanoma Skin Cancer in Elderly Patients with Alzheimer's Disease. Pharmacoepidemiol. Drug Saf. 2011, 20, S303–S304, doi:10.1002/pds.2206.
573. Wu, J.; Mafham, M.; Mamas, M.A.; Rashid, M.; Kontopantelis, E.; Deanfield, J.E.; de Belder, M.A.; Gale, C.P. Place and Underlying Cause of Death During the COVID-19 Pandemic: Retrospective Cohort Study of 3.5 Million Deaths in England and Wales, 2014 to 2020. Mayo Clin Proc **2021**, *96*, 952–963, doi:10.1016/j.mayocp.2021.02.007.
574. Wu, J.; Quill, T. Geriatric Rehabilitation and Palliative Care. Topics in Geriatric Rehabilitation **2011**, *27*, 29–35, doi:10.1097/TGR.0b013e3181ff6844.

575. Wu, J.; Quill, T. Geriatric Rehabilitation and Palliative Care. Topics in Geriatric Rehabilitation **2011**, *27*, 29–35, doi:10.1097/TGR.Ob013e3181ff6844.
576. Xiong, B.; Freeman, S.; Banner, D.; Spirgiene, L. Hospice Use and One-Year Survivorship of Residents in Long-Term Care Facilities in Canada: A Cohort Study. BMC Palliative Care 2019, 18, N.PAG-N.PAG, doi:10.1186/s12904-019-0480-z.
577. Yadav, N.R.; Jain, M.; Sharma, A.; Yadav, R.; Pahuja, M.; Jain, V. Distribution and Prevalence of Oral Mucosal Lesions in Residents of Old Age Homes in Delhi, India. Nepal J. Epidemiol. **2018**, *8*, 727–734, doi:10.3126/nje.v8i2.18708.
578. Yang, A.; Lall, C.; Bhargava, P.; Imagawa, D. Limited Utility of Plain Abdominal Radiographs in Evaluating Intussusceptions Secondary to Long Indwelling Feeding Tubes. J. Clin. Imaging Sci. 2013, 3, doi:10.4103/2156-7514.107994.
579. Yong, C.; Onukwugha, E.; Mullins, C.D.; Seal, B.; Hussain, A. The Use of Health Services among Elderly Patients with Stage IV Prostate Cancer in the Initial Period Following Diagnosis. J Geriatr Oncol **2014**, *5*, 290–298, doi:10.1016/j.jgo.2014.04.001.
580. Zanni, G.R.; Wick, J.Y.; Walker, B.L. Sexual Health and the Elderly. Consult. Pharm. **2003**, *18*, 310–322.
581. Zavaroni, C. Home Care and Short-Run Nursing Homes: Organizational Aspects of Their Integration with Oncological Organizations. Crit. Rev. Oncol. Hematol. **2001**, *39*, 247–267, doi:10.1016/s1040-8428(00)00118-9.
582. Zenimoto, M.; Kita, M.; Arai, T.; Murayama, T.; Mori, M. [Free/total prostate specific antigen ratio (%PSA) to predict probability of prostate cancer]. Rinsho Byori **2003**, *51*, 969–973.
583. Zweig, S. Bert's Dad. J. Am. Med. Dir. Assoc. **2002**, *3*, 278–279, doi:10.1016/S1525-8610(04)70495-8.
584. Hospital Services for Selected Chronic Disease Patients. ABSTR. HOSP. MANAGE. STUD. **1974**, *11*, 11965 CH:706p.
585. Optimal Criteria for Care of Patients with Cancer. JAMA **1974**, *227*, 57–63.
586. Case Presentation 2 (1990): The Unsafe Hospital Discharge -- Responses by the Hartford Hospital's HEC and the Patient's Hospital's HEC. HEC Forum **1990**, *2*, 279–284.
587. [Methods and instruments of nursing research: An analysis of some articles]. Riv Inferm **1993**, *12*, 29–32.
588. '97 TNA House Resolutions. Texas Nursing **1997**, *71*, 7–10.
589. Pain in Nursing-Home Cancer Patients Often Goes Untreated. Am J Health Syst Pharm **1998**, *55*, 1544, 1548, doi:10.1093/ajhp/55.15.1544.
590. Position of the American Dietetic Association: Oral Health and Nutrition. Journal of the American Dietetic Association **2003**, *103*, 615–625, doi:10.1053/jada.2003.50130.
591. Web Watch. Case Manager **2004**, *15*, 20–21.
592. EONS News 9.3. European Journal of Oncology Nursing **2005**, *9*, 199–201.
593. In Brief. BMJ: British Medical Journal (International Edition) **2006**, *332*, 928–928, doi:10.1136/bmj.332.7547.928.
594. Discussions. Ann. Surg. **2008**, *248*, 465–467, doi:10.1097/SLA.0b013e318185e1b3.
595. When the System Fails an Elderly Patient. Ann. Long-Term Care **2008**, *16*, 12–13.
596. 2008 SNRS Abstracts -- B. Southern Online Journal of Nursing Research **2008**, *8*, 2–2.
597. 2008 SNRS Abstracts -- I - K. Southern Online Journal of Nursing Research **2008**, *8*, 7–7.
598. 2008 SNRS Abstracts -- N - Q. Southern Online Journal of Nursing Research **2008**, *8*, 10–10.
599. Abstracts of the 2011 Joanna Briggs Institute International Convention...7–9 November 2011, National Wine Centre, Adelaide, South Australia. International Journal of Evidence-Based Healthcare **2011**, *9*, 282–334, doi:10.1111/j.1744-1609.2011.00182.x.
600. Cancer Often Goes Untreated in Nursing Home Residents. Community Oncology **2011**, *8*, 557–557.
601. MINERVA. BMJ: British Medical Journal (Overseas & Retired Doctors Edition) **2012**, *345*, 50–50.
602. Looking for More Meal Appeal. Nursing **2012**, *42*, 10–10.
603. 9th Congress of the EUGMS. Eur. Geriatr. Med. 2013, 4.
604. Singapore Health and Biomedical Congress, SHBC 2013. Ann. Acad. Med. Singapore 2013, 42, S1.
605. Poster Abstracts - Post Acute and Long Term Care Medicine 2018. J. Am. Med. Informatics Assoc. 2018, 19.

Table S4. Characteristics of studies, investigation.

Authors - Year	Population	Object	Setting	Design	Inclusion Criteria	Interventio n	Main Outcome Measures	Results	Principal Source of Bias
Koroukian et al. – 2007 [1]	4,404 breast cancer patients, 5,334 prostate cancer patients, and 4,822 colorectal cancer patients	Complexity of Care Needs and Unstaged Cancer in Elders: A Population-Based Study	Nursing homes, Ohio, US	Cross-sectional population-based study	All 65 years or older diagnosed with breast, prostate or colorectal cancer, during the year 2000	Info from databases OCISS, OASIS, MDS.	Occurrence of unstaged cancer and complexity of care needs (COCN)	Proportion of unstaged cases was 7.6% among breast, 16.9% for prostate, and 13.8% for colorectal cancer	Selection

								patients. Older age groups compared to younger had higher proportion of unstaged cases. Compared with patients with low complexity of care needs, those with higher complexity were 4 to 5 times as likely to have unstaged cancer.	
Hamaker et al.-2012 [2]	1239 of elderly care physicians registered at the National Association of Elderly Care Physicians and social Geriatricians	Nonreferral of Nursing Home Patients with Suspected Breast Cancer	Primary care setting, Netherland	Web-based survey	All members of the National Association of Elderly Care Physicians and Socials Geriatricians in the Netherland	Web-based survey	The extent of non-referral of patients suspected of breast cancer by ECP and the motivations behind this choice.	Thirty-three percent 33% of elderly care physicians reported not referring the last patient. Motivations were end-stage dementia in 57%, patient/family preference in 29% and limited life expectancy (23%). 16% of non-referred patients had hormonal treatment started without diagnostic confirmation of cancer. Motivations to refer	Volunteer

								patients were to confirm the diagnosis (28%), fear of future ulceration or metastases (21%), good general health and life expectancy (19%), and the desire of the patient's or families to investigate (18%).	
Christos et al. – 2004 [3]	457 nurses form the Texas State Board of Nurse Examiners	Skin cancer prevention and detection by nurses: attitudes, perceptions, and barriers.	Public health, Texas, US	Descriptive survey	Full-time, employed, public health and oncology nurses	The survey instrument was a 4-page questionnaire	Knowledge of skin cancer as a public health problem, the perceived importance of and opportunity for skin cancer prevention and detection, and potential barriers to skin cancer prevention and detection services by nurse	Eighty-four percent stated that it was in their scope of practice to teach skin cancer prevention to patients, and 61%, to detect skin cancer in patients. Barriers to continuing education for skin cancer prevention and detection included lack of financial resources (43%), not knowing educational opportunities (41%), time away from work (36%), and time away	Sampling

Bradley et al. – 2008 [4]	1907 elderly NH residents with cancer	Absence of Cancer Diagnosis and Treatment in Elderly Medicaid-Insured Nursing Home Residents	Nursing homes, Michigan, US	Populational study	Nursing home residents diagnosed with cancer between January 1, 1997, and December 31, 2000, who have at least 1 year of claims history before the month of diagnosis to estimate patients' comorbidity burden	Dataset assembled from the Michigan Tumor Registry and Medicare records	Cancer site for late or unstaged, cancer at diagnosis, death within 3 months of diagnosis, receipt of hospice care, and the likelihood of cancer-directed surgery.	from home (26%). Preponderance of late or unstaged disease (62%), high mortality within 3 months of diagnosis (48%), and low acute care facilities use if they were metastatic (28%). Twenty-two percent received cancer-directed surgery, 61% was for breast cancer patients. Patients who went under intervention were younger (OR = 2.83, 95%CI [1.26; 6.32]; RR = 1.37, 95%CI [1.08; 1.75]). Only 6% of patients received a systemic treatment, via chemotherapy and/or radiation.	Selection
Yadav et al. – 2018 [5]	464 NH residents	Distribution and prevalence of oral mucosal lesions in residents of old age	Old age homes in Delhi, India	Cross sectional study	65-74-year-old, in old age homes	Survey was prepared based on Oral Health Assessment Form, WHO Clinical examination was	Distribution and prevalence of oral mucosal lesions	Frequent oral mucosal lesions reported were malignant tumors, leukoplakia,	Selection

		homes in Delhi, India				performed using two mouth mirrors under natural illumination		lichen planus, ulcerations, ANUG, abscess and candidiasis. Leukoplakia was seen in 15% of residents. Malignant tumors were seen in 1,5% and mostly on the floor of mouth.	
Clement et al. – 2009 [6]	1316 NH residents without a cancer diagnosis but developed the disease during their stay	Organizational characteristics and cancer care for nursing home residents	Nursing homes, Michigan, US	Cohort study	Subsequently diagnosed with cancer from 1997 to 2000, Residents for at least 31 days	Data from the Michigan Tumor Registry (1997–2000), Medicare claims, Medicaid cost reports, and the Area Resource File	Timeliness of diagnosis, defined as diagnosis at death or in the same month as death, is a care outcome. Pain medication administration during the month of diagnosis or the month following diagnosis.	Twenty-five percent were diagnosed at death or during the month they died. Seventy-four percent of diagnosis were made at late or unstaged cancer. Risk factors were oldest and Alzheimer disease.	Selection
Rudel et al. – 2007 [7]	39 directors of nursing for rural long-term care facilities	On breast cancer detection, directors of nursing and female residents: a study in rural long-term care.	Rural long-term care facilities, Midwestern state, US	Descriptive study	Long term care facilities situated in communities with a population of under 2500	Survey with fixed-choices, Likert-scales, and qualitative open-ended questions	Clinical breast exam frequency, mammography frequency, factors influencing screening services	Twenty-six percent of directors of nursing of LTC facilities reported that over 75% of the elderly women do receive regular CBEs by a provider, 43.6% respondents reported that less	Selection

								than 25% receive a CBE. The perceived barriers to screening were facility physician(s) do not encourage mammograms; and some believe that mammograms are too expensive to be efficacious in LTC. They reported that embarrassment for elderly women residents and time for CBE and mammography would not be an issue.	
Wildiers et al. – 2019 [8]	4262 NH residents	Cancer events in Belgian nursing home residents: An EORTC prospective cohort study	Nursing homes, Belgium	Prospective cohort study	Being a resident in one of the 39 nursing home during the study period	Data from a questionnaire completed by the dedicated nurse and the treating general practitioner in the nursing home. Medical documents from hospitalization/specialist consultation were also used.	Incidence rate of cancer	In 9 residents, a cancer event was recorded during follow-up: 5 new (suspected or diagnosed) cancer events (incidence rate = 123/100.000 NHR per year) and 4 with (suspected or diagnosed) progressive	Reporting

Darjani et al. – 2020 [9]	259 nursing home residents	Skin Diseases among the Old Age Residents in a Nursing Home: A Neglected Problem	Charity nursing home, Iran	Cross-sectional study	All residents more than 60 years form November 2017 to March 2018	Full-body skin examination by dermatologists, confirmation of the disease by biopsy, pathological and laboratory methods	Frequency of dermatological diseases	disease. No diagnostic procedure was performed in 4 residents suspected with cancer, and in 5 no anticancer treatment was started. Almost 86% had age-related skin changes. The benign neoplasm was the most common skin disease among patients (68.3%), followed by infectious diseases (46.3%) and erythema-squamous (31.6%). None of them had precancerous lesions or skin cancers.	Selection

Table S5. Characteristics of studies, treatment.

Authors - Year	Population	Object	Setting	Design	Inclusion Criteria	Intervention	Main Outcome Measures	Results	Principal Source of Bias
Hyman et al. – 2005 [10]	6487 women with lymph node-positive breast cancer	Adjuvant Chemotherapy in Older and Younger Women with Lymph Node–Positive Breast Cancer	Academic and community medical centers, US	Retrospective review of data from 4 randomized trials	From 4 CALGB randomized trials, designed for patients with node-positive breast cancer	Comparison of at least 2 chemotherapy regimens that differed by dose level, dose intensity, or regimen	Comparison of disease-free survival, overall survival, and treatment-related mortality among	Increased odds of death from treatment toxicity (33 deaths, 0.5% of all patients). Adjuvant chemotherapy and	Selection

							different age groups	tamoxifen improved significatively disease-free and overall survival in older women with node-positive disease. No association between age, disease-free survival and reduction in breast cancer mortality. Death from causes other than breast cancer deteriorates overall survival (P<.001) for patients aged 65 or older.	
Liuu et al. – 2019 [11]	214 NH residents with cancer	Prevalence of cancer and management in elderly nursing home residents. A descriptive study in 45 French nursing homes	45 nursing homes, France	Descriptive retrospective study	All residents with cancer or recurrence of cancer occurring after the age of 75 years regardless of tumoral site and oncologic treatment plan	Data from the medical records of NH, local hospital and loco-regional online cancer registry	Cancer prevalence occurring after the age of 75, residents' characteristics and parameters associated with cancer specific management.	Most common tumoral sites were skin, digestive tract and breast. The residents were moderately dependent based on the AGGIR scale in 50% of cases and highly dependent in 43%. Staging information was available in 61%, and reports of a multidisciplinary discussion	Volunteer

								35%. Surgery was the most frequently treatment performed (55%). 17% patients were still undergoing cancer treatment. Decision of cancer-specific treatment was significantly lower (69% vs. 86%, p = 0.013) in residents with cancer diagnosed since admission in nursing home compared to those with a diagnosis before admission. Palliative management was frequent in residents with cancer diagnosed in NH.	
Mercedes et al. – 2019 [12]	24 family members of NH residents with cancer	Prospect Theory Concepts Applied to Family Members of Nursing Home Residents With Cancer: A Good Ending Is a Gain	4 nursing homes in a college town in a mid-western state, US	Secondary analysis of qualitative interview data	Family members of current or recent NH residents with cancer diagnosis	Interview	Prospect theory concepts of gain, loss, risk and reference point were used in qualitative content analysis.	Three themes comprise the main findings, including "Don't prolong this", "A good ending is a gain" and experience can facilitate seeing the big picture"	Sampling

Tang et al.-2018 [13]	5969 NH residents who underwent breast cancer surgery.	Functional Status and Survival After Breast Cancer Surgery in Nursing Home Residents	All Medicare nursing homes, US	Cohort study	Female long-term nursing home residents (age ≥67 years) who underwent an inpatient breast cancer surgical procedure by linking data from 100% national Medicare inpatient files (2003-2013) with the Minimum Data Set (MDS) for nursing homes (2003-2013).	Medicare claims from 2003 to 2013, use of Minimum Data Set Activities of Daily Living (MDS-ADL) summary score	Functional status and death.	Of 5969 female NH residents who underwent surgery (mean [SD] age, 82 [7] years) 666 (11.2%) underwent lumpectomy, 1642 (27.5%) underwent mastectomy, and 3661 (61.3%) underwent lumpectomy or mastectomy with axillary lymph node dissection (ALND). The 30-day mortality rates were 8% after lumpectomy, 4%after mastectomy, and 2%after ALND. The 1-year mortality rates were 41% after lumpectomy, 30% after mastectomy, and 29% after ALND. Among 1-year survivors, the functional decline rate was 56% to 60%. The mean MDS-ADL score increased by 3 points for lumpectomy	Information

, 4 points for mastectomy, and 5 points for ALND. In multivariate analysis, poor baseline MDS-ADL score was associated with a higher 1-year mortality risk (lumpectomy: HR, 1.92, 95%CI [1.23;3.00], P = .004; mastectomy: HR, 1.80, 95%CI [1.35;2.39], P < .001; and ALND: HR, 1.77, 95%CI [1.46;2.15], P < .001). After multivariate adjustment, preoperative decline in MDS-ADL score (lumpectomy: sHR, 1.59, 95%CI [1.25;2.03], P < .001; mastectomy: sHR, 1.79, 95%CI [1.52;2.09], P < .001; and ALND: sHR, 1.72, 95%CI [1.56;1.91], P < .001) and cognitive impairment (lumpectomy: sHR, 1.27, 95%CI [1.03;1.56], P

								= .02; mastectomy: sHR, 1.26, 95%CI [1.09;1.45], P = .002; and ALND: sHR, 1.14, 95%CI [1.04;1.24], P = .003) were significantly associated with 1-year functional decline across all breast cancer surgery groups.	
Finlayson E et al. – 2012 [14]	6822 NH residents who underwent surgery for colon cancer	Functional Status After Colon Cancer Surgery in Elderly Nursing Home Residents	Nursing homes, US	Retrospective cohort study	Nursing home residents, 65 and older, underwent surgery for colon cancer between 1999 and 2005. Classified as long-term nursing home residents if they underwent two or more consecutive MDS assessments more than 90 days apart during the 6 months before surgery.	Data from Medicare Inpatient Files (2000–2005) linked with those from the Minimum Data Set for Nursing Homes. Identification with International Classification of Diseases, Ninth Revision (ICD-9) diagnosis and procedure codes. Comparison with cohort of nursing home residents who did not undergo surgery during the same period matched on age, gender, race, year of admission,	Functional status defined with MDS-ADL and mortality	Functional decline rate of 24%, for a 3.9-point worsening in MDS-ADL score at 1 year. Factors associated were older age (>80 vs 65–69, adjusted relative risk (ARR) = 1.53, 95%CI [1.15;2.04], P < .001), hospital readmission after surgical hospitalization (ARR = 1.15, 95%CI [1.03;1.29], P = .02), surgical complications (ARR = 1.11, 95%CI [1.02;1.21], P = .01), and functional decline before surgery (ARR = 1.21, 95%CI	Information

						and comorbidity.		[1.11;1.32], P < .001).	
Finlayson et al.- 2012 [15]	979 NH residents undergoing proctectomy for rectal cancer	Outcomes After Rectal Cancer Surgery in Elderly Nursing Home Residents	Nursing homes, US	Retrospective cohort study	Long-term nursing home residents underwent 2 or more consecutive MDS assessments performed over 90 days apart during the 6 months before surgery, undergoing oncologic resection for rectal cancer with proctectomy with permanent colostomy and sphincter-sparing proctectomy (2000–2005)	Data from national Medicare Inpatient Files (2000–2005) with the Minimum Data Set for Nursing Homes (MDS, 1999–2006).	Fecal incontinence and the 1-year mortality rate	Operative mortality was 18% after proctectomy with permanent colostomy and 13% after sphincter-sparing proctectomy (adjusted relative risk, 1.25, 95%CI [0.90;1.73], p = 0.188) and one-year mortality was also high with respectively 51% and 40% (adjusted hazard ratio 1.32, 95%CI [1.09;1.60], p = 0.004). After sphincter-sparing proctectomy, 37% of residents were incontinent of feces.	Selection
Chan et al. – 2019 [16]	57 patients aged 90 or above	Outcomes of colorectal cancer surgery in the nonagenarians: 20-year result from a tertiary center	Tertiary center, Hong Kong, China	Retrospective observational study	Patients aged 90 or above, underwent operations all patients who suffered from histologically proven adenocarcinoma of the colon and rectum and underwent	Review of medical records	Complications rate, 30-day and 180-day mortality rates	Surgery with curative intent (77.2%), elective setting (57.9%) and open approach (78.9%). Postoperative complications happened in 36.8% of patients,	Selection

					surgery between January 1996 and December 2015			postoperative pneumonia was the most common. The 30-day and 180-day mortality rate was 7 and 31.6% respectively. The 1 and 2-year survival rate for elective surgery was 69.7 and 54.5% respectively.	
Bradley et al. – 2008 [4]	1907 elderly NH residents with cancer	Absence of Cancer Diagnosis and Treatment in Elderly Medicaid-Insured Nursing Home Residents	Nursing homes, Michigan, US	Populational study	Nursing home residents diagnosed with cancer between January 1, 1997, and December 31, 2000, who have at least 1 year of claims history before the month of diagnosis to estimate patients' comorbidity burden	Dataset assembled from the Michigan Tumor Registry and Medicare records	Cancer site for late or unstaged, cancer at diagnosis, death within 3 months of diagnosis, receipt of hospice care, and the likelihood of cancer-directed surgery.	Preponderance of late or unstaged disease (62%), high mortality within 3 months of diagnosis (48%), and low hospice use if they had distant-stage cancer (28%). Only 22% received cancer-directed surgery, 61% of which was breast cancer patients, and 6% received chemotherapy and/or radiation. Older age was positively associated with late or unstaged cancer and with death within 3 months of	Selection

								diagnosis. Patients aged 71 – 75 years were more likely to have cancer-directed surgery than patients aged 86 years and older (OR = 2.83, 95%CI [1.26;6.32]; RR = 1.37, 95%CI [1.08;1.75].	
Williams et al. – 2018 [17]	125 older adults	Geriatric assessment to predict hospitalization frequency and long-term care utilization in older adult cancer survivors	North Carolina Cancer Hospital, US	Cohort study	Age 65 years and over, a cancer diagnosis, completed the GA from 3 months pre-diagnosis through 6 months postdiagnosis, have continuous enrollment in both Medicare Parts A and B, with no enrollment in managed care plans for 6 months before and after cancer diagnosis	Data collected from Medicare claims	Hospitalizations and long-term care utilization, geriatric assessment risk measures (frail status, impaired IADL, limitations in climbing stairs, falls, TUG, unintended weight lost)	Compared to their counterparts living at home, factors associated with long term care use were prefrail/frail status (RR, 1.86; P , .007), instrumental activities of daily living impairment (RR, 4.58; P , .001), history of falls (RR, 6.73; P , .001), prolonged Timed Up and Go (RR, 5.45; P , .001), and limitations in climbing stairs (RR, 1.89; P , .005).	Sample
Buchanan et al. – 2005 [18]	548 330 patients admitted in NH	Analyses of Nursing Home Residents with Cancer	Nursing home, US	Descriptive study	Nursing home admission assessments recorded in the national	Data from MDS	Sociodemographic characteristics, measure of health status, levels	11% of all admissions was diagnosed cancer. Majority was	Information

		at Admission				Minimum Data Set (MDS) during 2002	of physical dependency, cognitive performance, and treatments or therapies received.	dependent for activities of daily living and more than a half used a wheelchair. More than 37% experienced at least moderate daily pain at admission and require heavy care; 26% had a diagnosis of depression. More than 50% of residents with cancer had an unstable health condition, 21% had a life expectancy of less than six months, and 19% received hospice care. More than 40% of residents with cancer had no advance directives recorded at admission	
Blytt et al. – 2018 [19]	1825 NH patients with and without dementia and cancer	Comorbid Dementia and Cancer in Residents of Nursing Homes: Secondary Analyses of a Cross-Sectional Study	64 nursing homes, located in urban and rural areas in 5 of 19 counties of Norway	Cross-sectional study	All patients who had lived in the NH for at least 2 weeks	Medical record, standardized interviews with the patient's primary caregiver	Prevalence of cancer among NH patients with and without dementia and compare patients with comorbid dementia and cancer with those without, the	84 % had dementia, and 5.5% had comorbid dementia and cancer. Patients with comorbid dementia and cancer received significantly	Information

							prevalence of neuropsychiatric symptoms (NPS) such as agitation, depression, and sleep disturbances and the daily use of medication, including analgesic drug prescription.	more analgesics compared with patients without cancer but with dementia. Compared with patients without dementia but with cancer, patients with comorbid dementia and cancer had significantly more NPS, including sleep disturbances and agitation	
Drageset et al. – 2012 [20]	Cognitively intact NH residents 65 years or older	Cancer in nursing homes: characteristics and health-related quality of life among cognitively intact residents with and without cancer	2042 long-term-care residents from the 30 NHs in Bergen, Norway	Cross-sectional observation study	All cognitively intact NH residents 65 years or older who were capable of conversing and had been residing in the NH for at least 6 months	Single face-to-face interviews	Differences in HRQOL, controlled for age, gender, marital status, education, length of stay, and comorbidity	Residents with cancer reported more bodily pain (P = .17). General health was worse than that of the residents without cancer (P = .04) after adjusting for sociodemographic variables but not for comorbidity (P = .06).	Information

Table S6. Characteristics of studies, symptoms.

Authors – Year	Population	Object	Setting	Design	Inclusion Criteria	Intervention	Main Outcome Measures	Results	Principal Source of Bias
Blytt et al. – 2018 [19]	1825 NH patients with and without	Comorbid Dementia and Cancer in Residents	64 nursing homes, Norway	Cross-sectional study	All patients who had lived in the NH for at	Medical record, standardized interviews	Prevalence and comparison of cancer	Cancer is a risk factor of receiving more	Selection

	dementia and cancer	of Nursing Homes: Secondary Analyses of a Cross-Sectional Study			least 2 weeks	with the patient's primary caregiver	among patients with and without dementia. Prevalence of neuropsychiatric symptoms	analgesics in patients with dementia. Patients with comorbid dementia and cancer had significantly more neuropsychiatric symptoms, suggesting inadequate relief.	
Wergeland Sørbye et al. – 2019 [21]	100 NH residents	Opioids, Pain Management, and Palliative Care in a Norwegian Nursing Home From 2013 to 2018.	Non-profit nursing homes in Oslo, Norway	Longitudinal study	Residents who died in the nursing home	Data from InterRAI assessment instrument annually and when de resident died; semi-structured interview with nurses on duty at the deathbed	Description of the use of opioids	Use of analgesic medication was different depending on the diagnosis: cancer (odds ratio [OR] = 3.9, P = 0.048), heart/coronary disease (and OR = 3.49; P = 0.023) or suffering from dyspnea (OR = 3.2; P = 0.074). The main reason to give opioids was pain (22%) or a combination of different reasons (22%). Opioids were used as a prophylaxis (18%) or for comfort (13%).	Volunteer

Drageset et al. – 2015 [22]	60 NH residents with cancer	Loneliness, loss, and social support among cognitively intact older people with cancer, living in nursing homes–a mixed-methods study	Nursing homes, Norway	Mixed-methods design, cohort study	Diagnosis of cancer, 65 years and older, cognitively intact, capable of conversing and residing in the nursing home for at least 6 months	Face-to-face interviews using the one-item loneliness scale and the social provision scale	Loneliness and social support and patient's experience with loneliness, social support, and loss.	Marital status (P=0.02) and the subdimension on reassurance of worth (P=0.001) were significantly associated with loneliness. This emotional state was alleviated by being engaged in activities, being in contact with other people, and occupying oneself.	Sampling
Drageset et al. – 2013 [23]	227 cognitively intact older residents	Emotional Loneliness Is Associated with Mortality Among Mentally Intact Nursing Home Residents with and Without Cancer: A Five-Year Follow-Up Study	30 nursing homes, Bergen, Norway	Cross-sectional design	Patients who scored 0,5 or less on the Clinical Dementia Rating Scale, and provided informed consent	Face-to-face interview and sociodemographic variables and medical records	Sociodemographic factors and illness variables association with mortality and emotional and social loneliness measured using the Social Provisions Scale influence on mortality among nursing homes residents with cancer.	Overall survival after 5 years was 17% for residents with cancer and 22% for residents without cancer. Depression and comorbidity were associated with significantly worse survival independent of a cancer diagnosis. Residents with cancer and symptoms of anxiety had worse survival than those	Information

								without anxiety symptoms (P = .02), but not found among the noncancer group.	
Monroe et al. – 2012 [24]	58 NH patients	Assessing Advanced Cancer Pain in Older Adults with Dementia at the End-Of-Life	Nursing homes, US	Cross sectional study	Nursing home residents with mild to very severe dementia	Retrospective chart audits of people with Alzheimer's disease in nine nursing homes in 2009.	Discomfort Behavior Scale score and the Cognitive Performance Scale score	Patients with severe cognitive dysfunction have less pain behaviors. Medications administered over the 2 weeks before death were not correlated with pain behavior. Patients with severe Alzheimer disease received less opioid.	Information
Godino C et al. – 2006 [25]	40 oncology patients	Nursing education as an intervention to decrease fatigue perception in oncology patients.	Cancer center in Barcelona, Spain	Randomized experimental study	Patients with colon or gastric cancer	Patient education program delivered by nurses multifaceted; including one-to-one education, training and counselling, audio-visual and computerized educational materials.	Functional Assessment of Cancer Therapy Fatigue scores (FACT-F); satisfaction with the nursing intervention on fatigue assessed with a self-completed questionnaire	Decrease in the level of fatigue in the experimental group, whereas the group of patients that did not receive this intervention showed an increase in fatigue level along the treatment.	Information
Berry et al. – 2007 [26]	9 family members of NH residents	The Pain of Residents with Terminal Cancer in USA Nursing Homes:	Nursing homes in south-central Wisconsin, US	Cohort study	Family members of nursing home residents who had end-stage cancer and	Unstructured interviews, demographic information, an assessment	Perspectives of family members of nursing home residents with terminal	Vision of the patient's pain and illness was different if family member believed the	Selection

		Family Members' Perspectives			had reported pain.	of the status of the resident's pain management.	cancer, about pain and pain management	pain could be better managed or not. Members who believed pain could possibly be better managed often took the role of advocate and follow the evolution, while those who thought no improvement was possible expressed no concerns about this aspect of care as it was part of the illness and represented a barrier to the resident's access to adequate pain management.	
Pimentel et al. – 2015 [27]	8,094 NH residents with cancer	Pain Management in Nursing Home Residents with Cancer	Nursing homes, US	Cross-sectional study.	Newly admitted, Medicare-eligible NH residents with cancer	Nationwide data on NH resident health from the Minimum Data Set 2.0 linked to all-payer pharmacy dispensing records	Identification of all drugs dispensed within seven days of a resident's first prescription date.	65% of residents with cancer had any pain, among whom 13.5% had severe and 61.3% had moderate pain. 17% of residents in daily pain (CI [16.0;19.1%]) received	Information

								no analgesics, including 11.7% with severe pain (95%CI [8.9;14.5%]) and 16.9% with moderate pain (95%CI [15.1;18.8%]). Treatment was negatively associated with age >85 years, cognitive impairment, presence of feeding tube, and restraints.	
Dube et al. – 2018 [28]	367 462 NH residents with cancer	Cognitive Impairment and Pain Among Nursing Home Residents with Cancer	Nursing homes, US	Cross-sectional study	All newly admitted nursing home residents who were age 50 years and older with an active cancer diagnosis on their admission MDS in 2011 or 2012	Minimum Data Set 3.0 admission assessment	Pain (frequency, effect on function, intensity of pain) and pain management (any scheduled and as-needed pain medication, and any nonpharmacologic intervention for pain.)	Documentation of pain less frequent when severe cognitive impairment (severe versus no/mild cognitive impairment; self-assessed pain 0.77, 95%CI [0.76;0.78]; staff-assessed pain 0.96, 95%CI [0.93;0.99]). Pharmacologic management in 59.4% compared to 74.9% in those with no/mild cognitive	Information

							impairment).	
Drageset et al. – 2013 [29]	227 residents without cognitive impairment	Anxiety and Depression and Mortality Among Cognitively Intact Nursing Home Residents with and Without a Cancer Diagnosis: A 5-year Follow-Up Study	Nursing homes, Norway	Prospective observation study	All cognitively intact NH residents 65 years or older who were capable of conversing and had been residing in the NH for at least 6 months	Face-to-face interviews	Hospital Anxiety and Depression Scale (HADS) to assess anxiety and depression symptoms, with anxiety subscale (HADS-A) and depression subscale (HADS-D); assessment of ADLs by using the modified Katz Index of Independence	Anxiety symptoms were associated with worse survival compared to no symptoms. This association was not found in the group without cancer.	Information
Lukas et al. – 2013 [30]	3926 NH residents	Pain Characteristics and Pain Control in European Nursing Homes: Cross-Sectional and Longitudinal Results from the Services and Health for Elderly in Long TERm Care (SHELTER) Study	Nursing homes, Czech Republic, England, Finland, France, Germany, Netherlands, Italy and Israel	Cross-national study	57 nursing home facilities where residents were assessed by the inter-RAI instrument for Long Term Care Facilities (the interRAI LCTF), from Czech Republic, England, Finland, France, Germany, Netherlands, Italy and Israel	Data from interRAI instrument for Long Term Care Facilities (interRAI LTCF)	Validation of the interRAI LCTF as methodology to assess provision of care in European nursing homes and to implement this instrument over a large number of different European countries and to create a database as a basis for comparison across countries; Pain (frequency, constancy,	Almost 50% of residents suffered from pain, they have significantly less often dementia (P < .001). Pain was associated with unstable health conditions, cancer and depression; and was negatively associated with dementia.	Information

							intensity and control)		
Teno et al. – 2004 [31]	2,138,442 residents	Daily Pain That Was Excruciating at Some Time in the Previous Week: Prevalence, Characteristics, and Outcomes in Nursing Home Residents	Nursing homes in US	Cross-sectional study.	Persons just recently admitted to a NH as well as long-stay NH residents with a quarterly or annual MDS assessment.	Data from version 2.0 of the MDS	Pain reported as daily and at its most excruciating in the previous week on the MDS at initial and follow-up assessment	3.7% of residents had daily pain that was at least one time excruciating in the previous week, reported more frequently in younger patients. 62.1% of them needed assistance in self-care, in activities of daily living but 48.8% had a normal cognitive status. More than 20% with daily pain that was excruciating at times had a cancer diagnosis, and 21.5% experienced weight loss.	Selection
Duncan et al. – 2009 [32]	10,932 NH residents	Symptom Occurrence and Associated Clinical Factors in Nursing Home Residents with Cancer	Nursing homes, Kansas City, US	Cross-sectional study	All long-term care nursing home residents in the state of Kansas admitted between January 1, 2001 and December 31, 2003	Data from MDS	Pain (MDS Pain Scale, intensity, frequency and Intensity), shortness of breath, vomiting, weight loss, and diarrhea	Pain, shortness of breath, vomiting, weight loss, and diarrhea were significantly (p < .05) more prevalent in residents with cancer. Cancer treatments were	Information

								significantly related to fewer delirium indicators.	
Hunnicutt et al. – 2017 [33]	78,160 Medicare beneficiaries with cancer in NH	Hospice Use and Pain Management in Elderly Nursing Home Residents with Cancer	Nursing homes, US	Descriptive retrospective study	All Medicare beneficiaries with a validated date of death in 2011-2012 who had a comprehensive or quarterly MDS assessment <90 days before death, residents with an active MDS diagnosis of cancer listed	Data from Medicare Master Beneficiary Summary File and MDS 3.0, a medical record review for analgesic use	Pain and pain management at the last MDS assessment, severity was measured using either the Verbal Descriptor Scale or the Numeric Rating Scale	Hospice is associated with increased pain management with scheduled and PRN medications. Prevalence of pain was higher in residents who receive hospice care (59,9% vs 50%). Untreated pain was uncommon (self-reported pain: 2.9% and 5.6% in hospice users and nonusers, respectively).	Information
Monroe et al. – 2013 [34]	55 NH residents with dementia who died from cancer	Pain and hospice care in nursing home residents with dementia and terminal cancer	Nursing homes, US	Cross-sectional observation study	Nursing homes were stratified to include for-profit and not-for-profit, ranging in size from 68–180 beds located in and up to 150 miles from a large southern US metropolitan area (population >1.5 million). Decedents had to have	Data from MDS, chart review	Dementia severity (Cognitive Performance Scale; [CPS] score); pain/discomfort (Discomfort Behavior Scale [DBS]) score; and opioid administration over the last 2 weeks of life.	45% of residents were in hospice at the end-of-life. It increases likelihood of opioid pain treatment in residents with dementia and terminal cancer (OR = 3.9, 95%CI [1.1;14.0], P = 0.037). They were more likely	Information

					a diagnosis of dementia and must have died from cancer in the nursing home			to receive an opioid analgesic during the last 2 weeks of life (80% vs 43%, P = 0.005), but less likely to show severe cognitive impairment (20% vs 50%, P = 0.050). Severe cognitive impairment was associated with a higher risk of suffering from untreated advanced cancer pain OR = 0.3, 95%CI [0.1;0.8], P = 0.030). 40% of NH residents with dementia who died from cancer did not receive any opioid at the end-of-life.	
Reblin, et al. – 2017 [35]	58 nurses, 101 hospice cancer patient and spouse caregiver dyads	Caregiver, patient, and nurse visit communication patterns in cancer home hospice.	10 hospice agencies, New Jersey US	Descriptive study	Caregivers to individuals with a cancer diagnosis newly enrolled in-home hospice, over 45 years of age, spoke English, cognitively	Audio recorded visits	Definition of 2 dimensions: the patient/caregiver interacting with the nurse and the relatively high or low expression of distress	6 visit communication patterns were identified and defined by 2 dimensions: (1) either the patient and/or caregiver interacting with the nurse and	Information

					able to participate, and had at least 1 visit with their hospice nurse recorded		during the visit	(2) the relatively high or low expression of distress during the visit. Time until death significantly predicted several clusters.	
Liu et al. – 2019 [36]	3268 Medicare beneficiaries	Adjuvant Use and the Intensification of Pharmacologic Management for Pain in Nursing Home Residents with Cancer: Data from a US National Database.	Nursing homes, US	Cohort study	Active cancer diagnosis on MDS admission assessment; continuous Part D enrollment for ≥ 90 days after admission; ≥ one Part D claim for an opioid within 7 days of admission	MDS 3.0. Part D claims provided information on opioid and adjuvant use during the 7 days after admission and 90 days later. Evaluation of the medication at admission period (within 7 days of admission); and 90 days after admission.	Prevalence of adjuvants to opioid therapy and changes in these agents for pharmacologic management in nursing home residents with cancer.	20% received adjuvants at admission and gabapentin was the most common at 34.4%. Advanced age (≥85; aOR 0.80, 95%CI [0.63;1.02]) and comorbidities including dementia (aOR 0.65, 95%CI [0.53;0.82]) and depression (aOR: 1.55, 95%CI [1.29;1.87]) were associated with adjuvant use at admission. At 3 months, worse cognitive impairment (aOR: 0.80, 95%CI [0.64;0.99]) and presence of severe pain (aOR: 1.60,	Information

								95%CI [1.26;2.03]) were associated maintained or intensified analgesics in 25% of patients.	

Table S7. Characteristics of studies, advanced care planning.

Authors – Year	Population	Object	Setting	Design	Inclusion Criteria	Intervention	Main Outcome Measures	Results	Principal Source of Bias
Tseng et al. 2017 [37]	– 213 elderly NH residents	Factors Associated with the Do-Not-Resuscitate Decision Among Surrogates of Elderly Residents at a Nursing Home in Taiwan	Nursing home, northern Taiwan	Cross-sectional survey	Availability of family surrogates of elderly residents (65 years of age) and willingness to participate in this study	Questionnaires distributed to family surrogates or used in a face-to-face interview	Factors associated with DNR decision	Only 11.3% of residents had a signed DNR will or consent form. It depends on the culture, public knowledge toward DNR, legislation, and LTC facilities' policy, and nursing staff's workload and competence in communicating DNR with residents and family surrogates. Severe brain injury, a diagnosis of pulmonary disease or cancer are triggers for DNR decisions. Cancer residents	Selection

								have a higher likelihood of completed DNR forms.	
Epstein et al. – 2019 [38]	67 cancer patients	Giving Voice to Patient Values Throughout Cancer: A Novel Nurse-Led Intervention	3 clinics for patients with hematologic or gastrointestinal malignancies, New York, US	Pilot study	> 21 years or older, English-speaking, with a new diagnosis of cancer made or confirmed at Memorial Sloan Kettering (MSK) Institutional Review Board, establishing care at MSK	Trained oncology nurses for using specific questions and an empathic communication framework to discuss health-related values during outpatient clinic visits.	Feasibility and acceptability	Patients founded discussions helpful and felt comfortable with the process. Professionals perceived an improvement of care quality by normalizing such discussions and establishing a framework for shared decision making going forward.	Volunteer
Walling et al. – 2017 [39]	Approximately 10,000 patients with advanced cancer	Effect and efficiency of an embedded palliative care nurse practitioner in an oncology clinic	2 oncology clinic, Los Angeles, US	Prospective study	Patients with incurable referred by oncologists	Simultaneous care model by embedding a palliative care nurse practitioner in an oncology clinic. Implementation strategies (structured referral mechanism, routine symptom screening, integration of a psychology-based cancer supportive	Tasks (Symptom Assessment Basic, Symptom Assessment Medical, Symptom Assessment Psychosocial, Communication Basic, and Communication Complex) and then computed time spent	It increased the likelihood to document advance care planning (30% v 17%; P, .01) and to receive referral for psychosocial support (46% v 16%; P,.01) compared to no palliative care nurse.	Sampling

						care center, implementation team meetings, team training, and a metrics dashboard for continuous quality improvement).			
Dyason et al – 2015 [40]	166 LTC residents	Advance care planning discussions among residents of long-term care and designated assisted living: experience from Calgary, Alberta	Long term care and designated assisted living on Calgary, Canada	Evaluation and quality improvement audit	Older than 50 years old, minimum length of stay of 45 days in LTC or 90 days in DAL.	Review of tracking forms and charts about participation in ACP at baseline, 6 months and 18 months after policy implementation	Documentation of ACP orders, participation of the patient, presence or not of cognitive impairment	Resuscitative care orders: 40% in DAL 15% in LTC; medical care to prolong life 35% in DAL 55%; comfort care 6% in DAL and 25% in LTC. Residents were present in conversation 46%, not participation 41%; documented family member in 28%. Resident with cognitive impairment documented present in 20%. Presence of a nurse in 67%, physicians 34% social worker 16%.	Information
Bai et al. – 2010 [41]	146 patients with advanced cancer, 66	Attitudes towards palliative care among	One provincial and one municipal	Cross-sectional survey	Patients who were diagnosed with cancer	Structured questionnaires	Attitudes of terminally ill patients and health	The majority accepted death as a	Information

	doctors and 106 nurses	patients and health professionals in Henan, China	oncology hospital, and three community healthcare centers, Henan, China		at stage IV in the participating oncology hospitals, frontline doctors and nurses working in the participating community healthcare centers and hospitals		professionals towards palliative care	natural life process, but nearly half of the patients believe life preservation was their major care goal. They were unclear about their diagnosis and prognosis. The majority of health professional respondents affirmed the importance of palliative care. Less than half was trained or were willing to deliver the relevant care.	
Lawrence et al. – 2017 [42]	247 residents	Identification of the trajectory of functional decline for advance care planning in a nursing home population	4 nursing home facilities, southern Tasmania	Retrospective, longitudinal study	Patients admitted to the NH after March 1997 and whose death occurred before March 2008.	Paper-based records of deceased residents, Cause-of-death data were obtained by data matching with the Tasmanian register of Births, Deaths and Marriages (BDM).	Shape of the trajectories of functional decline grouped by their diagnosis, how trajectories can improve end of-life care planning	Patients with organ failure have a linear average functional decline with 6.5 points per year, and average function score at death of 68.6, CI [62.4;74.8]. The cancer and frail residents had significant curvilinear terms. The frail residents had a	Information

								slower rate of decline at 9.54 points per year and were most care dependent at death, with an average function score of 77.1, CI [73.8; 80.9].	
Dobalian – 2005 [43]	5899 NH residents	Advanced care planning documents in nursing facilities: results from a nationally representative survey	815 facilities, US	Retrospective cohort study	Facility or distinct unit of a facility that was either certified by Medicare or Medicaid or licensed as a nursing facility with 3 or more beds and providing onsite 24-hour skilled nursing care, residents living in the facility on January 1 1996 and those admitted during 1996.	Data from 1996 Nursing Home Component (NHC) of the Medical Expenditure Panel Survey (MEPS), MDS	Presence of documents Do-Not-Resuscitate orders, feeding/medication/other treatment restrictions and living wills correlated with clinical, demographic, facility, and regional factors.	DNR orders were more prevalent among residents aged 75 and more and those with severe cognitive impairment, dementia, emphysema, and cancer, but less common among African Americans and Latinos than whites.	Information

TableS 8. Characteristics of studies, end of life.

**Authors – year**	**Population**	**Object**	**Setting**	**Design**	**Inclusion criteria**	**Intervention**	**Main outcome measures**	**Results**
Sommerbak k et al – 2016 [44]	20 employees	Barriers to and facilitators for implementing quality improvements in palliative care – results from a qualitative interview study in Norway	2 hospitals, 1 nursing home, 2 local medical centers, Norway	Qualitative interview study	Employees directly involved in providing clinical PC (nurses and physicians) or involved in the organization of PC (managers). At least one year working experience within the service	Semi-structured interviews	Barriers or facilitators for improving PC in cancer and dementia settings	Three specific barrier diverse characteristic symptom asse inappropriate, tens changes at odds with care philosophy medical focus) and la

Irvin et al – 2000 [45]	5 registered nurses	The experiences of the registered nurse caring for the resident dying of cancer in a nursing home	Nursing home, Australia	Qualitative single case study	Minimum of 12 months experience working in the nursing home this setting,	Unstructured in-depth interviews and reflective journals.	Experiences of caring for a resident who was in the palliative care phase of a cancer illness.	Exclusivity of th management of p impacts of caring
Davies N et al – 2013 [46]	7 professionals providing palliative care for patients with either cancer or dementia	Quality palliative care for cancer and dementia in five European countries: some common challenges: investigate the factors that affect the quality of palliative care and care of dying, particularly but not exclusively for dementia syndrome	Cancer and palliative, primary, secondary and tertiary care settings from 5 European countries (England, Germany, Italy, Netherlands, Norway)	Semi-structured interviews, qualitative study	Clinical practitioners who provide dedicated palliative care within the settings, service management and those developing and implementing high level guidelines and policies designed to support high quality palliative care in primary, secondary and tertiary care	Semi-structured interviews	Challenges	5 common challenge difficulties; the va structural/functional services; the difficul palliative care serv processes of ca definitions, knowle inclusiveness) and constraints.
Johnston et al – 2016 [47]	121,458 NH residents	Increasing our understanding of dying of breast cancer: Comorbidities and care	Nursing homes, Nova Scotia, Canada	Population-based study	All 121,458 Nova Scotia residents who died in the province from 1995 to 2009. These decedents were identified in the Nova Scotia Vital Statistics (VS) database.	Linked administrative data from population-based registries were used to examine deaths in Nova Scotia from 1995-2009	Palliative program enrollment, time from palliative program enrollment to death, and place of death (hospital, nursing home, own home).	Mean age of breast was similar to th decedents (72.0 vers age spread was gre 23.1% versus 16.7%; versus 6.5%). Among breast cancer, 15.6% w diabetes registry a cardiovascular regist cancer decedents, decedents were twic dementia as a cause less likely to die in likely to die in a nu cancer decedents ha rates more similar t cancer decedents.
Drageset et al – 2013 [48]	227 cognitively intact older residents	Mortality in Nursing Home Residents Without Cognitive Impairment and Its Relation to Self-Reported Health-Related Quality of Life, Sociodemographic Factors, Illness Variables and Cancer Diagnosis: A 5-year Follow-Up Study	30 nursing homes in Norway	Cross-sectional study	All cognitively intact NH residents aged 65 years and older who were capable of carrying out a conversation, and had been residing in the NH for at least 6 months	Face-to-face interview	Self-reported HRQOL on SF-36 scale compared to general population and to compared to residents without a cancer diagnosis	Survival did not residents with and w 0.31). Twenty percer with cancer and 13 were still alive. Aft sociodemographic ar increasing age (education (P = 0.009 0.04) and the subd functioning (P = mortality. Bodily marginally associate = 0.08)
Bainbridge et al – 2015 [49]	1196 NH residents deceased of cancer cause	Factors Associated with Acute Care Use Among Nursing Home Residents Dying of Cancer: A Population-Based Study	Nursing homes, Ontario, Canada	Population-based, retrospective, cohort study	All decedents with a cancer-confirmed cause of death from the Ontario Cancer Registry, lived in LTC 6-7 months (182–212 days) before death	Data extracted form administrative healthcare datasets	Emergency department visit: the dichotomous variable defined as whether or not there was an emergency department visit in the last 6 months of life; Death in acute care: the dichotomous variable was defined as whether or not the person died in hospital or emergency department	61% had visited department in the la and 20% had died in associated with in having an emergenc were diabetes (OR 2 dementia (1.91, C congestive heart [1.12;2.93]) (all P<0.05 with death in hospita department were dia [1.07;2.25]), deme [1.05;2.03]), renal dis 2.66]), or COPD (1.55 P<0.03) .
Boyd et al – 2019 [50]	286 deceased residents	End of Life Care for Long-Term Care Residents with	Long term facilities, New Zealand	Prospective survey	Random cluster sample of 61 representative facilities. Facilities were	Standardized questionnaires – Symptom Management (SM-EOLD) and Comfort Assessment in End of life with Dementia	Trajectories before death in LTC for those with cancer, dementia or chronic illness including resident's condition 1 month before death,	Diagnosis at the tir dementia (49%), chr cancer (17%), and de (4%). There was no SM-EOLD in the las diagnosis (cancer dementia 26.5 (SD 8

		Dementia, Chronic Illness and Cancer: Prospective Staff Survey			stratified by region, size (up to 70 beds, and over 70 beds), and by for-profit or not-for-profit status.	(CAD-EOLD) – were administered to staff after the resident's death.	identification of sentinel events, symptom management 1 month prior to death using the SM-EOLD, such as management of restlessness, depression, fear and anxiety, pain, shortness of breath, and skin breakdown	26.9 (SD 8.6)). There v mean CAD-EOLD s before death by di 34.4(SD 5.2), chronic i cancer 33.3(SD 5. dementia had more symptoms and those less anxiety.
Kiely et al – 2002 [51]	120 348 newly admitted and long-stay residents	Common and gender specific factors associated with one-year mortality in nursing home residents	2 nursing home facilities, state of New York, US	Retrospective cohort study	> 65 years of age, resided 665 newly admitted or 647 long-stay in New York State nursing homes facilities between June 1994 and December 1997, death information was known	Minimum Data Set	Measures of health, functional, cognitive, psychological and social status	Cancer was more s with mortality in wor men (HR 2.21) in residents.
Cagle et al – 2020 [52]	1209 participants who died between 2012 and 2014	Hospice Utilization in the United States: A Prospective Cohort Study Comparing Cancer and Noncancer Deaths.	US	Prospective cohort study		Data from the 2012 survey wave to predict hospice use in general, and then separately for cancer and non-cancer deaths.	Hospice utilization rate for cancer and non-cancer death	Hospice utilization ra 70.8% for cancer dea noncancer deaths. A health OR 0.79, dem cancer OR 5.47 were higher odds of r Among cancer deaths and female gender (only predictors of hos

Table S9. Characteristics of studies, nursing home care.

Authors - Year	Population	Object	Setting	Design	Inclusion Criteria	Intervention	Main Outcome Measures	Results	Principal Source of Bias
Zueger et al – 2019 [53]	88 957 beneficiaries	Older Medicare Beneficiaries Frequently Continue Medications with Limited Benefit Following Hospice Admission	Nursing homes, Chicago, US	Cohort study	Enrolled and subsequently died in hospice between January 1, 2008, and December 31, 2013, any enrollment in a Medicare Advantage plan, 66 years and older who were admitted to and died under hospice care (for cancer and non-cancer causes)	Following of dispensing of limited benefit medications from SEER-Medicare database	Continuation of at least one LBM between the date of admission in hospice care and date of death	Patients admitted to hospice for cancer continued at least one LBM after admission in 29.8%, and 30.5% for patients with non-cancer related cause. The medications most frequently continued were anti-dementia (29.3%) while antiosteoporosis medications were less often	Selection

								maintained (14.1%). LBM continuation was greater in skilled nursing compared to home hospice (RR 1.25, 95%CI [1.20;1.29]), non-skilled nursing (RR 1.29, 95%CI [1.25;1.32]), and assisted living facilities (RR 1.28, 95%CI [1.24;1.32]).	
Drageset et al – 2015 [54]	227 LTC residents	Acute Hospital Admission for Nursing Home Residents Without Cognitive Impairment with a Diagnosis of Cancer	30 nursing homes, Bergen, Norway	Cross-sectional design	Age 65 years or older, resided in the NH for at least 6 months and cognitively intact and was capable of carrying out a conversation	Data obtained on Social Provision Scale (SPS) through face-to-face interviews	Time from inclusion in the sample in 2004–2005 until the first hospital admission. For residents with no hospital admission after inclusion, it was the time until the end of follow-up (in 2010). For people dying in the NH before the first hospital admission, the time until death was recorded and treated it as a censored	Residents with cancer had more admissions (25/60) than those without (53/167) (odds ratio 1.7). Social integration was correlated with admission (P = 0.04) regardless of cancer diagnosis.	Volunteer

							admission time		
Mackenzie et al – 2016 [55]	7324 patients with cancer and 23,871 caregivers	Unique Correlates of Heart Failure and Cancer Caregiver Satisfaction With Hospice Care	Hospice care, US	Retrospective cohort study	Caregivers of adult hospice patients who reported that their care recipient received hospice care primarily for heart failure or cancer, answered the FEHC in English, and answered the demographics section of the FEHC completely.	National data collected in 2011 by the National Hospice and Palliative Care Organization using the 61-item Family Evaluation of Hospice Care survey sent out 1 to 3 months after patient death. Family Evaluation of Hospice Care responses of adult heart failure and cancer caregivers	Relationship between possible correlates and caregiver satisfaction including caregiver and patient demographics, patient clinical characteristics, and hospice characteristics.	Need of hospice care in an LTC was more than twice likely to happen to heart failure patients compared to cancer patients (29% vs. 13%, P < 0.0001) and was associated with lower satisfaction in both groups	Sampling
Meijers et al – 2009 [56]	12 883 patients	Malnutrition Prevalence in The Netherlands: Results of the Annual Dutch National Prevalence Measurement of Care Problems	All healthcare organizations, Netherland	Cross-sectional, multi-center design	Patients of age 18 years and above from all healthcare organizations in The Netherlands including university and general hospitals, nursing homes and homecare organizations	Standardized questionnaire	Prevalence of malnutrition, association of factors such as age, sex, disease, ward type and time since admission.	Prevalence of malnutrition was the highest in hospitals (23·8 %), homecare organizations (21·7 %) and nursing homes (19·2 %). Logistic regression analysis revealed no association with age, time since admission and ward type. Being female was associated with malnutritio	Information

								n only in nursing homes. Blood diseases, gastrointestinal tract diseases, infection, chronic obstructive pulmonary disease, dementia and cancer were the factors associated with malnutrition in hospitals. Dementia was associated with malnutrition in nursing homes, while gastrointestinal tract diseases, diabetes mellitus and cancer were the associated factors in home care.	
Aamodt et al – 2013 [57]	10 nurses	Nurses' perspectives on the discharge of cancer patients with palliative care needs from a gastroenterology ward.	Gastroenterology surgical ward at the university hospital, Norway	Descriptive study	Registered nurse, minimum 3 months’ nursing experience with cancer patients receiving palliative care on the ward, experience with patient discharge planning, able to	Focus group interviews	Nurses’ recommendations for optimal patient follow-up care after discharge	Two themes: ‘the complexity of and fluctuations in the patients’ health status’ and ‘considering the competency of the nurses at the discharge destinations	Recall

					participate in a 60–90-minute session, and able to speak and read Norwegian in order to consent to participation				
Graziano et al – 2019 [58]	1843 residents with polypharmacy	Deprescribing in Nursing Home Residents on Polypharmacy: Incidence and Associated Factors.	Nursing homes, Europe and Israel	Longitudinal multicenter cohort study	57 nursing home facilities where residents were assessed by the inter-RAI instrument for Long Term Care Facilities (the interRAI LCTF), from Czech Republic, England, Finland, France, Germany, Netherlands, Italy and Israel	Data from the Services and Health in Long TERm care study (SHELTER)	Incidence of deprescribing (defined as a reduction in the number of medications used over the study period) and factors related to deprescribing Polypharmacy defined as the concurrent use of 5 or more medications	Mean number of medications was 8.6 at the baseline. Deprescribing was observed in 35.6% of residents. Risk factors for deprescription were cognitive impairment (mild/moderate OR 1.41, 95%CI [1.11;1.79], severe OR 1.41, 95%CI [1.15;1.72]), presence of the geriatrician within the facility staff (OR 1.41, 95%CI [1.15;1.72]), and number of medications used at baseline (OR 1.10, 95%CI [1.06; 1.14]). The factors associated with a lower probability of	Selection

								deprescribing were cancer (OR 0.64, 95%CI [0.45;0.90]), heart failure (OR 0.69, 95%CI [0.53;0.89]), and female gender (OR 0.76, 95%CI [0.61;0.96]).	
Lage et al. – 2020 [59]	34.670 deceased older NH residents with cancer	Potentially Burdensome End-of-Life Transitions among Nursing Home Residents with Poor Prognosis Cancer	Nursing homes, US	Retrospective analysis	Deceased older adults (≥65 years old) with poor prognosis solid tumors who resided in US NHs and enrolled in Medicare fee-for service (2008-2009), residing in a NH over 100 days within the study eligibility period of 1/2008 – 12/2009 (i.e., long-stay resident) with no more than 10 consecutive days outside the facility during the 100 day eligibility period, continuous Medicare enrollment, and not on Medicare Advantage	Data from Medicare claims (Parts A and B), MDS files and OSCAR NH dataset.	Any potentially burdensome transition in the last 90 days of life: 2 or more hospitalizations or an intensive care unit admission	53,8% had moderate-to-severe cognitive impairment, full dependence in activities of daily living (66.5%), and comorbidities like congestive heart failure (29.3%) and chronic obstructive pulmonary disease (34.1%). 55.4% of patients used hospice at any time in the 90 days before death. 36.0% of patients experienced a potentially burdensome EOL transition, which were higher among patients who did not receive hospice	Information

								(45.4% vs. 28.7%, p<0.01). Full dependence in ADLs (OR 1.70, P<0.01), CHF (OR 1.48, P<0.01), and COPD (OR 1.28, P<0.01) were associated with higher risk of burdensome EOL transition. Those with do-not-resuscitate directives (OR 0.60, P<0.01) and impaired cognition (OR 0.89, p<0.01) had lower odds of burdensome EOL transition.	

Table S10. Characteristics of studies, education.

Authors - Year	Population	Object	Setting	Design	Inclusion Criteria	Intervention	Main Outcome Measures	Results	Principal Source of Bias
Nagamatsu et al – 2014 [60]	188 nurses	Impact of a nursing education program about caring for patients in Japan with malignant pleural mesothelioma on nurses' knowledge, difficulties and	Hospitals with respiratory wards or palliative care wards, cancer hospitals, home visiting nurse stations and health care centers, Japan	Randomized controlled study	Recruitment by advertisement letters sent nationwide to the heads or nursing directors of health care facilities targeting hospitals with respiratory wards or	2-day program with a half-day follow-up program included lectures, group work, role-playing and group discussion. Knowledge, difficulties and attitude were measured at	Knowledge of and treatment of MPM, difficulties in caring for patients with MPM and attitude toward care for patients with MPM.	The knowledge, difficulty and attitude scores were favorable with the intervention post-test and follow-up test 1 month later. The Knowledge score (t = 14.03, *p* =	Selection

		attitude: a randomized control trial			palliative care wards, cancer hospitals, home visiting nurse stations and health care centers.	baseline and a post-test and at follow-up one month later.		0.000) and follow-up test (t = 8.98, *p* = 0.000). Difficulty score (t = −3.41, *p* = 0.001) and follow-up test (t = −3.70, *p* = 0.000). The Attitude score (t = 7.11, *p* = 0.000) and follow-up test (t = 4.54, *p* = 0.000).	
Gerhart et al – 2017 [61]	12 clinicians from an SCT team and inpatient oncology nursing staff	Brief Training on Patient Anger Increases Oncology Providers' Self-Efficacy in Communicating with Angry Patients.	Midwestern academic medical center, Chicago, US	Posttest design	Providers working in stem cell transplant and oncology units	Brief provider training program by cognitive behavioral models of anger that included didactics, discussion, and experimental training on communication and stress management.	Feasibility for increasing providers' self-efficacy in responding to patient anger	Provider-rated self-efficacy was significantly higher for nine of 10 skill outcomes (*p* < 0.005) including acknowledging patient anger, discussing anger, considering solutions, and using relaxation to manage their own distress.	Volunteer
Passik SD et al – 2000 [62]	14 oncologists and 18 nurses	Oncology staff recognition of depressive symptoms on videotaped interviews of depressed cancer patients: implications for designing a	Rural community, hospital-based outreach network, Indiana, US	Posttest design	Not informed	It was given a one-hour in-service on the use of the Mini International Neuropsychiatric Interview to provide a differential diagnosis. Then, staff viewed a videotape of	MD and RN Concordance Rates	Average concordance with the experts (86% overall agreement) among oncologist and nurse raters for somatic symptoms (Case 1 82%; Case 2 91%) was higher overall than	Sampling

		training program.				an investigator utilizing the MINI to interview two depressed breast cancer patients and rated depressive symptoms and made a diagnosis.		for cognitive symptoms (78% overall; Case 1 91%; Case 2 65%). Raters reported a greater level of confidence about their diagnosis for Case 1 (94%) than for Case 2 (64%).	
Sommerbakk et al – 2016 [44]	20 employees	Barriers to and facilitators for implementing quality improvements in palliative care – results from a qualitative interview study in Norway	2 hospitals, 1 nursing home, 2 local medical centers, Norway	Qualitative interview study	Employees directly involved in providing clinical PC (nurses and physicians) or involved in the organization of PC (managers). At least one year working experience within the service	Semi-structured interviews	Barriers or facilitators for improving PC in cancer and dementia settings	3 specific barriers to palliative care: diverse characteristics of patients makes symptom assessment tools inappropriate, tension caused by changes at odds with the holistic hospice care philosophy (example: more medical focus) and lack of expertise.	Selection
Hee-Soon et al – 2006 [63]	40 clinical nurses	Development and evaluation of a PBL-based continuing education for clinical nurses: a pilot study	University hospital, Seoul, Korea	Pilot study	Nurses at a university hospital located in Seoul	PBL packages with simulation on ACLS, program consisted of six 3-hour weekly classes and was evaluated by the	Evaluation of the program after its implementation: perceived advantages and disadvantages of PBL, and the possibility	57.5% of the participants responded positively about the use of PBL as continuing nurse education in terms of self-motivated	Selection

						participants’ subjective responses	to use the PBL method as a continuing education	and cooperative learning, whereas 20.0% of the participants answered that the PBL method was not suitable for clinical nurses.	
Bakker et al – 2010 [64]	12 focus groups with 91 cancer nurses	Canadian cancer nurses' views on recruitment and retention.	Canada	Cohort study	Registered nurses who provided direct care to cancer patients, full-time or part-time employed in cancer care settings (ambulatory care clinics, hospital units, homecare or community palliative care services)	Focus groups with cancer nurses to collect data with interviews about strategies that could improve recruitment and retention	Phase I: prospective survey design to determine workforce and professional practice factors present in oncology work environment and to test a conceptual model linking work environment factors to oncology nurse’s job satisfaction. Phase II: Data about strategies that could improve recruitment and retention.	Oncology needs to be recognized as a specialty, tacit knowledge is no longer enough, gratification is a retaining factor and relationship depends on environment. Job satisfaction requires continuing education opportunities. It is not only about administration of chemotherapy, but a combination of clinical practice experience in an inpatient setting along with life experiences necessary to deal with life-altering cancers. To keep oncology nurses,	Selection

								there must be a mutual agreement between the caregiver and the organization for investment in professional development.	
Fukui et al – 2011 [65]	8 nurses	Effectiveness of communication skills training of nurses on the quality of life and satisfaction with healthcare professionals among newly diagnosed cancer patients: A preliminary study	4 cancer screening institutions in east Japan	Secondary analysis of a randomized controlled trial	Newly diagnosed and informed of cancer, from January to December 2006; primary nurse present at the physician consultation; age over 18 years; not advanced and operable stage; diagnosed with gastric, colorectal, or breast cancer, the 3 major sites most tested at the center; and written informed consent.	Random assignation to an experimental group attending communication skills training program (4 nurses) or to a control group (4 nurses)	Effectiveness for patient quality of life and their satisfaction with healthcare professionals was assessed by administrating the SF-8 and a single-item VAS three times (1 week after diagnosis, 1 month after, and at 3 months)	Repeated measures analysis of variance showed a group-by-time significant increase of the mental aspects of SF-8 (F53.48; P50.03) and satisfaction with the nurse (F53.18; p50.04)	Selection

References

1. Koroukian, S.M.; Xu, F.; Beaird, H.; Diaz, M.; Murray, P.; Rose, J.H. Complexity of Care Needs and Unstaged Cancer in Elders: A Population-Based Study. *Cancer Detect Prev.* **2007**, *31*, 199–206, doi:10.1016/j.cdp.2007.04.002.
2. Hamaker, M.E.; Hamelinck, V.C.; van Munster, B.C.; Bastiaannet, E.; Smorenburg, C.H.; Achterberg, W.P.; Liefers, G.-J.; de Rooij, S.E. Nonreferral of Nursing Home Patients with Suspected Breast Cancer. *J Am Med Dir Assoc* **2012**, *13*, 464–469, doi:10.1016/j.jamda.2012.01.002.
3. Christos PJ; Oliveria SA; Mâsse LC; McCormick LK; Halpern AC Skin Cancer Prevention and Detection by Nurses: Attitudes, Perceptions, and Barriers. *J. Cancer Educ.* **2004**, *19*, 50–57, doi:10.1207/s15430154jce1901_12.

4. Bradley, C.J.; Clement, J.P.; Lin, C. Absence of Cancer Diagnosis and Treatment in Elderly Medicaid-Insured Nursing Home Residents. *JNCI: J. Natl. Cancer Inst.* **2008**, *100*, 21–31, doi:10.1093/jnci/djm271.
5. Yadav, N.R.; Jain, M.; Sharma, A.; Yadav, R.; Pahuja, M.; Jain, V. Distribution and Prevalence of Oral Mucosal Lesions in Residents of Old Age Homes in Delhi, India. *Nepal J. Epidemiol.* **2018**, *8*, 727–734, doi:10.3126/nje.v8i2.18708.
6. Clement, J.P.; Bradley, C.J.; Lin, C. Organizational Characteristics and Cancer Care for Nursing Home Residents. *Health Serv. Res.* **2009**, *44*, 1983–2003, doi:10.1111/j.1475-6773.2009.01024.x.
7. Rudel RJ On Breast Cancer Detection, Directors of Nursing and Female Residents: A Study in Rural Long-Term Care. *Online J. Rural Nurs. & Health Care* **2007**, *7*, 21–35, doi:10.14574/ojrnhc.v7i2.135.
8. Wildiers, H.; Mauer, M.; Elseviers, M.; De Wolf, J.; Hatse, S.; Hamaker, M.; Buntinx, F.; De Lepeleire, J.; Uytterschaut, G.; Falandry, C.; et al. Cancer Events in Belgian Nursing Home Residents: An EORTC Prospective Cohort Study. *J. Geriatr Oncol.* **2019**, *10*, 805–810, doi:10.1016/j.jgo.2019.03.005.
9. Darjani, A.; Alizadeh, N.; Rafiei, E.; Moulaei, M.; Naseri Alavi, S.H.; Eftekhari, H.; Rafiei, R.; Gharaei-Nejad, K.; Mohtasham-Amiri, Z. Skin Diseases among the Old Age Residents in a Nursing Home: A Neglected Problem. *Dermatol. Res. Pract.* **2020**, 1–5, doi:10.1155/2020/8849355.
10. Muss, H.B. Adjuvant Chemotherapy in Older and Younger Women With Lymph Node–Positive Breast Cancer. *JAMA* **2005**, *293*, 1073, doi:10.1001/jama.293.9.1073.
11. Liuu, E.; Guyot, N.; Valero, S.; Jamet, A.; Ouazzani, H.E.; Bouchaert, P.; Tourani, J.; Migeot, V.; Paccalin, M. Prevalence of Cancer and Management in Elderly Nursing Home Residents. A Descriptive Study in 45 French Nursing Homes. *Eur. J. Cancer Care* **2019**, *28*, N.PAG-N.PAG, doi:10.1111/ecc.12957.
12. Bern-Klug, M.; Singh, J.; Liu, J.; Shinkunas, L. Prospect Theory Concepts Applied to Family Members of Nursing Home Residents with Cancer: A Good Ending Is a Gain. *J. Soc. Work End Life Palliat Care* **2019**, *15*, 34–54, doi:10.1080/15524256.2019.1580242.
13. Tang, V.; Zhao, S.; Boscardin, J.; Sudore, R.; Covinsky, K.; Walter, L.C.; Esserman, L.; Mukhtar, R.; Finlayson, E. Functional Status and Survival After Breast Cancer Surgery in Nursing Home Residents. *JAMA Surg.* **2018**, *153*, 1090–1096, doi:10.1001/jamasurg.2018.2736.
14. Finlayson, E.; Zhao, S.; Boscardin, W.J.; Fries, B.E.; Landefeld, C.S.; Dudley, R.A. Functional Status after Colon Cancer Surgery in Elderly Nursing Home Residents. *J. Am. Geriatr Soc.* **2012**, *60*, 967–973, doi:10.1111/j.1532-5415.2012.03915.x.
15. Finlayson, E.; Zhao, S.; Varma, M.G. Outcomes after Rectal Cancer Surgery in Elderly Nursing Home Residents. *Dis. Colon Rectum.* **2012**, *55*, 1229–1235, doi:10.1097/DCR.0b013e318267bfe3.
16. Chan, T.Y.; Foo, C.C.; Law, W.L.; Lo, O. Outcomes of Colorectal Cancer Surgery in the Nonagenarians: 20-Year Result from a Tertiary Center. *BMC Surg.* **2019**, *19*, 155, doi:10.1186/s12893-019-0623-4.
17. Williams, G.R.; Dunham, L.; Chang, Y.; Deal, A.M.; Pergolotti, M.; Lund, J.L.; Guerard, E.J.; Kenzik, K.; Muss, H.B.; Sanoff, H.K. Geriatric Assessment to Predict Hospitalization Frequency and Long-Term Care Utilization in Older Adult Cancer Survivors. *J. Clin. Oncol.* **2018**, *36*, doi:10.1200/JCO.2018.36.15-suppl.10036.
18. Buchanan, R.J.; Barkley, J.; Wang, S.; Kim, M. Analyses of Nursing Home Residents with Cancer at Admission. *Cancer Nurs.* **2005**, *28*, 406–414, doi:10.1097/00002820-200509000-00012.
19. Blytt, K.M.; Selbæk, G.; Drageset, J.; Natvig, G.K.; Husebo, B.S. Comorbid Dementia and Cancer in Residents of Nursing Homes: Secondary Analyses of a Cross-Sectional Study. *Cancer Nurs.* **2018**, *41*, E13–E20, doi:10.1097/NCC.0000000000000478.
20. Drageset, J.; Eide, G.E.; Ranhoff, A.H. Cancer in Nursing Homes: Characteristics and Health-Related Quality of Life among Cognitively Intact Residents with and without Cancer. *Cancer Nurs.* **2012**, *35*, 295–301, doi:10.1097/NCC.0b013e31822e7cb8.
21. Wergeland Sørbye, L.; Steindal, S.A.; Kalfoss, M.H.; Vibe, O.E. Opioids, Pain Management, and Palliative Care in a Norwegian Nursing Home From 2013 to 2018. *Health Serv. Insights* **2019**, *12*, N.PAG-N.PAG, doi:10.1177/1178632919834318.
22. Drageset, J.; Eide, G.E.; Dysvik, E.; Furnes, B.; Hauge, S. Loneliness, Loss, and Social Support among Cognitively Intact Older People with Cancer, Living in Nursing Homes--a Mixed-Methods Study. *Clin. Interv. Aging* **2015**, *10*, 1529–1536, doi:10.2147/CIA.S88404.

23. Drageseth, J.; Eide, G.E.; Kirkevold, M.; Ranhoff, A.H. Emotional Loneliness Is Associated with Mortality among Mentally Intact Nursing Home Residents with and without Cancer: A Five-Year Follow-up Study. *J. Clin. Nurs.* **2013**, *22*, 106–114, doi:10.1111/j.1365-2702.2012.04209.x.
24. Monroe, T.; Carter, M.; Feldt, K.; Tolley, B.; Cowan, R.L. Assessing Advanced Cancer Pain in Older Adults with Dementia at the End-of-Life. *J .Adv. Nurs.* **2012**, *68*, 2070–2078, doi:10.1111/j.1365-2648.2011.05929.x.
25. Godino C; Jodar L; Durán Á; Martínez I; Schiaffino A Nursing Education as an Intervention to Decrease Fatigue Perception in Oncology Patients. *Eur. J. Oncol. Nurs.* **2006**, *10*, 150–155, doi:10.1016/j.ejon.2005.03.004.
26. Berry, P.H. The Pain of Residents with Terminal Cancer in USA Nursing Homes: Family Members' Perspectives. *Int. J. Palliat. Nurs.* **2007**, *13*, 20–27, doi:10.12968/ijpn.2007.13.1.22777.
27. Pimentel, C.B.; Briesacher, B.A.; Gurwitz, J.H.; Rosen, A.B.; Pimentel, M.T.; Lapane, K.L. Pain Management in Nursing Home Residents with Cancer. *J. Am. Geriatr Soc.* **2015**, *63*, 633–641, doi:10.1111/jgs.13345.
28. Dubé, C.E.; Mack, D.S.; Hunnicutt, J.N.; Lapane, K.L. Cognitive Impairment and Pain Among Nursing Home Residents With Cancer. *J. Pain Symptom Manag.* **2018**, *55*, 1509–1518, doi:10.1016/j.jpainsymman.2018.02.012.
29. Drageset, J.; Eide, G.E.; Ranhoff, A.H. Anxiety and Depression and Mortality among Cognitively Intact Nursing Home Residents with and without a Cancer Diagnosis: A 5-Year Follow-up Study. *Cancer Nurs.* **2013**, *36*, E68-74, doi:10.1097/NCC.0b013e31826fcb11.
30. Lukas, A.; Mayer, B.; Fialová, D.; Topinkova, E.; Gindin, J.; Onder, G.; Bernabei, R.; Nikolaus, T.; Denkinger, M.D. Pain Characteristics and Pain Control in European Nursing Homes: Cross-Sectional and Longitudinal Results from the Services and Health for Elderly in Long TERm Care (SHELTER) Study. *J. Am. Med. Dir. Assoc.* **2013**, *14*, 421–428, doi:10.1016/j.jamda.2012.12.010.
31. Teno, J.M.; Kabumoto, G.; Wetle, T.; Roy, J.; Mor, V. Daily Pain That Was Excruciating at Some Time in the Previous Week: Prevalence, Characteristics, and Outcomes in Nursing Home Residents. *J. Am. Geriatr. Soc.* **2004**, *52*, 762–767, doi:10.1111/j.1532-5415.2004.52215.x.
32. Duncan, J.G.; Bott, M.J.; Thompson, S.A.; Gajewski, B.J. Symptom Occurrence and Associated Clinical Factors in Nursing Home Residents with Cancer. *Res. Nurs. Health* **2009**, *32*, 453–464, doi:10.1002/nur.20331.
33. Hunnicutt, J.N.; Tjia, J.; Lapane, K.L. Hospice Use and Pain Management in Elderly Nursing Home Residents With Cancer. *J. Pain Symptom Manag.* **2017**, *53*, 561–570, doi:10.1016/j.jpainsymman.2016.10.369.
34. Monroe, T.B.; Carter, M.A.; Feldt, K.S.; Dietrich, M.S.; Cowan, R.L. Pain and Hospice Care in Nursing Home Residents with Dementia and Terminal Cancer. *Geriatr. Gerontol. Int.* **2013**, *13*, 1018–1025, doi:10.1111/ggi.12049.
35. Reblin, M.; Clayton, M.F.; Xu, J.; Hulett, J.M.; Latimer, S.; Donaldson, G.W.; Ellington, L. Caregiver, Patient, and Nurse Visit Communication Patterns in Cancer Home Hospice. *Psycho-Oncology* **2017**, *26*, 2285–2293, doi:10.1002/pon.4361.
36. Liu, S.-H.; Hunnicutt, J.N.; Ulbricht, C.M.; Dubé, C.E.; Hume, A.L.; Lapane, K.L. Adjuvant Use and the Intensification of Pharmacologic Management for Pain in Nursing Home Residents with Cancer: Data from a US National Database. *Drugs & Aging* **2019**, *36*, 549–557, doi:10.1007/s40266-019-00650-3.
37. Tseng, Y.-P.; Huang, L.-H.; Huang, T.-H.; Hsu, L.-L.; Hsieh, S.-I. Factors Associated With the Do-Not-Resuscitate Decision Among Surrogates of Elderly Residents at a Nursing Home in Taiwan. *Int. J. Gerontol.* **2017**, *11*, 95–99, doi:10.1016/j.ijge.2016.07.004.
38. Epstein, A.S.; Desai, A.V.; Bernal, C.; Romano, D.; Wan, P.J.; Okpako, M.; Anderson, K.; Chow, K.; Kramer, D.; Calderon, C.; et al. Giving Voice to Patient Values Throughout Cancer: A Novel Nurse-Led Intervention. *J. Pain Symptom Manag.* **2019**, *58*, 72-79.e2, doi:10.1016/j.jpainsymman.2019.04.028.
39. Walling, A.M.; D'Ambruoso, S.F.; Malin, J.L.; Hurvitz, S.; Zisser, A.; Coscarelli, A.; Clarke, R.; Hackbarth, A.; Pietras, C.; Watts, F.; et al. Effect and Efficiency of an Embedded Palliative Care Nurse Practitioner in an Oncology Clinic. *J. Oncol. Pract.* **2017**, *13*, e792–e799, doi:10.1200/JOP.2017.020990.
40. Dyason, C.; Simon, J.; Wityk Martin, T.L. Advance Care Planning Discussions among Residents of Long Term Care and Designated Assisted Living: Experience from Calgary, Alberta. *BMJ Support Palliat Care* **2015**, *5*, 48–53, doi:10.1136/bmjspcare-2013-000444.
41. Bai, Q.; Zhang, Z.; Lu, X.; Shi, Y.; Liu, X.; Chan, H. Attitudes towards Palliative Care among Patients and Health Professionals in Henan, China. *Prog. Palliative Care* **2010**, *18*, 341–345, doi:10.1179/1743291X10Y.0000000006.

42. Lawrence, S.; Robinson, A.; Eagar, K. Identification of the Trajectory of Functional Decline for Advance Care Planning in a Nursing Home Population. *Australas J. Ageing* **2017**, *36*, E14–E20, doi:10.1111/ajag.12454.
43. Dobalian, A. Advance Care Planning Documents in Nursing Facilities: Results from a Nationally Representative Survey. *Arch. Gerontol. Geriatr.* **2006**, *43*, 193–212, doi:10.1016/j.archger.2005.10.007.
44. Sommerbakk, R.; Haugen, D.F.; Tjora, A.; Kaasa, S.; Hjermstad, M.J. Barriers to and Facilitators for Implementing Quality Improvements in Palliative Care - Results from a Qualitative Interview Study in Norway. *BMC Palliat Care* **2016**, *15*, 61, doi:10.1186/s12904-016-0132-5.
45. Irvin, S. The Experiences of the Registered Nurse Caring for the Person Dying of Cancer in a Nursing Home. *Collegian* **2000**, *7*, 30–34, doi:10.1016/s1322-7696(08)60388-1.
46. Davies, N.; Maio, L.; van Riet Paap, J.; Mariani, E.; Jaspers, B.; Sommerbakk, R.; Grammatico, D.; Manthorpe, J.; Ahmedzai, S.; Vernooij-Dassen, M.; et al. Quality Palliative Care for Cancer and Dementia in Five European Countries: Some Common Challenges. *Aging Ment. Health* **2014**, *18*, 400–410, doi:10.1080/13607863.2013.843157.
47. Johnston, G.M.; Urquhart, R.; Lethbridge, L.; MacIntyre, M. Increasing Our Understanding of Dying of Breast Cancer: Comorbidities and Care. *Prog. Palliat. Care* **2016**, *24*, 147–152, doi:10.1080/09699260.2015.1108638.
48. Drageset, J.; Eide, G.E.; Ranhoff, A.H. Mortality in Nursing Home Residents without Cognitive Impairment and Its Relation to Self-Reported Health-Related Quality of Life, Sociodemographic Factors, Illness Variables and Cancer Diagnosis: A 5-Year Follow-up Study. *Qual. Life Res.* **2013**, *22*, 317–325, doi:10.1007/s11136-012-0143-5.
49. Bainbridge, D.; Seow, H.; Sussman, J.; Pond, G. Factors Associated with Acute Care Use among Nursing Home Residents Dying of Cancer: A Population-Based Study. *Int. J. Palliat. Nur.s* **2015**, *21*, 349–356, doi:10.12968/ijpn.2015.21.7.349.
50. Boyd, M.; Frey, R.; Balmer, D.; Robinson, J.; McLeod, H.; Foster, S.; Slark, J.; Gott, M. End of Life Care for Long-Term Care Residents with Dementia, Chronic Illness and Cancer: Prospective Staff Survey. *BMC Geriatr* **2019**, *19*, 137, doi:10.1186/s12877-019-1159-2.
51. Kiely, D.K.; Flacker, J.M. Common and Gender Specific Factors Associated with One-Year Mortality in Nursing Home Residents. *J. Am. Med. Dir. Assoc.* **2002**, *3*, 302–309, doi:10.1016/S1525-8610(05)70545-4.
52. Cagle, J.G.; Lee, J.; Ornstein, K.A.; Guralnik, J.M. Hospice Utilization in the United States: A Prospective Cohort Study Comparing Cancer and Noncancer Deaths. *J. Am. Geriatr. Soc.* **2020**, *68*, 783–793, doi:10.1111/jgs.16294.
53. Zueger, P.M.; Holmes, H.M.; Calip, G.S.; Qato, D.M.; Pickard, A.S.; Lee, T.A. Older Medicare Beneficiaries Frequently Continue Medications with Limited Benefit Following Hospice Admission. *J. Gen. Intern. Med.* **2019**, *34*, 2029–2037, doi:10.1007/s11606-019-05152-x.
54. Drageset, J.; Eide, G.E.; Harrington, C.; Ranhoff, A.H. Acute Hospital Admission for Nursing Home Residents without Cognitive Impairment with a Diagnosis of Cancer. *Eur. J .Cancer Care (Engl)* **2015**, *24*, 147–154, doi:10.1111/ecc.12205.
55. MacKenzie, M.A.; Buck, H.G.; Meghani, S.H.; Riegel, B. Unique Correlates of Heart Failure and Cancer Caregiver Satisfaction With Hospice Care. *J. Pain Symptom Manag.* **2016**, *51*, 71–78, doi:10.1016/j.jpainsymman.2015.09.001.
56. Meijers, J.M.M.; Schols, J.M.G.A.; van Bokhorst-de van der Schueren, M.A.E.; Dassen, T.; Janssen, M.A.P.; Halfens, R.J.G. Malnutrition Prevalence in The Netherlands: Results of the Annual Dutch National Prevalence Measurement of Care Problems. *Br. J. Nutr.* **2009**, *101*, 417–423, doi:10.1017/S0007114508998317.
57. Marie Thon Aamodt, I.; Lie, I.; Hellesø, R. Nurses' Perspectives on the Discharge of Cancer Patients with Palliative Care Needs from a Gastroenterology Ward. *Int. J. Palliat. Nurs.* **2013**, *19*, 396–402, doi:10.12968/ijpn.2013.19.8.396.
58. Onder, G.; Vetrano, D.L.; Villani, E.R.; Carfì, A.; Lo Monaco, M.R.; Cipriani, M.C.; Manes Gravina, E.; Denkinger, M.; Pagano, F.; van der Roest, H.G.; et al. Deprescribing in Nursing Home Residents on Polypharmacy: Incidence and Associated Factors. *J. Am. Med Dir. Assoc.* **2019**, *20*, 1116–1120, doi:10.1016/j.jamda.2019.01.130.
59. Lage, D.E.; DuMontier, C.; Lee, Y.; Nipp, R.D.; Mitchell, S.L.; Temel, J.S.; El-Jawahri, A.; Berry, S.D.; El-Jawahri, A. Potentially Burdensome End-of-Life Transitions among Nursing Home Residents with Poor-Prognosis Cancer. *Cancer (0008543X)* **2020**, *126*, 1322–1329, doi:10.1002/cncr.32658.

60. Nagamatsu, Y.; Natori, Y.; Yanai, H.; Horiuchi, S. Impact of a Nursing Education Program about Caring for Patients in Japan with Malignant Pleural Mesothelioma on Nurses' Knowledge, Difficulties and Attitude: A Randomized Control Trial. *Nurse Educ. Today* **2014**, *34*, 1087–1093, doi:10.1016/j.nedt.2014.02.007.
61. Gerhart, J.I.; Sanchez Varela, V.; Burns, J.W. Brief Training on Patient Anger Increases Oncology Providers' Self-Efficacy in Communicating With Angry Patients. J. *Pain Symptom Manag.* **2017**, *54*, 355-360.e2, doi:10.1016/j.jpainsymman.2017.07.039.
62. Passik SD; Donaghy KB; Theobald DE; Lundberg JC; Holtsclaw E; Dugan WM Jr.; Passik, S.D.; Donaghy, K.B.; Theobald, D.E.; Lundberg, J.C.; et al. Oncology Staff Recognition of Depressive Symptoms on Videotaped Interviews of Depressed Cancer Patients: Implications for Designing a Training Program. J. *Pain Symptom Manag.* **2000**, *19*, 329–338.
63. Kim, H.S.; Hwang, S.Y.; Oh, E.G.; Lee, J.E. Development and Evaluation of a PBL-based Continuing Education for Clinical Nurses: A Pilot Study. *J. Korean Acad. Nurs.* **2006**, *36*, 1308, doi:10.4040/jkan.2006.36.8.1308.
64. Bakker, D.; Butler, L.; Fitch, M.; Green, E.; Olson, K.; Cummings, G. Canadian Cancer Nurses' Views on Recruitment and Retention. *J. Nurs. Manag.* **2010**, *18*, 205–214, doi:10.1111/j.1365-2834.2009.01029.x.
65. Fukui, S.; Ogawa, K.; Yamagishi, A. Effectiveness of Communication Skills Training of Nurses on the Quality of Life and Satisfaction with Healthcare Professionals among Newly Diagnosed Cancer Patients: A Preliminary Study. *Psycho-Oncology* **2011**, *20*, 1285–1291, doi:10.1002/pon.1840.
